# Metagenomic insights into viral and microbial genes of Russian High-Arctic soil microbiomes

**DOI:** 10.1038/s42003-026-10050-0

**Published:** 2026-04-15

**Authors:** Beat Frey, Gilda Varliero, Joel Rüthi, Ivan Alekseev, Weihong Qi, Vasiliy Povazhnyi, Vitalii Zemlianskii, Beat Stierli, Ksenia Ermokhina, Gabriela Schaepman-Strub, Jessica Cuartero

**Affiliations:** 1 Swiss Federal Institute for Forest, Snow and Landscape Research WSL, Birmensdorf, Switzerland; 2 Arctic and Antarctic Research Institute, Saint Petersburg, Russia; 3 Laboratory for Greenhouse Gas Monitoring, Karelian Research Centre of the RAS, Petrozavodsk, Russia; 4 Functional Genomics Center Zurich, ETH Zurich and University of Zurich, Zurich, Switzerland; 5 Swiss Institute of Bioinformatics SIB, Geneva, Switzerland; 6 South Scientific Centre of Russian Academy of Science, Rostov-on-Don, Russia; 7 Department of Evolutionary Biology and Environmental Studies, UZH, Zurich, Switzerland; 8 A.N. Severtsov Institute of Ecology and Evolution, Russian Academy of Sciences, Moscow, Russia

**Keywords:** Microbial ecology, Ecosystem ecology

## Abstract

High-Arctic soils are extreme ecosystems where microbial and viral roles remain poorly studied. Climate-driven vegetation expansion may alter these environments, but its impact is unknown. We generate a shotgun metagenomic database from four High-Arctic islands, comparing vegetated and unvegetated sites at two depths (0–2 cm and 30–50 cm). We analyse the functional gene potential, including biosynthetic gene clusters (BGCs) and antibiotic resistance genes (ARGs) in metagenome-assembled genomes (MAGs), and assess viral diversity. Vegetated soils at 30–50 cm were enriched in genes for carbon/nitrogen cycling, energy production, and carbohydrate metabolism, indicating enhanced nutrient inputs. Conversely, unvegetated soils show higher BGC and ARG richness, reflecting microbial competition under nutrient limitation. Viral richness decreases in surface vegetated soils, while diversity and giant virus (Nucleocytoviricota) abundance increase with depth. These findings reveal how vegetation and soil depth modulate microbiomes and viromes, critical for predicting ecosystem trajectories in a warming world.

## Abstract (web summary)

Shotgun metagenomics shows how climate-driven vegetation expansion could alter microbial functions and viral communities in High-Arctic soils, with depth-dependent shifts in nutrient cycling, microbial competition, and giant virus abundance.

## Introduction

The Arctic’s polar desert biome is distinguished by extreme environmental conditions, rendering it particularly sensitive to climate change^1^. Characterised by a mean July air temperature slightly above freezing (0–3 °C), continuous permafrost, shallow snow cover, and persistent summer fog^2,3^, this biome supports discontinuous vegetation with cryptogams as dominant flora. Currently, the Russian High Arctic remains amongst the least explored terrestrial environments on Earth^4^. The Russian High-Arctic archipelagos form a mosaic of islands with diverse landscapes shaped by remote location, geological history and climate. These islands serve as unique natural laboratories for studying microbial adaptations to extreme conditions in relatively isolated environments with minimal anthropogenic influence.

Vegetation cover varies markedly across these islands–some support sparse, cryptogam-dominate plant communities while others remain barren. This variability is particularly relevant: vegetation significantly alters soil microbial functionality and taxonomic composition in High-Arctic areas^5,6^, while barren areas experience faster permafrost degradation due to the limited thermal buffering by organic matter and near-surface vegetation^7^. Soil microbial communities play a critical role in these permafrost regions, particularly through greenhouse gas emissions (CO_2_ and CH_4_)^8^ and functional adaptations enabling survival in extreme conditions, such as lipid transport modifications and enhanced cellular defence mechanisms^5^. Despite this ecological importance, no direct studies have been conducted on Russian Arctic islands–a knowledge gap that demands attention as climate change accelerates.

Biosynthetic gene clusters (BGCs) are contiguous genomic regions encoding enzymatic machinery for specialized metabolites–antibiotics, pigments, toxins and compounds essential for cellular processes and environmental adaption^9–12^. BGCs hold biotechnological value as source of therapeutic natural products^13^. Microorganisms additionally carry antimicrobial resistance genes (ARGs) conferring tolerance to antibiotics. Though traditionally linked to clinical settings, ARGs have also been detected in remote pristine environments, suggesting antibiotic resistance is an ancient, natural microbial trait^14^. In extreme ecosystems, ARGs likely provide selective advantages through mediating competition in nutrient-limited conditions. Understanding environmental ARG prevalence is essential, as they constitute part of the broader environmental resistome—a potential reservoir for pathogenic bacteria^15,16^. Nutrient availability shapes BGC and ARG abundance: oligotrophic environments increase bacterial competition, elevating BGC and ARG prevalence, while nutrient-rich environments favour cooperation and reduce the abundance of these genes^17^.

Cold environments harbour diverse viral communities critical for shaping soil microbial functions and ecosystem processes^18^, yet remain understudied. As obligate parasites, viruses regulate microbial abundance and diversity—key drivers of nutrient cycling^19^. Positive correlation between bacterial and viral abundance indicates a close ecological relationship^20^. Soil viral diversity is strongly influenced by vegetation cover, organic matter, soil temperature, and especially soil moisture^21^. By shaping viral populations, these factors indirectly influence microbial communities, as viral infection redirects host metabolism toward viral replication^22^. Lytic phages cause host lysis, while non-lytic viruses impair host growth^23^. Through modulating microbial populations and interactions, viruses indirectly influence carbon and nitrogen cycling; however, viral diversity in permafrost habitats remains poorly characterised.

Shotgun metagenomic sequencing allows comprehensive permafrost soil microbiome characterisation, including the resistome and virome assessment^5,24^. This approach simultaneously captures taxonomic composition and functional gene potential^25,26^, detecting bacteria, archaea, fungi and viruses^27^. Metagenome-assembled genomes (MAGs) enable assignment of functional traits–BGCs and ARGs–to uncultivated lineages, offering unique opportunities to explore microbial diversity, functionality, adaptive mechanisms, and discover novel bioactive compounds and ARGs.

Accessing the Russian High Arctic islands is logistically challenging due to remoteness, harsh conditions and often military access in the Franz Josef Land, Novaya Zemlya, and Severnaya Zemlya archipelagos^28,29^. The Arctic Century Expedition (2021) enabled the first comprehensive soil samples from these regions^30^. Here, we studied the functional gene potential and microbial diversity at two soil depths (0–2 cm and 30–50 cm, above the permafrost table) on both unvegetated (Graham-Bell, Komsomolets) and vegetated (October Revolution, Vize) High-Arctic islands, representative of Eurasian Subzone A^2^. Our objectives were to: (1) compare the taxonomic and functional potential of the soil microbiome between vegetation cover and soil depths; (2) reconstruct the MAGs and link key traits—such as metabolic genes, ARGs, and BGCs—to specific taxa; and (3) characterise the virome composition.

We hypothesized that:

1. vegetated islands harbour higher taxonomic diversity and protein-coding gene (functional gene potential) diversity than unvegetated islands, with the highest microbial and functional diversity occurring at 0–2 cm soil depth, due to the greater availability of energy (C) and nutrients (e.g. plant litter input).
2. MAGs from vegetated islands (especially at 0–2 cm depth) have a higher abundance of genes involved in carbon (C) and nitrogen (N) cycling but fewer BGCs and ARGs than those from unvegetated islands, due to lower microbial competition for C and N.
3. Vegetation cover leads to higher bacterial abundance (mainly at 0–2 cm soil depth), resulting in higher viral diversity on vegetated islands.

## Results

### Vegetated surface soils have more organic matter and higher microbial abundance

**Fig. 1:**
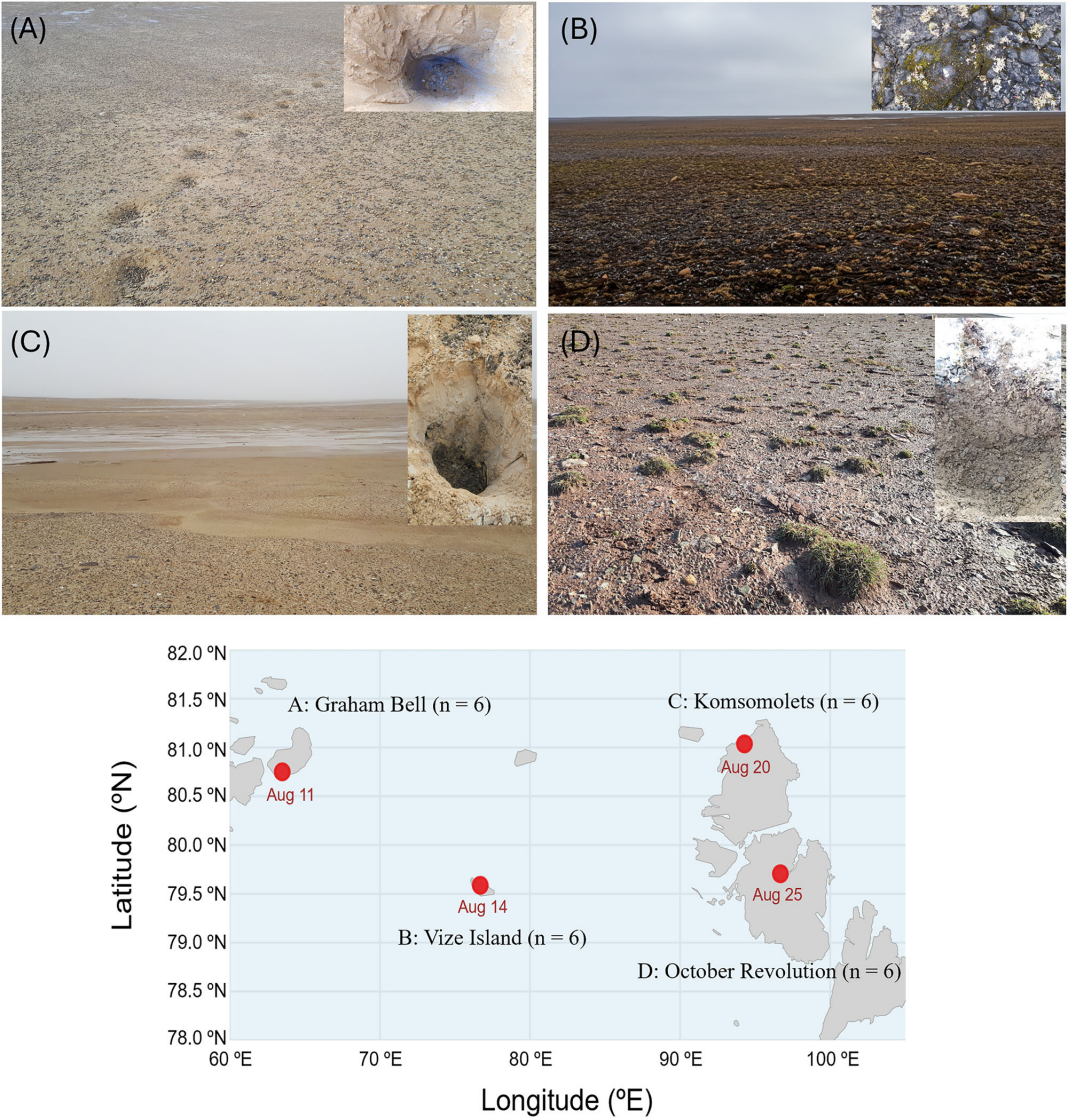
Sampling sites across the Russian High-Arctic. Study sites in the Russian High-Arctic. Photographs show representative landscapes of the sampled locations: **A** Graham Bell, **B** Vize Island, **C** Komsomolets, and **D** October Revolution, including close-up views of surface soils. The map indicates the geographic locations of the four islands, sampling sites (red dots), sampling dates, and the total number of samples collected at each location. Source data are provided in Supplementary Data 1.

Compared to unvegetated islands (Fig. 1, and Supplementary Figs. 1 and 2), the vegetated islands (October Revolution and Vize Island; Fig. 1, and Supplementary Figs. 3 and 4) have larger diurnal temperature ranges and higher temperatures during the summer months (Supplementary Table 1). These conditions may contribute to the presence of vegetation, with Vize supporting greater plant diversity and cover of vascular plants (graminoids and forbs) and cryptogams (bryophytes and lichens), likely due to its higher precipitation and temperature values (Supplementary Table 1, and Supplementary Fig. 4). On October Revolution, vegetation is present but very scarce and mostly consists of vascular plants (Supplementary Table 1, and Supplementary Fig. 3). At the soil surface, vegetated islands showed significantly higher CO₂ fixation and lower CO₂ emissions compared with unvegetated islands (Supplementary Table 2). Vegetation cover significantly increased soil organic carbon (SOC), DNA, and ITS rRNA copy numbers in soils (all *T*_*v*_ > 3, *P* < 0.05; Table 1), although its effect was greater for surface soils (all *T*_*vs*_ < −3, *P* < 0.05; Table 1). 16S rRNA copy numbers were significantly smaller at greater soil depth (Table 1). Soil depth significantly affected silt and sand, showing a higher content with higher soil depth (Table 1).

**Table 1:** Soil physico-chemical and biological properties of the studied High-Arctic islands

	pH	Clay (%)	Silt (%)	Sand (%)	SOC (%)	DNA (µg g^-1^ dry soil)	16S rRNA copies	ITS rRNA copies
0–2 cm								
G	7.6 ± 0.6	7.9 ± 1.9	30.0 ± 1.84	62.0 ± 3.4	0.8 ± 0.2	3.2 ± 1.5	4.6×10^10^ ± 2.1×10^10^	2.5×10^7^ ± 1.6×10^7^
K	5.1 ± 0.7	9.1 ± 2.6	30.4 ± 0.7	60.5 ± 2.1	0.2 ± 0.1	1.9 ± 0.4	2.0×10^10^ ± 1.5×10^10^	1.8×10^7^ ± 1.2×10^7^
O	7.0 ± 0.3	10.4 ± 5.1	30.0 ± 2.0	59.5 ± 7.1	6.2 ± 3.2	11.8 ± 3.0	1.9×10^10^ ± 2.5×10^10^	22.8×10^7^ ± 15.2×10^7^
V	6.3 ± 0.9	13.7 ± 2.5	28.7 ± 1.1	57.6 ± 2.1	7.1 ± 0.4	50.1 ± 13.9	2.9×10^10^ ± 4.2×10^10^	10.6×10^7^ ± 11.9×10^7^
30–50 cm								
G	7.7 ± 0.3	14.0 ± 3.88	30.4 ± 2.0	55.6 ± 2.6	1.6 ± 1.5	1.9 ± 1.7	7.23×10^8^ ± 0.8×10^8^	8.1×10^4^ ± 5.0×10^4^
K	6.0 ± 0.2	8.3 ± 1.4	32.5 ± 1.4	59.2 ± 2.5	0.3 ± 0.1	< 0.01	13.2×10^8^ ± 13.4×10^8^	31.2×10^4^ ± 48.9×10^4^
O	7.3 ± 0.6	10.9 ± 0.4	31.0 ± 1.1	58.0 ± 0.6	2.3 ± 0.7	4.6 ± 2.6	4.8×10^8^ ± 2.5×10^8^	49.1×10^4^ ± 4.7×10^4^
V	6.5 ± 0.4	9.4 ± 2.4	29.5 ± 1.9	61.2 ± 2.24	1.3 ± 0.1	1.6 ± 0.5	7.2×10^8^ ± 2.9×10^8^	29.8×10^4^ ± 12.4×10^4^
	*T*_*v*_ = 0.31 *P*_*v*_ = 0.84 *T*_*s*_ = 1.60 *P*_*s*_ = 0.14 *T*_*vs*_ = −0.67 *P*_*vs*_ = 0.56	*T*_*v*_ = 1.95 *P*_*v*_ = 0.07 *T*_*s*_ = 1.45 *P*_*s*_ = 0.16 *T*_*vs*_ = −1.75 *P*_*vs*_ = 0.10	*T*_*v*_ = 1.00 *P*_*v*_ = 0.40 ***T***_***s***_ = **−2.55** ***P***_***s***_ = **0.020** *T*_*vs*_ = −1.00 *P*_*vs*_ = 0.33	*T*_*v*_ = 1.38 *P*_*v*_ = 0.27 ***T***_***s***_ = **−2.65** ***P***_***s***_ = **0.017** *T*_*vs*_ = −1.40 *P*_*vs*_ = 0.18	***T***_***v***_ = **5.92 *P***_***v***_ = **0.012** *T*_*s*_ = 1.00 *P*_*s*_ = 0.34 ***T***_***vs***_ = **−7.63** ***P***_***vs***_ < **0.001**	***T***_***v***_ = **3.43 *P***_***v***_ = **0.036 ** *T*_*s*_ = −0.44 *P*_*s*_ = 0.70 ***T***_***vs***_ = **−3.59** ***P***_***vs***_ = **0.002**	*T*_*v*_ = −2.22 *P*_*v*_ = 0.06 ***T***_***s***_ = **−3.93** ***P***_***s***_ < **0.001** *T*_*vs*_ = 1.97 *P*_*vs*_ = 0.06	***T***_***v***_ = **4.58 *P***_***v***_ < **0.001** *T*_*s*_ = −0.90 *P*_*s*_ = 0.42 ***T***_***vs***_ = **−3.43** ***P***_***vs***_ < **0.001**

Effects of vegetation cover and soil depth on soil physico-chemical and biological properties, presented as means ± standard deviation (*N* = 21*; n* = *3*). Statistical significance was assessed using linear mixed-effects models with vegetation (*v*), soil depth (*s*), and their interaction (*vs*) as fixed effects. *T-*values indicate effect direction and magnitude (positive/negative relationships). Unvegetated: Graham Bell (G), Komsomolets (K); vegetated: October Revolution (O), Vize Island (V). SOC: soil organic carbon concentration (%). Significant *P*-values are in bold.

### Vegetation cover and soil depth affect microbial taxonomic diversity and composition

**Fig. 2:**
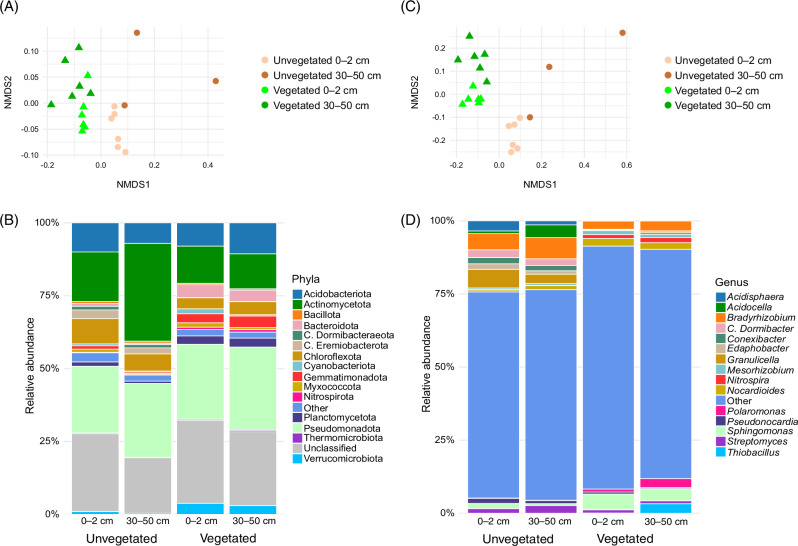
Soil microbial communities. Non-metric multidimensional scaling (NMDS) plots based on Bray–Curtis dissimilarity, showing beta diversity at the phylum (**A**) and genus (**C**) levels (*N* = 21; *n* = 3). Bar plot displaying the relative abundances (CPMs) of the 15 most abundant taxa at the phylum (**B**) and genus (**D**) levels. Unvegetated: Graham Bell (G), Komsomolets (K); vegetated: October Revolution (O), Vize Island (V). Source data are provided in Supplementary Data 2–5.

Microbial richness and Shannon index were higher in vegetated surface soils compared with unvegetated surface soils (Supplementary Table 3). Vegetation cover and soil depth both significantly affected microbial community structure (*F*_*v*_ = 22.12; *P* = 0.003; *P*_*s*_ = 4.59; *P* = 0.002). At the kingdom level, Eukaryota abundance (CPM) was higher at 0–2 cm depth, while bacterial abundance was higher at 30–50 cm on unvegetated islands and at 0–2 cm on vegetated islands (Table 2). At the phylum level, both vegetation cover and soil depth significantly affected community structure (Fig. 2A; vegetation cover: *F* = 12.9, *P* = 0.010; soil depth: *F* = 4.21, *P* = 0.006). Among 228 identified phyla, Verrucomicrobiota, Planctomycetota, and Cyanobacteriota showed higher abundances on vegetated islands, while Pseudomonadota, Bacillota, Candidatus Dormibacteraeota, and Bacteroidota were lower (Fig. 2B, and Supplementary Table 4). Acidobacteriota, Chloroflexota, Nitrospirota, and Thermomicrobiota had highest abundances at 30–50 cm on vegetated islands, whereas Cyanobacteriota and Actinomycetota were lowest in this environment (Fig. 2B, and Supplementary Table 4). At the genus level, both vegetation cover and soil depth had a significant effect on microbial community structure (Fig. 2C; vegetation cover: *F* = 15.6, *P* = 0.017; soil depth: *F* = 2.66, *P* = 0.031). The genera *Bradyrhizobium*, *Conexibacter*, and *Candidatus Dormibacter* were significantly less abundant on vegetated islands, while *Nocardioides*, *Polaromonas*, and *Nitrospira* had higher abundances on these islands (Fig. 2D, and Supplementary Table 5). On unvegetated islands*, Mesorhizobium*, *Acidocella*, and *Edaphobacter* had higher abundances in surface soil, whereas *Conexibacter* and unclassified taxa were more abundant (CPM) in deeper soil (Fig. 2D, and Supplementary Table 5).

**Table 2:** Soil microbial taxa composition of the studied High-Arctic islands

	Archaea	Bacteria (×10^3^)	Eukaryota (×10^2^)	Viruses
0–2 cm				
G	1538 ± 166	741 ± 7	411 ± 141	259 ± 59
K	1200 ± 90	745 ± 18	2262 ± 397	441 ± 73
O	3827 ± 2,988	745 ± 14	181 ± 66	471 ± 107
V	1052 ± 144	723 ± 17	347 ± 39	195 ± 40
30–50 cm				
G	1490 ± 175	830 ± 27	199 ± 189	351 ± 171
O	2183 ± 512	773 ± 17	94 ± 9	769 ± 145
V	3148 ± 1706	759 ± 39	122 ± 25	285 ± 122
	*T*_*v*_ = 0.12 *P*_*v*_ = 0.91 *T*_*s*_ = 0.22 *P*_*s*_ = 0.83 *T*_*vs*_ = 1.50 *P*_*vs*_ = 0.15	*T*_*v*_ = −0.50 *P*_*v*_ = 0.64 ***T***_***s***_ = **5.66** ***P***_***s***_ < **0.001** ***T***_***vs***_ = **−2.80** ***P***_***vs***_ = **0.012**	*T*_*v*_ = −1.16 *P*_*v*_ = 0.37 ***T***_***s***_ = **−2.57** ***P***_***s***_ = **0.024** *T*_*vs*_ = 0.58 *P*_*vs*_ = 0.57	*T*_*v*_ = −1.16 *P*_*v*_ = 0.37 *T*_*s*_ = −1.58 *P*_*s*_ = 0.14 *T*_*vs*_ = 0.39 *P*_*vs*_ = 0.71

Effects of vegetation cover and soil depth on abundance (counts per million, CPM) across microbial taxonomic kingdoms, presented as means ± standard deviation (*N* = 21*; n* = *3*). Statistical significance was assessed using linear mixed-effects models with vegetation (*v*), soil depth (*s*), and their interaction (*vs*) as fixed effects. *T-*values indicate effect direction and magnitude (positive/negative relationships). Unvegetated: Graham Bell (G), Komsomolets (K); vegetated: October Revolution (O), Vize Island (V). Significant values are in bold. Insufficient DNA for shotgun metagenomic sequencing was extracted from soil samples from Komsomolets at a depth of 30–50 cm, so soils at this depth from unvegetated islands are solely represented by soil samples collected from Graham Bell.

Although microbial alpha-diversity did not differ significantly among the studied environments, the highest taxonomical richness was found in unvegetated surface soils, while on vegetated islands, richness was higher in deeper soils. Microbial community structure was significantly affected by vegetation cover and soil depth, with taxa like *Bradyrhizobium* (Pseudomonadota) and *Conexibacter* (Actinomycetota) being more abundant on unvegetated islands, and *Nitrospira* (Nitrospirota) on the vegetated ones.

### Strong impact of vegetation cover on the functional gene potential of the soil microbiome

**Fig. 3:**
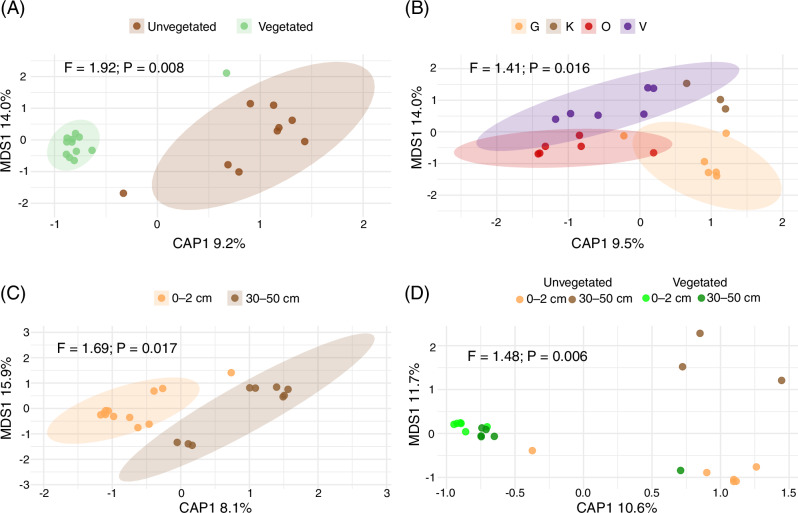
Vegetation and soil depth shape functional gene composition. Effects of vegetation cover, soil depth on functional gene structure, represented through canonical analysis of principal coordinates (CAP) using the Bray–Curtis distance (*N* = 21; *n* = 3) of **A** vegetation cover (vegetated vs. unvegetated), **B** the studied islands **C** soil depth and **D** the interaction between the vegetation cover and soil depth. The plots display the “sites” points, which include the first CAP axis and MDS1. Unvegetated: Graham Bell (G), Komsomolets (K); vegetated: October Revolution (O), Vize Island (V). Source data are provided in Supplementary Data 6.

Functional gene structure was significatively affected by vegetation cover and soil depth (Fig. 3; vegetation cover: *F* = 1.92, *P* = 0.008; soil depth: *F* = 1.69, *P* = 0.017), and this functional gene structure was driven by pH at both soil depths and by SOC content at 0–2 cm (Supplementary Table 6). Functional gene diversity was also significantly affected by soil depth, with both richness and Shannon index being significantly higher at 0–2 cm depth compared with deeper soils (Table 3). The abundances (CPM) of protein-coding genes of the super-categories of cellular processes and signalling (CPS), information storage and processing (ISP), and metabolism were not significantly affected by vegetation cover alone. However, a significant interaction between vegetation cover and soil depth indicated higher abundances of these categories in deeper soil (Table 4, and Supplementary Table 7). On unvegetated islands, the abundance of these eggNOG super-categories was higher in surface soils. Detailed information about the effect of vegetation cover and soil depth on eggNOG classes can be found in Table 4, Supplementary Results 1, and Supplementary Tables 8–10.

**Table 3:** Functional gene diversity of the studied High-Arctic islands

Functional gene diversity		
	Richness (×10^5^)	Shannon index
0–2 cm		
G	12.4 ± 1.0	13.7 ± 0.1
K	10.4 ± 0.7	13.4 ± 0.1
O	13.6 ± 12.6	13.8 ± 0.1
V	15.5 ± 0.5	14.1 ± 0.1
30–50 cm		
G	7.4 ± 3.3	12.7 ± 0.7
O	8.6 ± 0.4	12.5 ± 0.8
V	10.0 ± 29.7	13.1 ± 0.8
	*T*_*v*_ = 2.22 *P*_*v*_ = 0.10 ***T***_***s***_ = **-3.35** ***P***_***s***_ = **0.011** *T*_*vs*_ = -0.47 *P*_*vs*_ = 0.64	*T*_*v*_ = 1.57 *P*_*v*_ = 0.14 ***T***_***s***_ = **-2.74** ***P***_***s***_ = **0.014** *T*_*vs*_ = -0.14 *P*_*vs*_ = 0.89

Effects of vegetation cover and soil depth on functional gene diversity (richness and Shannon diversity index). Values of alpha-diversity are presented as means ± standard deviation (*N* = 21*; n* = *3*). Statistical significance was assessed using linear mixed-effects models with vegetation (*v*), soil depth (*s*), and their interaction (*vs*) as fixed effects. *T*-values indicate effect direction and magnitude (positive/negative relationships). Unvegetated: Graham Bell (G), Komsomolets (K); vegetated: October Revolution (O), Vize Island (V). Significant values are in bold. Insufficient DNA for shotgun metagenomic sequencing was extracted from soil samples from Komsomolets at a depth of 30–50 cm, so soils at this depth from unvegetated islands are solely represented by soil samples collected from Graham Bell.

**Table 4:** Summary of the effects of vegetation cover and soil depth on the functional genetic potential

Database	Functional category/substrate/class/family	*T*_*v*_	*T*_*s*_	*T*_*vs*_
eggNOG super-categories	Cellular processing and signalling (CPS)	↓	↓**	↑***
	Information storage and processing (ISP)	↓	↓**	↑**
	Metabolism (MBM)	↓	↓**	↑***
eggNOG functional categories	Amino acid transport and metabolism (E)	↓	↓*	↑***
	Carbohydrate transport and metabolism (G)	↓	↓*	↑***
	Cell wall/membrane/envelope biogenesis (M)	↓	↓*	↑***
	Cytoskeleton (Z)	↓	↓	↑**
	Defence mechanisms (V)	↓	↓*	↑***
	Energy production and conversion (C)	↓	↓*	↑***
	Function unknown (S)	↓	↓	↑*
	Lipid transport and metabolism (I)	↓	↓**	↑***
	Post-translational modification, protein turnover, and chaperones (O)	↓	↓**	↑***
	Replication, recombination, and repair (L)	↓	↓	↑**
	Secondary metabolites biosynthesis, transport and catabolism (Q)	↓	↓**	↑***
	Transcription (K)	↓	↓**	↑***
	Translation, ribosomal structure, and biogenesis (J)	↓	↓**	↑***
CAZy classes	Auxiliary activities (AA)	↓	↓	↑**
	Carbohydrate binding modules (CBM)	↓	↓	↑***
	Carbohydrate esterases (CE)	↓	↓*	↑***
	Glycoside hydrolases (GH)	↓	↓*	↑***
	Polysaccharide lyases (PL)	↓	↓	↑**
	Glycosyl transferases (GT)	↓	↓**	↑***
CAZy substrates	Cellulose – (CBM, GH)	↓	↓*	↑***
	Chitin – (CBM, GH, CE)	↓	↓*	↑***
	Hemicellulose – (CBM, GH, CE)	↓	↓*	↑***
	Lignin – (CBM, GH, AA)	↓	↓	↑*
	Multiple – (CBM, GH, CE)	↓	↓*	↑***
	Murein – (GH)	↓	↓*	↑***
	Oligosaccharides – (CBM, GH, CE, PL)	↓	↓*	↑***
	Pectin – (CBM, CE, GH, PL)	↓	↓	↑***
	Starch – (CBM)	↓	↓	↑***
NCyc families	Anammox (A)	↓	↓	↑
	Assimilatory nitrate reduction (ANR)	↓	↓*	↑***
	Denitrification (D)	↑	↓	↑***
	Denitrification and dissimilatory nitrate reduction (DDNR)	↑	↑***	↑***
	Dissimilatory nitrate reduction (DNR)	↓	↓	↑***
	Nitrification (N)	↑	↓	↑*
	N_2_-fixation (NF)	↓	↓**	↑*
	Organic degradation and synthesis (OD&S)	↓	↓	↑***

Effects of vegetation cover and soil depth on the abundance (counts per million, CPM) of EggNOG super-categories and categories, CAZyme substrates and classes, and NCycDB families (*N* = 21; *n* = 3). Statistical significance was assessed using linear mixed-effects models with vegetation (*v*), soil depth (*s*), and their interaction (*vs*) as fixed effects. ^*^P < 0.05; ^**^P < 0.01; ^***^P < 0.001. ↑ and ↓ represent the direction of effect in the model. ↑*T*_*v*_ indicates a positive correlation with vegetation cover, ↓*T*_*s*_ indicates a negative correlation with soil depth, and ↑*T*_*vs*_ indicates a larger effect of vegetation cover at greater soil depth. Data are from shotgun metagenomic sequencing.

The abundances (CPM) of protein-coding gene classes annotated by the CAZy database and associated with catabolic processes—including auxiliary activities (AA), carbohydrate esterases (CE), carbohydrate-binding modules (CBM), glycoside hydrolases (GH), and polysaccharide lyases (PL)—and those involved in anabolic processes, such as glycosyl transferases (GT), were not affected by vegetation cover alone. However, a significant positive interaction between vegetation cover and soil depth was observed, indicating a higher abundance of AA, CE, CBM, GH, and PL classes in vegetated soils at 30–50 cm depth (Table 4, and Supplementary Table 11). CE, GH, and GT had higher abundances on unvegetated islands at 0–2 cm depth. Similarly, the abundance of genes involved in the breakdown of various C substrates, such as cellulose, chitin, hemicellulose, lignin, multiple sources, murein, oligosaccharides, pectin, and starch, was higher on vegetated islands in deeper soils (Table 4, and Supplementary Table 12). On unvegetated islands, the abundance of genes acting on cellulose, chitin, hemicellulose, multiple, murein, and oligosaccharides was higher in surface soils.

The abundances (CPM) of gene families annotated by the NCyc database showed a similar pattern, with higher abundances of genes related to assimilatory nitrate reduction (ANR), denitrification and dissimilatory nitrate reduction (DDNR), dissimilatory nitrate reduction (DNR), and organic degradation and synthesis (OD&S) on vegetated islands at 30–50 cm depth (Table 4, and Supplementary Table 13). On unvegetated islands, the abundance of genes related to ANR and N_2_-fixation was higher in surface soils. Although statistical significance was not assessed due to the low number of samples, a comparison of O and V samples, selected based on differences in vegetation cover, is provided in Supplementary Table 14.

These results highlight the strong impact of vegetation cover on the studied environments, particularly at greater soil depths. In vegetated islands, the abundance of genes related to overall metabolism, C and N cycling, substrate catabolism, microbial growth, and defence was significantly higher in deeper, cryoturbated permafrost soils.

### Metabolic versatility in High-Arctic metagenome-assembled genomes (MAGs)

A total of 230 MAGs were reconstructed across the four islands and two soil depths (≥ 50% completeness, < 10% contamination; a complete list of the MAGs is available in the Supplementary Data 17). Among these, we identified a total of nine potential novel taxa based on thresholds of < 95% ANI and > 60% AF (Supplementary Tables 15–18). None of the MAGs was exclusively associated with a specific island, vegetation cover, or soil depth. Therefore, we used LEfSe analysis (*P* < 0.01) to identify MAGs that were associated with a particular environment (Supplementary Fig. 5), which revealed a predominance of Chloroflexota and Eremiobacterota on unvegetated islands.

**Fig. 4:**
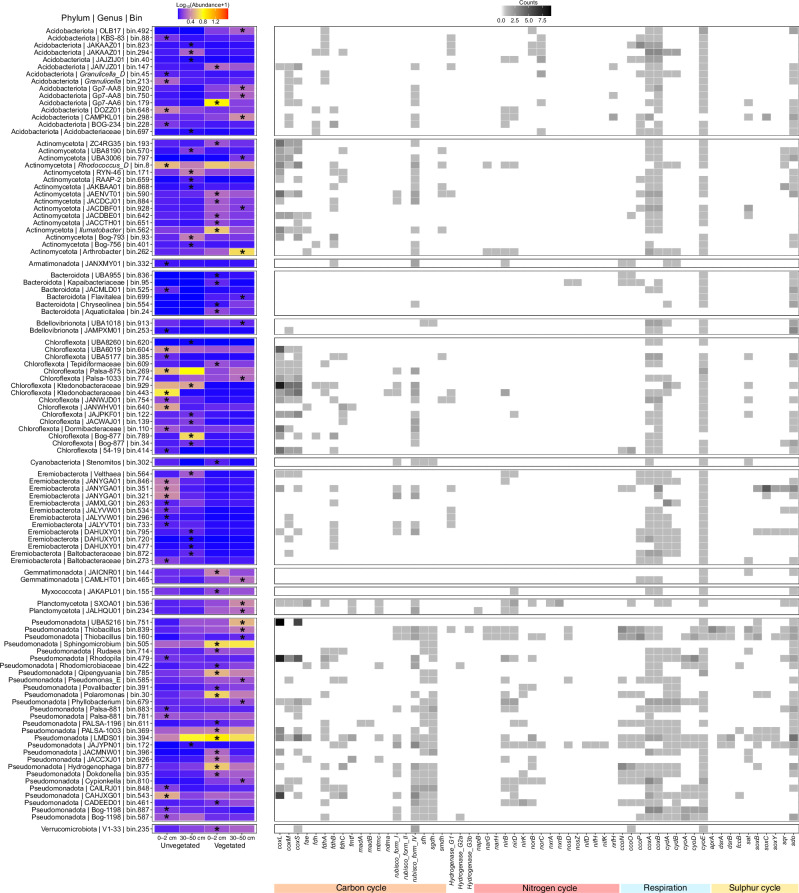
Metagenome-assembled genome distribution across soil environments. Heatmap showing the effect of vegetation cover and soil depth on the log-transformed relative abundance (colour scale) of metagenome-assembled genomes (MAGs) across soil environments, with gene hit counts (in grey) detected by metabolisHMM (*N* = 21; *n* = 3). Only MAGs with > 87% completeness and < 10% contamination are shown. Asterisks in the colour scale indicate MAG indicators (*P* < 0.01). When genus-level classification was not possible, the name of the finest confidently assigned taxonomic rank is provided. Source data are provided in Supplementary Data 7-8.

Functional annotation of selected high-quality MAGs (> 87% completeness) revealed consistent patterns across the islands and soil depths, with widespread potential for CO_2_ fixation via Rubisco forms I and IV and for CO oxidation through *coxLMS* genes (Fig. 4). Genes involved in N and sulphur (S) cycling were more sporadically distributed. Denitrification pathways appeared incomplete in all the environments, although some specific MAGs (especially on vegetated islands) harboured *narGH*, *nirK*, *norBC*, and *nrfH*, suggesting limited potential for denitrification and microbially driven DNR to ammonium. Genes for microbial sulphur metabolism were generally scarce but were more diverse in deeper vegetated soils, where complete pathways (*sat*, *dsrAB*, *sox*genes) co-occurred in a few MAGs. Terminal oxidases for both high- and low-oxygen conditions (e.g. *ccoPON*, *cydA*, *cyoE*, *coxAB*) were found in all environments, indicating respiratory versatility. A full description of the annotated genes for each MAG and environment is available in Supplementary Results 2–5. The phyla with the largest number of functional genes were *Pseudomonadota* and *Planctomycetota*, which included genera such as *Thiobacillus*, *Rudaea*, *Hydrogenophaga*, and *Cypionkella*, all of which were associated with vegetated islands (LEfSe, *P* < 0.01; Fig. 4, and Supplementary Fig. 5). Unvegetated soils had a lower representation of functional genes related to C, N, and S metabolism and respiration, particularly within the phyla *Eremiobacterota* and *Armatimonadota* (Fig. 4).

Our findings suggest that, despite potentially higher nutrient availability on vegetated islands, the functional gene potential of the identified MAGs was not clearly distinct between unvegetated and vegetated soils. While certain phyla with small numbers of functional genes—such as *Eremiobacterota* and *Armatimonadota*—were mostly associated with unvegetated islands, on the vegetated islands, we identified MAGs, such as *Gemmatimonadota*, that lacked genes involved in C and N cycling.

### BGC and ARG in High-Arctic soil metagenome assemblages

**Fig. 5:**
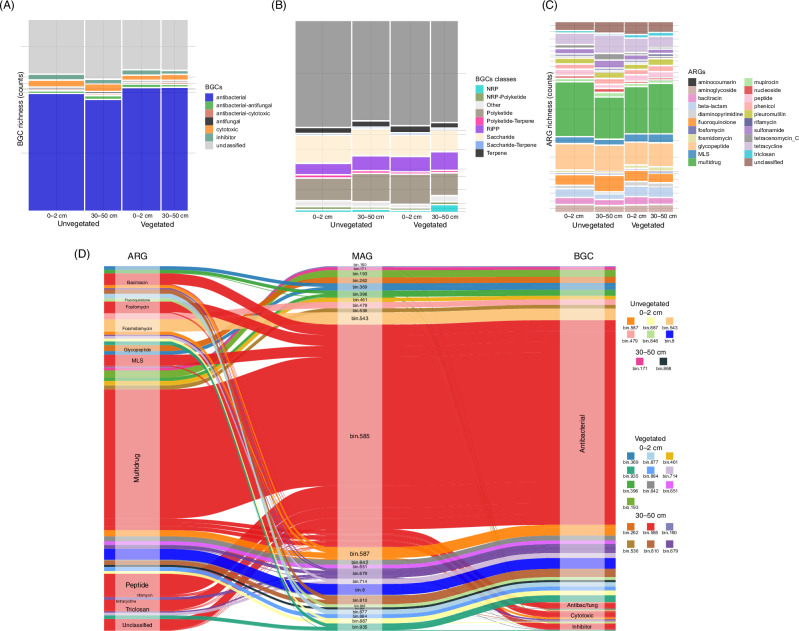
Biosynthetic and antimicrobial resistance gene diversity. Stacked barplot displaying the effect of vegetation cover and soil depth on BGC and ARG richness (defined as the number of genes encoding BGCs and ARGs) for: **A** biosynthetic gene clusters (BGCs), **B** BGC products, and **C** antimicrobial resistance genes (ARGs). An alluvial plot **D** displays the relationships among ARG richness, metagenome-assembled genomes (MAGs), and BGCs (*N* = 21). Unvegetated: Graham Bell (G), Komsomolets (K); vegetated: October Revolution (O), Vize Island (V). Source data are provided in Supplementary Data 9 and 10.

Considering only MAGs (LEfSe *P* < 0.01; > 87% completeness), we found the largest number of BGCs richness (defined as the number of genes encoded within BGCs) in surface soils (0–2 cm depth), with 1115 BGCs on unvegetated and 762 BGCs on vegetated islands. Deeper soils (30–50 cm depth) contained fewer BGCs (unvegetated = 723; vegetated = 522). The most common predicted BGCs were antimicrobial, cytotoxic, and inhibitory compounds, primarily from non-ribosomal peptides (NRPs), polyketides, ribosomally synthesized and post-translationally modified peptides (RiPPs), saccharides, and terpenes. Over 55% of BGCs across all soil environments remained unclassified, indicating strong potential for novel bioactive molecule discovery (Fig. 5A, B, and Supplementary Table 19). ARGs richness (defined as the number of ARG-encoding genes) and the diversity of ARGs were highest in unvegetated surface soils and lowest in vegetated surface soils. Across all locations, multidrug resistance genes were the most abundant (higher ARGs richness), followed by glycopeptide and tetracycline resistance genes—particularly in unvegetated and deeper soils (Fig. 5C, and Supplementary Table 20). ARGs for beta-lactams, bacitracin, and fluoroquinolones were present in all the studied soil environments. Unclassified ARGs accounted for a significant fraction in each environment. Some MAGs showed a particularly large number of BGCs and ARGs (Fig. 5D), such as MAG 585 (an indicator of deeper vegetated soils), and MAGs 543 and 587 (indicators of vegetated surface soils). Although all the studied environments had a large number of BGCs and ARGs, the highest BGCs and ARGs richness was observed in unvegetated surface soils, suggesting strong microbial competition for available resources. Interestingly, MAG 585 (*Pseudomonas*), which had the largest ARGs richness, was found in deeper vegetated soils.

### Vegetation cover and soil depth change the soil virome

**Fig. 6:**
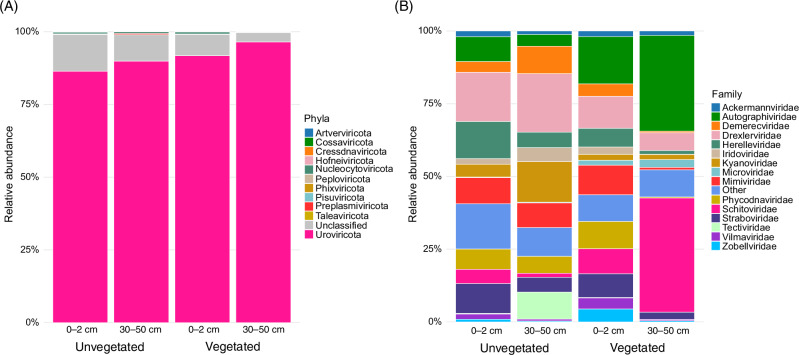
Taxonomic composition of the Arctic soil virome. Stacked Bar plot displaying the relative abundance (CPMs) in the soil virome at the **A** phylum and **B** family levels (*N* = 21; *n* = 3). The family-level plot includes the 15 most abundant families out of a total of 47 identified. Unvegetated: Graham Bell (G), Komsomolets (K); vegetated: October Revolution (O), Vize Island (V). Source data are provided in Supplementary Data 11 and 12.

The virome structure was significantly affected by vegetation cover and soil depth (Supplementary Fig. 6; vegetation cover: *F* = 2.65, *P* = 0.001; soil depth: *F* = 1.57, *P* = 0.013). At the phylum level, the abundance (CPM) of Pisuviricota (genome: ssRNA+, dsRNA) was higher on unvegetated islands, while that of Taleaviricota (dsDNA) was higher on unvegetated islands at depths of 0–2 cm depth and on vegetated islands at 30–50 cm depth (Fig. 6A, and Supplementary Table 21). Nucleocytoviricota (giant dsDNA viruses) were more abundant on vegetated islands at 0–2 cm depth. At the family level, Herelleviridae (class: Caudoviricetes; dsDNA) and Drexlerviridae (Caudoviricetes; dsDNA) had the highest abundances in unvegetated soils at 0–2 cm (Fig. 6B, and Supplementary Table 22). In contrast, Demerecviridae (Caudoviricetes; dsDNA) and Tectiviridae (Tectiliviricetes; dsDNA) were more abundant at 0–2 cm depth on unvegetated islands but at 30–50 cm depth on vegetated islands, together with Iridoviridae (Megaviricetes; dsDNA; Supplementary Table 23).

These findings indicate that vegetation cover and soil depth significantly affect the High-Arctic soil virome, particularly certain taxa, such as those in the Nucleocytoviricota phylum, which includes the giant virus family Mimiviridae. Although the abundance of Mimiviridae in particular did not differ significantly among the studied environments, its gene counts were higher at 0–2 cm depth on all the islands.

## Discussion

### Effects of vegetation cover and soil depth on soil properties, microbial diversity, and functional potential

Here, we studied four remote High-Arctic soil environments: vegetated and unvegetated islands, each sampled at two soil depths (active surface layer and cryoturbated deeper soils). Previous research has demonstrated that taxonomic diversity in Arctic soils can be as high as that found in temperate and tropical forests^31,32^ and that plant litter can increase the microbial diversity in High-Arctic environments^5^. Based on this, our first hypothesis posited that even sparse vegetation in the Arctic would increase soil C and nutrient availability, leading to greater microbial richness and diversity—especially in the surface layer at 0–2 cm depth. This hypothesis was supported by our results; however, we also observed higher diversity in vegetated islands at depths of 30–50 cm. This may be attributed to the release of iron, silicon, and aluminium from minerals, which are typically more abundant near the soil surface and can increase the microbial diversity compared with that in deeper layers^33^. Notably, qPCR quantification revealed that 16S rRNA gene copy numbers were significantly reduced at greater soil depths, suggesting that while community composition may be resilient to vegetation cover, the bacterial abundance declines substantially with depth, likely reflecting depletion of labile carbon substrates below the active soil layer.

Interestingly, and contrary to our first hypothesis, the highest taxonomic richness on vegetated islands was observed at 30–50 cm depth. This finding is surprising, given that most of the vegetation on the studied islands consists of nonvascular plants, which typically have shallow root systems and rely primarily on atmospheric deposition (nutrient uptake) and environmental factors, such as soil properties^34^. Therefore, two potential sources of nutrient input at lower soil depths could be considered: the release of nutrients after permafrost thaw and the rhizosphere priming. Due to climate change, permafrost soils are thawing at greater soil depths^35^. As permafrost thaws, C and N that were previously frozen are becoming available for microorganisms and plants^36^, potentially contributing to the observed greater gene abundance at 30–50 cm depth. Furthermore, in a study by Blume-Werry et al.^37^, the root systems of vascular plants were found to be closely linked to permafrost thaw, as they access newly released pools of C and N resulting from thawing processes. Through this deeper root penetration and nutrient uptake, plants not only exploit these resources but also actively modify the biogeochemical properties of the soil. For instance, Blume-Werry et al.^37^ found that root litter input at all soil depths was ten times greater with permafrost thaw than in frozen soils. In another study, Proctoc and He^38^ found that tundra plant root exudates release readily degradable compounds—such as organic acids, amino acids, and carbohydrates. This process, known as rhizosphere priming^39^, stimulates microbial activity, thereby enhancing microbial richness and altering the functional potential of the microbial community. Additionally, root presence can alter the physicochemical structure of the soil by increasing pore size and enhancing the physical retention of organic matter, which in turn facilitates the infiltration of microbial enzymes into the soil matrix^40^ and aeration. Moreover, in permafrost soils, processes such as argillaceous mineral accumulation at the active layer–permafrost interface further contribute to changes in soil structure.

On the other hand, plant cell walls are mainly composed of recalcitrant polymers, such as cellulose, hemicellulose, and lignin^41^, which require degradation by specialized soil microorganisms. Accordingly, the higher abundances of genes encoding CBMs and PLs in deeper soils in our study may reflect greater availability of these plant-derived substrates in the deeper soil. Enhanced microbial competition for these resources highlights the strong adaptability and competition among both plants and microorganisms. In addition, chitin—a major component of fungal cell walls and necromass^42^—may serve as a continuous C and nutrient source. The observed depth-dependent enrichment of chitinolytic genes is consistent with findings from forest soils^43^, indicating a general microbial adaptation to the accumulated presence of chitin in deeper soil layers. Interestingly, we also found a greater abundance of genes (gene counts; counts per million) involved in N_2_-fixation in deeper soils. While most studies indicate that N_2_-fixation is more prominent in surface layers, some reports suggest that it can be greater in deeper soils (e.g.^44,45^).

Our results also suggest that deeper (cryoturbated) soils on vegetated islands may represent more copiotrophic conditions compared with the other three soil environments (unvegetated at 0–2 and 30–50 and vegetated at 0–2 cm depth). This is supported by the observed higher abundances of genes associated with microbial adaptation strategies, including cell wall modifications under biotic stress^46^, metabolic reprogramming for nutrient optimization^47^, and enhanced defences against antagonistic interactions. Moreover, the observed higher gene abundances of the eggNOG categories C (energy production and conversion) and Q (secondary metabolite biosynthesis, transport, and catabolism) on vegetated islands are consistent with competitive environments, where microbes invest in the production of secondary metabolites, such as antimicrobials^48^, a process that demands increased energy regeneration. However, metagenomic analysis also revealed that only the bacterial kingdom was significantly affected by vegetation cover and soil depth, consistent with results previously reported in Arctic tundra soils^49^.

Considering only the most abundant taxa, we found that specific bacterial phyla were significantly affected by vegetation cover and soil depth. For example, the abundance of Nitrospirota and Chloroflexota was higher in vegetated soils at 30–50 cm depth. Nitrospirota are essential for soil nitrification (conversion of NH₄⁺ to NO₂⁻ and then to NO₃⁻), playing a key role in increasing N availability for plants in extreme environments^50^. On the other hand, Chloroflexota is known for its ability to degrade recalcitrant organic matter and contribute to C cycling in soils, and it is considered a strong candidate for biotechnological applications due to its versatility and metabolic capabilities^51^. At the genus level, we found that *Conexibacter* was more abundant in vegetated soils at 30–50 cm depth; members of this genus are generally heterotrophic and are associated with nutrient-rich environments, due to their diverse metabolic capabilities. *Acidocella*, an acidophilic and strictly aerobic bacterium, was most abundant in vegetated surface soils, likely due to the well-aerated topsoil layers and the acidifying root exudates. Additionally, *Mesorhizobium* is known for its role in N_2_-fixation through close symbiosis with plant roots. This association has been reported in the Canadian Arctic, for example, with *Oxytropis arctobia*^52^. Our results suggest that similar symbiotic processes may also occur in the Russian High-Arctic islands, for example, in lichens such as *Peltigera* or *Stereocaulon*, which are capable of fixing nitrogen^53^.

### High-Arctic MAGs are rich in biosynthetic gene clusters and antibiotic resistance genes

In our second hypothesis, we predicted that the nutrient input from vegetation would lead to a higher abundance of genes involved in C and N metabolism on vegetated islands. However, our results did not show a clear distinction in the number of such genes across the different soil environments. Instead, our results suggest that the studied soil environments harbour MAGs with varying capacities for C and N metabolism, regardless of vegetation cover and soil depth. For instance, the presence of Rubisco form I, together with CO-oxidation genes (*coxLMS*), indicates that both vegetated and unvegetated soils harbour a permafrost-affected soil microbiome with a functional gene potential for autotrophic C assimilation and atmospheric trace gas scavenging—traits frequently observed in oligotrophic soils^54,55^. The detection of Rubisco form IV (also called the Rubisco-like protein; RLP), which does not directly catalyse CO₂ fixation but is involved in other metabolic processes, further suggests high metabolic versatility. The incomplete representation of denitrification pathways, coupled with sporadic detection of *narGH*, *nirK*, *norBC*, and *nrfH*, points to limited but potentially functionally redundant N metabolism, likely constrained by redox and substrate availability. This observation is consistent with previous reports that the denitrification potential in dry or C-limited soils is often incomplete or tightly regulated^56,57^. Sulphur metabolism genes, including *sat*, *dsrAB*, and *sox*, while overall scarce, were more diverse in vegetated islands. The presence of these genes could reflect niche partitioning and microscale anoxic conditions favouring sulphur cycling^58^. On the other hand, the presence of taxa such as *Thiobacillus*, *Rudaea*, and *Hydrogenophaga* in vegetated islands—phyla known for their versatile metabolic capabilities—support the idea that plant-associated microbiomes harbour microbial communities with enhanced functional complexity^59^, even in extreme environments. These results suggest that, although there were no strong differences among the four studied soil environments, vegetation might slightly affect the microbial composition by promoting taxa with more versatile metabolic capabilities and greater microbial complexity, including the ability to use sulphur as an energy source.

We also predicted that unvegetated soils would harbour the largest number of BGCs and ARGs, due to high microbial competition in nutrient-poor environments. We found that BGCs indicative of antimicrobial, cytotoxic, and inhibitor compounds—especially NRPs, polyketides, and RiPPs—were prevalent in unvegetated soils, which may reflect greater microbial competition or stress under C and nutrient limitation^60,61^. Additionally, the higher richness and gene diversity of ARGs in unvegetated soils may reflect both a higher microbial turnover and inter-species competition under resource-limited conditions^62–64^. For instance, the predominance of multidrug, glycopeptide, and tetracycline resistance genes is consistent with ARG profiles observed in pristine but microbially competitive environments, where horizontal gene transfer and ancient resistance mechanisms persist independently of anthropogenic influence^65,66^. The co-occurrence of high BGC and ARG contents, in particular indicator MAGs such as 585, 543, and 587, further supports the hypothesis that secondary metabolism and resistance traits are often genomically linked, possibly as part of ecological arms races in microbial communities^67^. Furthermore, we found that, in general, the richness and gene diversity of BGCs, with a dominant fraction remaining unclassified, illustrate the potential for undiscovered bioactive metabolites in these unexplored and remote High-Arctic islands^68,69^.

### Vegetation cover and soil depth change the soil virome composition

In our third hypothesis, we predicted that vegetation would lead to increased viral diversity. Although our results did not indicate significant differences associated with vegetation cover, we observed lower viral richness in surface soils and a decrease in viral diversity with increasing soil depth, contrary to our hypothesis and in accordance with findings from a recent study from alpine permafrost in the Qinghai-Tibet Plateau^70^. Unfortunately, we found limited information on the effects of vegetation cover on soil viral communities in the literature, as most studies of High-Arctic soils have tended to be focused on bacterial communities while overlooking viruses. However, there are previous studies demonstrating that vegetation can strongly influence microbial communities in Arctic soils^71^. These effects have been reported to be site-specific^72^ and to depend on soil moisture, amongst other variables^73,74^, with soil moisture also being a key factor. Specifically, we found that the abundances of certain viral phyla differed with vegetation cover. For example, Pisuviricota was more abundant on unvegetated islands. Pisuviricota comprises RNA viruses with rapid replication cycles that primarily infect eukaryotes. This finding is intriguing, given that our sequencing targeted DNA extracted from soil samples rather than RNA. One possible explanation is that RNA viruses can be indirectly detected through their proviral DNA intermediates or via integrated viral sequences within host genomes^75^, which can be captured during DNA-based metagenomic sequencing. Therefore, shifts in the eukaryotic community may indirectly drive changes in RNA viral populations^76^. Although we did not find significant differences associated with vegetation cover in the eukaryotic kingdom, we found a higher abundance on unvegetated islands compared with vegetated ones. This may help explain the increased abundance of Pisuviricota on unvegetated islands. Also, Nucleocytoviricota is a phylum that includes the giant viruses, which are characterised by their large genome sizes and complex replication mechanisms^77^. These viruses infect a wide range of eukaryotic hosts, including algae and some metazoans. Previous research has suggested that giant viruses play crucial ecological roles by influencing microbial community dynamics, nutrient cycling, and even C fluxes through the lysis of host cells^78^. Within this phylum, the Mimiviridae family was notably abundant across all islands, highlighting the substantial role that giant viruses play in the High-Arctic soils. We also identified several bacteriophages, such as Demerecviridae and Tectiviridae, which had higher abundances in vegetated surface soils. This finding coincides with a significantly higher bacterial abundance, suggesting that the higher bacterial abundance provides a larger pool of hosts for these bacteriophages^79^. Both families, composed of double-stranded DNA viruses, infect diverse bacterial hosts and modulate microbial community structure through lytic infection cycles that cause bacterial cell lysis. This dynamic interaction likely drives nutrient recycling and shapes microbial diversity, emphasizing the tight coupling between phage abundance and bacterial communities^80^. Thus, the elevated presence of Demerecviridae and Tectiviridae in vegetated soils reflects not only their ecological role as regulators of bacterial populations but also the influence of vegetation in promoting favourable conditions for both bacteria and their viral predators within the soil ecosystems on High-Arctic islands.

This study provides the first comprehensive overview of the soil metagenomes from four Russian Arctic islands, comparing surface and deeper (cryoturbated permafrost) soils from vegetated and unvegetated islands. We found that vegetation cover affects the functional gene potential and virome, especially at greater soil depths, likely due to increased C and nutrient availability resulting from vegetation presence. Vegetation cover and sparse root growth in deeper soils can alter the functional gene potential, increasing the abundance of genes involved in C and N cycling and C catabolism, but also energy production, transcription, carbohydrate metabolism, and transport, thereby influencing the overall microbial functionality in permafrost-affected soils. MAGs revealed that, although there were no significant differences in the number of genes involved in C and N cycling across the different soil environments, there was a shift in microbial taxa. We also found a higher gene count and gene diversity of BGCs in unvegetated soils, suggesting stronger microbial competition. Furthermore, these soils harboured a higher abundance of ARGs, including those conferring resistance to fluoroquinolones, glycopeptides, and aminoglycosides. Together, these findings highlight a strong microbial competitive dynamic in these oligotrophic soil environments. Regarding the virome, both vegetation cover and soil depth significantly affected its diversity, which was highest in vegetated surface soils. Specifically, these soils had a higher abundance of Nucleocytoviricota (which includes giant viruses) and phages, such as Demerecviridae and Tectiviridae.

## Methods

### Study sites and soil sampling

The Arctic Century Expedition (ACE) of 2021 was a collaborative scientific expedition conducted aboard the Akademik Tryoshnikov icebreaker^30^. This ambitious undertaking was jointly organized by three prominent research institutions: the Swiss Polar Institute (SPI), Russia’s Arctic and Antarctic Research Institute (AARI), and Germany’s Helmholtz Centre for Ocean Research Kiel (GEOMAR). The expedition commenced on 5 August 2021, departing from Murmansk, Russia. Over the course of approximately 1 month, until 6 September 2021, the research team traversed the Barents, Kara, and Laptev Seas, as well as the open waters of the Arctic Ocean. The scientific team explored several remote Arctic islands, seven of which belong to the Franz-Josef Land and Severnaya Zemlya archipelagos and are located far from the major landmasses. Information about the biomass on Eurasian polar desert islands can be found in ref. ^30^. During the field work at each site, vegetation, soil and environmental variables were described in plots of 10 m × 10 m.

Soil samples were collected from four different islands: Graham Bell (G), Komsomolets (K), October Revolution (O), and Vize (V). Sampling locations were determined by the logistical constraints of the ACE expedition, including coordination with researchers from other disciplines and weather conditions. These constraints allowed only a short period (up to 6 h) of fieldwork on these specific islands and precluded access to other potential sites. The studied islands exhibit diverse landscapes and climatic conditions, due to their location on the north-eastern edge of the warm Atlantic water influence (Fig. 1). October Revolution and Vize are sparsely vegetated, while Graham Bell and Komsomolets are almost completely barren^30^. Located at 81° north in the Russian High-Arctic, Graham Bell is the easternmost island in the northernmost land of Eurasia (Fig. 1A, and Supplementary Fig. 1). The southern and western regions of this island are covered by the Kupol Vetrenny glacier, while the ice-free area is formed by sandstone. The study area is covered with dark-coloured rocky debris mixed with lighter sandy soil with abundant fragments of ancient coal. The island is almost completely non-vegetated, apart from rare patches of crust lichens, algae and a few mosses^30^. However, its soils can support at least one species of Enchytraeidae, which was found during the expedition^81^. Komsomolets, the northernmost island of Severnaya Zemlya (Fig. 1C, and Supplementary Fig. 2), has the most extreme climate conditions in the archipelago. It is largely covered by the Academy of Science glacier and has rarely been visited before. The study site in the northwestern part of the island is a vast sandy fluvio-glacial plain devoid of vegetation, with numerous scattered stones rarely covered by lichen crust patches. October Revolution, the largest island in the Severnaya Zemlya archipelago (Fig. 1D, and Supplementary Fig. 3), features five major glaciers covering about half of its surface. Non-glaciated areas have broad river valleys and lowlands. The study area was inland in a major river valley with red, iron-rich loamy soils to the south of the Rusanov Glacier. Vegetation is sparse (<10% of the cover on average) and is mostly represented by cryptogams with few vascular plant species present. Vize is a non-glaciated island, situated in the northern part of the Kara Sea between Franz Josef Land and Severnaya Zemlya archipelagos (Fig. 1B, and Supplementary Fig. 4). A distinct feature of the study area is ice wedge polygons that occur in the presence of permafrost. The soils are loamy and heavily intermixed with angular stones. Vize is the only island among the visited sites that has a predominantly continuous vegetation cover composed of algae crusts, mosses, lichens and forbs with very few graminoids present^30^.

Sampling took place during the ACE in August 2021. At the study site, we established three 1 m^2^ soil pits, each separated by a distance of 50 m. Excavations reached a maximum depth of 50 cm, with concurrent temperature monitoring along the soil profile. The presence of underlying permafrost was confirmed at 50 cm depth, with soil temperatures below 1 °C on Vize and October Revolution islands and below 0.5 °C on Graham Bell and Komsomolets islands. Morphologically, the soil profiles exhibited biological soil crust at the surface alongside evidence of cryoturbation in the deeper layers. Following the sampling design outlined by Sannino et al. (2022)^5^, we collected five subsamples of bulk soil (≥10 g per sample) from two distinct depth intervals: 0–2 cm and 30–50 cm. To access the top layer (0–2 cm) within the 1 m^2^ pit area, we utilised a soil core (5 cm diameter; 10 cm depth), after which the pits were manually excavated to reach the deeper layer. Subsamples corresponding to the same depth and pit were subsequently pooled, cleared of any visible roots, and completely homogenized in autoclaved bags. In the laboratory, the homogenised soil subsamples were sieved (4 mm mesh size) and stored at 4 °C for the remainder of the expedition, although a portion of each soil sample was dried immediately onsite. Samples designated for physico-chemical analyses were securely transported to the AARI, Saint Petersburg, for further analyses.

### Soil physico-chemical properties, greenhouse gas fluxes, botanical and climatic data

Soil physico-chemical characterisations were conducted in accordance with previously validated protocols (Perez-Mon et al., Frey et al., Frey et al., Frossard et al.)^24,82–84^. Soil pH was measured using a 1:2 (w/v) soil-to-water suspension. Gravimetric water content was assessed by oven-drying fresh soil aliquots at 105 °C for 24 h. Soil organic matter was quantified via loss-on-ignition by combusting the samples at 450 °C for 4 h. To determine total carbon (TC) and total nitrogen (TN) concentrations, samples were previously dried at 65 °C, finely milled and analysed using an elemental analyser (NC-2500; CE Instruments, Wigan, UK). Vegetation, including plant functional type–level cover estimates and species identifications (both cryptogams and vascular plants), was recorded using full Braun–Blanquet plots following the Arctic Vegetation Archive protocol^85^. High-resolution (0.5°) climatic variables were obtained from the WorldClim global climate database (v2.1; https://www.worldclim.org/). Information about the climatic and vegetation composition can be found in Supplementary Table 1. In situ greenhouse gas (GHG) fluxes were quantified following the methodology described by Hartmann et al.^86^. An experimental respiration chamber (15.6 L volume; 25 × 25 × 25 cm) was deployed on the soil surface under both light and dark conditions. Gas samples (20 mL) were extracted at 5, 20, 35 and 50-min intervals and immediately transferred to evacuated Exetainers vials. Concentrations of CO_2_, CH_4_ and N_2_O were determined using an Agilent 7890 gas chromatograph equipped with a flame ionization detector (Agilent Technologies, Palo Alto, CA, USA), calibrated concurrently with certified standard gas mixtures. Flux rates were mathematically derived through gas concentration-time regression models using the kappa.max method (gasfluxes() function) in *gasfluxes* R package^87^, factoring in ambient temperature and barometric pressure at the time of sampling. In this study, positive flux values denote net soil emissions, whereas negative values indicate net atmospheric consumption.

### DNA extraction and metagenomic sequencing

Metagenomic DNA was directly extracted onboard the research vessel using 5–10 g of fresh soil per sample using the DNeasy PowerMax Soil Kit (Qiagen, Hilden, Germany). To prevent cross-contamination, all laboratory surfaces and instruments were rigorously decontaminated with 5% sodium hypochlorite solution and subsequently wiped with 70% ethanol prior to processing. Extraction blanks (containing only reagents with no soil input) were processed in parallel to serve as negative controls. Following the extraction, the DNA eluates were transported in cold packs to the WSL laboratories in Switzerland. Upon arrival, DNA concentrations were determined via fluorometry using the PicoGreen assay (Invitrogen, Carlsbad, CA, USA), and the samples were stored at −20 °C. To confirm the integrity of the extraction process and rule out contamination, the negative controls were screened by PCR targeting the 16S rRNA gene. This was performed using the 341F (CCTAYGGGDBGCWSCAG) and 806R (GGACTACNVGGGTHTCTAAT) primer pair, as described by Frey et al.^88^. The absence of amplified products in these controls confirmed that there was no detectable contamination.

Bacterial and fungal microbial abundances were quantified through quantitative PCR (qPCR) using a 7500 Fast Real-Time PCR System (Thermo Fisher Scientific, Waltham, MA, USA). The protocols and specific primer sets employed for these quantifications were based on previously validated methods^24,83^. For this, 6.6 µL of DNA extract was added to each reaction. For bacterial quantification, the V1-V3 region of the 16S rRNA was amplified using the 27F/519R. For fungal, the internal transcribed spacer 2 (ITS2) region was targeted with the ITS3/ITS4 primer pair. To ensure absolute quantification, calibration curves were constructed from tenfold serial dilutions (ranging 10⁻¹ to 10⁹ gene copiesµL^-^^1^) of targeted-specific plasmids. All standard curves showed strong linearity (*R*² ≥ 0.99). The absence of amplified products in these controls confirmed that there was no detectable contamination.

Shotgun library preparation was carried out with a TrueSeq DNA Library Preparation Kit (Illumina, San Diego, CA, USA). In total, 24 samples (4 islands × 3 pits × 2 depths) were used for library preparation. Insufficient DNA for shotgun metagenomic sequencing was extracted from soil samples from Komsomolets at a soil depth of 30–50 cm. As a result, throughout the article, the soil depth on unvegetated islands is represented solely by soil samples collected from Graham Bell. Sequencing reactions were carried out on the Illumina NovaSeq platform (SP flow cell; 2 × 150 cycles) at Microsynth (Balgach, Switzerland). The raw sequences were deposited in the NCBI Sequence Read Archive under accession number PRJNA933553.

### Bioinformatics analyses of shotgun DNA metagenomic data

A customised pipeline was applied to carry out pre-processing of raw reads, assembly of pre-processed reads into contigs, and annotation of contigs for function and taxonomy^5,89,90^. Raw metagenomic paired-end (PE) reads were quality-checked using FastQC v0.11.9^91^, trimmed (sliding window of 4 base pairs [bp], average quality [Q] = 15 and minimum length = 40) using Trimmomatic v0.39^92^ and assembled into contigs (>200 bp) with MEGAHIT v1.2.9 (–k-min 27, –k-step 10)^93^. All samples were processed and assembled independently; no pooled assemblies were performed.

Gene-coding sequences (i.e. coding DNA sequences; CDSs) were predicted with MetaGeneMark v3.38^94^. The resulting predicted protein sequences were first assigned to general metabolic and cellular functions using the eggNOG v5 database, which classifies the genes to clusters of orthologous groups (COGs) of proteins and organizes the COGs into general functional categories^95^. Annotation to eggNOG was performed using eggNOG-mapper v2.1.6^96^ with the DIAMOND search function. PCGs were assigned to CAZymes using the CAZy database (July 2017 release^97^) and to N-cycling families using the NCyc database^98^, following the comparative metatranscriptomics workflow. Annotations for CAZymes and N-cycling families were done using SWORD v1.0.4^99^ with the same parameters (-v 10-5) as in Anwar et al.^100^. A manual categorisation of CAZy genes into different C substrates was performed according to Feng et al. and Varliero et al.^89,101^. PE read abundance per gene was quantified by mapping with BWA v0.7.17 (bwa-mem) and normalized by calculating counts per million (CPM). CPM is a normalization method used in metagenomics that represents the number of reads mapped to a particular gene, scaled by the total number of mapped reads in the sample, expressed per million reads. Throughout the article, we use the term “abundance” to refer to this normalization count. Quality-checked PE reads from each sample were mapped back to the assembled contigs, using BWA aligner v0.7.17 (bwa-mem)^102^. Raw counts of reads for the predicted CDSs were obtained using the featureCounts function from the *Subread* package v2.0.1 (-minOverlap 10, *Q* = 10, -primary^103^ and their CPMs. Taxonomic assignment was completed using Kaiju 1.10.0 (NCBI BLAST nr + euk database kaiju-addTaxonNames v2023.05.10^104^) and phyloFlash v3.4.2^105^ with the silva 138.1 reference database^106^. The number of raw reads and CDS can be found in Supplementary Tables 24 and 25. Viral sequences were identified using geNomad v3^107^ with default parameters. This tool employs a classification and annotation framework that integrates gene content analysis with a deep neural network to accurately detect and differentiate plasmid and viral sequences. For abundance estimation, we retained only viral sequences containing the designated marker genes and used their corresponding gene IDs to extract abundance values from the CPM table. Therefore, in this study, we are not analysing complete viral genomes, but rather viral gene-containing sequences (genetic fragments) recovered from the dataset.

MAGs were generated from the assembled contigs using Metabat v2.12.1^108^. Only contigs larger than 1500 bp with a read depth > 1 were included in the binning process. The resulting bins were assessed for quality using CheckM v1.1.3^109^. High-quality MAGs, defined as bins with > 50% completeness (COM) and < 10% contamination (CON), were selected for downstream analyses. DeepBGC^110^ and DeepARG^111^ were used with default parameters to predict BGCs and ARGs, respectively, in MAGs with > 87% completeness. DeepBGC utilises deep learning models, including a bidirectional long short-term memory recurrent neural network and a word2vec-like embedding approach, to predict BGCs in bacterial and fungal genomes. Similarly, DeepARG applies deep learning techniques to identify ARGs directly from metagenomic data. In this study, we only consider BGCs with a confidence score > 70% and ARGs with a probability score > 60% and identity score > 60%. Taxonomic classification of the high-quality MAGs was performed using GTDB-Tk v2.1.1^111,112^ against the GTDB database (release 202). Species novelty was assessed using average nucleotide identity (ANI) and alignment fraction (AF), with thresholds of < 95% ANI and > 60% AF^113^, calculated with skani v0.2.1^114^. The metabolic capabilities of these MAGs (> 87% completeness) were subsequently analysed using metabolisHMM v2.21^115^. To determine the abundance of each MAG across samples, reads from each sample were mapped to the bins using BWA. The coverage for each MAG was calculated as the total number of mapped bases divided by the MAG’s genome length. Relative abundance was then determined by normalizing each MAG’s coverage to the sum of the coverages of all MAGs within the sample. A summary of the performed analysis can be found in Supplementary Table 26.

### Statistics and reproducibility

All statistical analyses were performed in R v4.2.2^116^. Linear mixed-effects models (*lme4* package v1.1-32^117^) were used to evaluate the effects of vegetation cover and soil depth on the microbiome and virome on the four islands. Model assumptions were checked using Shapiro–Wilk tests for normality and visual inspection of residual plots for homoscedasticity. Model stability was assessed by parametric bootstrapping (100 simulations, bootMer function in *lme4*). If assumptions were not met, log-transformations were first applied to the response variable. If issues persisted, outliers (standardized residuals ±2 SD) were removed and the model was refitted to ensure robustness. Model results are presented as tables showing test statistics (*t*-values) and significance values (*P*-values) for vegetation cover (*v*), soil depth (*s*), and their interaction (*vs*), i.e. the fixed effects in the models. In these models, ‘island’ was included as a random effect to account for variation among islands and to control for the non-independence of samples from the same island. We analysed a total of *N* = 21 soil samples: *n* = 6 per treatment group (vegetated and unvegetated islands at 0–2 cm and 30–50 cm soil depth), with the exception of unvegetated soils at 30–50 cm depth (*n* = 3) due to failed DNA extraction in three samples. Throughout the article, positive or negative *t*-values indicate the direction of the effect. Specifically, a positive *t*-value for vegetation cover (*Tv*) indicates a positive association between vegetation cover and the response variable; therefore, we report that the value of the variable is higher on vegetated islands. Similarly, a positive *t*-value for soil depth (*Ts*) indicates that greater soil depth is positively associated with the response variable, and therefore, the value of the variable is higher at greater soil depth. A positive interaction effect (*Tvs*) indicates that the combined effect of vegetation cover and soil depth is positively associated with the variable in question, meaning that the effect of vegetation cover becomes stronger at greater soil depth. When one factor alone was analysed, non-parametric tests were used—either the Kruskal–Wallis or Mann–Whitney U test, depending on the number of groups being compared. To evaluate the influence of soil physico-chemical properties on functional gene potential, a Redundancy Analysis (RDA) was performed using the rda() function from the *vegan* package. The statistical significance was assessed via permutation tests using the anova.cca function with 999 permutations.

Microbial community composition (Kaiju-based taxonomy) at the phylum and genus level was analysed via non-metric multidimensional scaling (NMDS) to represent beta diversity, with statistical differences assessed through permutational multivariate analysis of variance (PERMANOVA) based on CPM data and Bray–Curtis dissimilarity at both taxonomic levels, implemented through *microeco* package v.0.16.0^118^. Microbial alpha-diversity was calculated using the Nearest Taxonomic Unit from phyloFlash. Functional alpha-diversity, i.e. richness and Shannon index, was calculated based on normalized data, using the *vegan* package v2.6-4^119^. Functional gene structure, which was evaluated using Bray–Curtis distances and displayed through canonical analysis of principal coordinates (CAP) and differences, was also tested using PERMANOVA using the adonis function from *vegan* package. Viral richness and Shannon diversity index were estimated using CPM-normalized data at the contig level, restricted to sequences classified as “viruses”. Linear discriminant analysis (LDA) effect size (LEfSe), implemented using the *lefser* package v1.16.2^120^ with default parameters, was employed to detect MAGs indicator taxa based on their relative abundances. The LEfSe output is presented as LDA scores, where the direction (positive or negative) indicates which soil environment had the higher MAG abundance.

### Reporting summary

Further information on research design is available in the Nature Portfolio Reporting Summary linked to this article.

## Supplementary information

Supplementary_material

Supplementary Data 1

Supplementary Data 2

Supplementary Data 3

Supplementary Data 4

Supplementary Data 5

Supplementary Data 6

Supplementary Data 7

Supplementary Data 8

Supplementary Data 9

Supplementary Data 10

Supplementary Data 11

Supplementary Data 12

Supplementary Data 13

Supplementary Data 14

Supplementary Data 15

Supplementary Data 16

Supplementary Data 17

Description of Additional Supplementary Files

Reporting Summary

Transparent Peer Review file

## Data Availability

The raw sequencing shotgun data were deposited in the NCBI Sequence Read Archive under BioProject accession number PRJNA933553. The source data for the figures in this paper are available in Supplementary Data 1–17. Any remaining information can be obtained from the corresponding author upon reasonable request.

## Code availability

The software used in this study includes Trimmomatic v0.39, MEGAHIT v1.2.9, MetaGeneMark v3.38, eggNOG-mapper v2.1.6, CAZy annotation scripts from https://github.com/anwarMZ/CoMW, Kaiju v1.10.0, deepARG v 1.0.2, and deepBCG v 0.1.29. The pipeline used in this manuscript is available on Zenodo at https://zenodo.org/records/19255448^121^.
