# Supplementary material for: Metagenomic insights into viral and microbial genes of Russian High-Arctic soil microbiomes
Source: Commun Biol. 2026 Apr 15;9:819. doi: 10.1038/s42003-026-10050-0 (PMC13269706; doi:10.1038/s42003-026-10050-0)
Supplement: Supplementary file 1 — Supplementary_material [file 42003_2026_10050_MOESM1_ESM.pdf]

Metagenomic insights into viral and microbial genes of Russian High-Arctic soil microbiomes

Beat Frey[1], Gilda Varliero[1], Joel Rüthi[1], Ivan Alekseev[2,3], Weihong Qi[4,5], Vasya Povazhnyi[6], Vitalii Zemlianskii[7], Beat Stierli[1], Ksenia Ermokhina[8], Gabriela Schaepman-Strub[7], Jessica Cuartero[1]

[1]Swiss Federal Institute for Forest, Snow and Landscape Research WSL, Birmensdorf, Switzerland
[2]State Scientific Center of the Russian Federation Arctic and Antarctic Research Institute, Saint Petersburg, Russia
[3]Karelian Research Centre of the RAS, Laboratory for Greenhouse Gas Monitoring, 185910, Petrozavodsk, Russia
[4]Functional Genomics Center Zurich, ETH Zurich and University of Zurich, Zurich, Switzerland
[5]Swiss Institute of Bioinformatics SIB, Geneva, Switzerland
[6]South Scientific Centre of Russian Academy of Science, Rostov-on-Don, Russia
[7]UZH, Department of Evolutionary Biology and Environmental Studies, Zurich, Switzerland
[8]A.N. Severtsov Institute of Ecology and Evolution, Russian Academy of Sciences, Moscow, Russia

Corresponding author: Beat Frey; beat.frey@wsl.ch

Supplementary Material

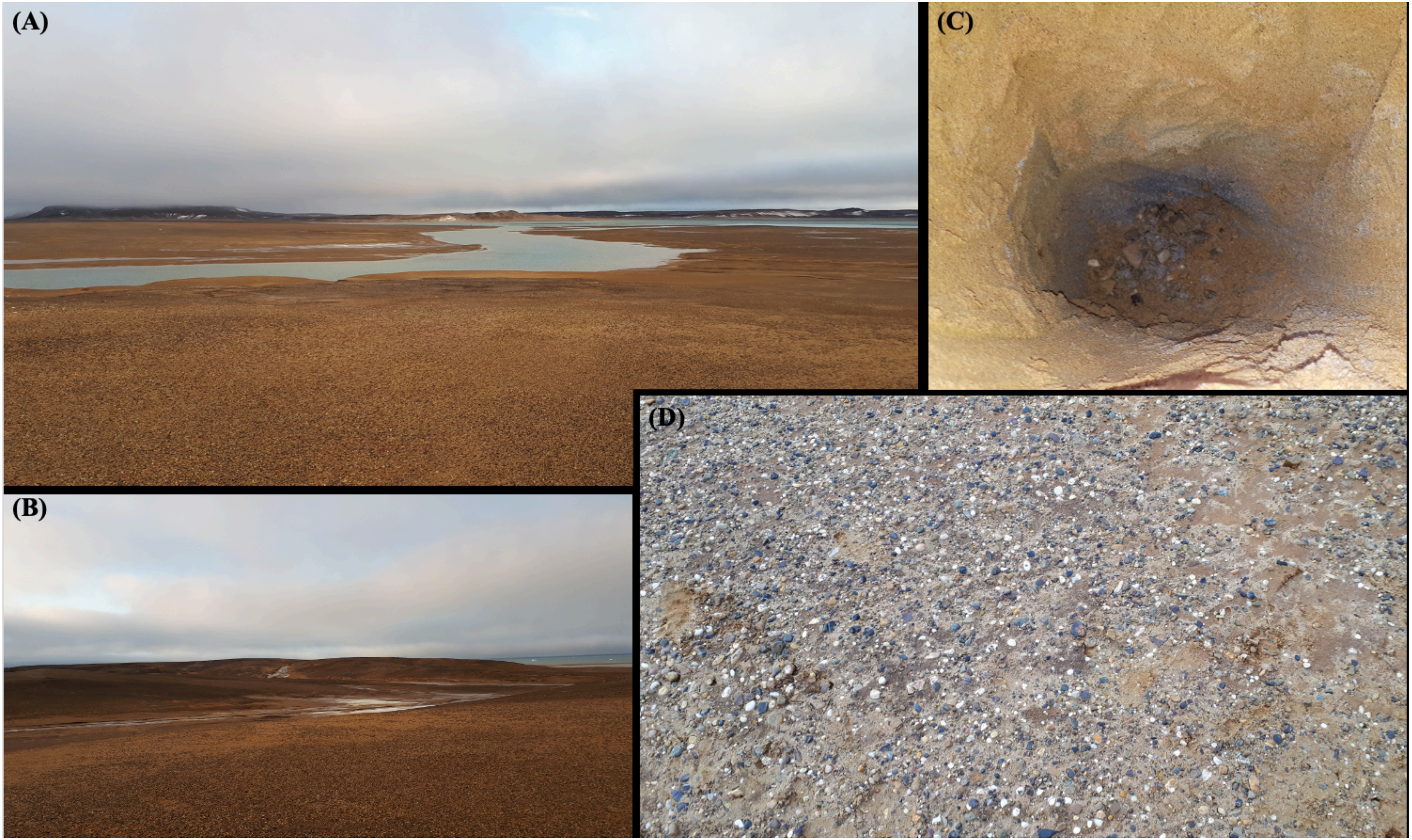

Supplementary Figure 1. Photographs of unvegetated Graham Bell (G) island: (A, B) landscape views; (C) soil profile; (D) soil.

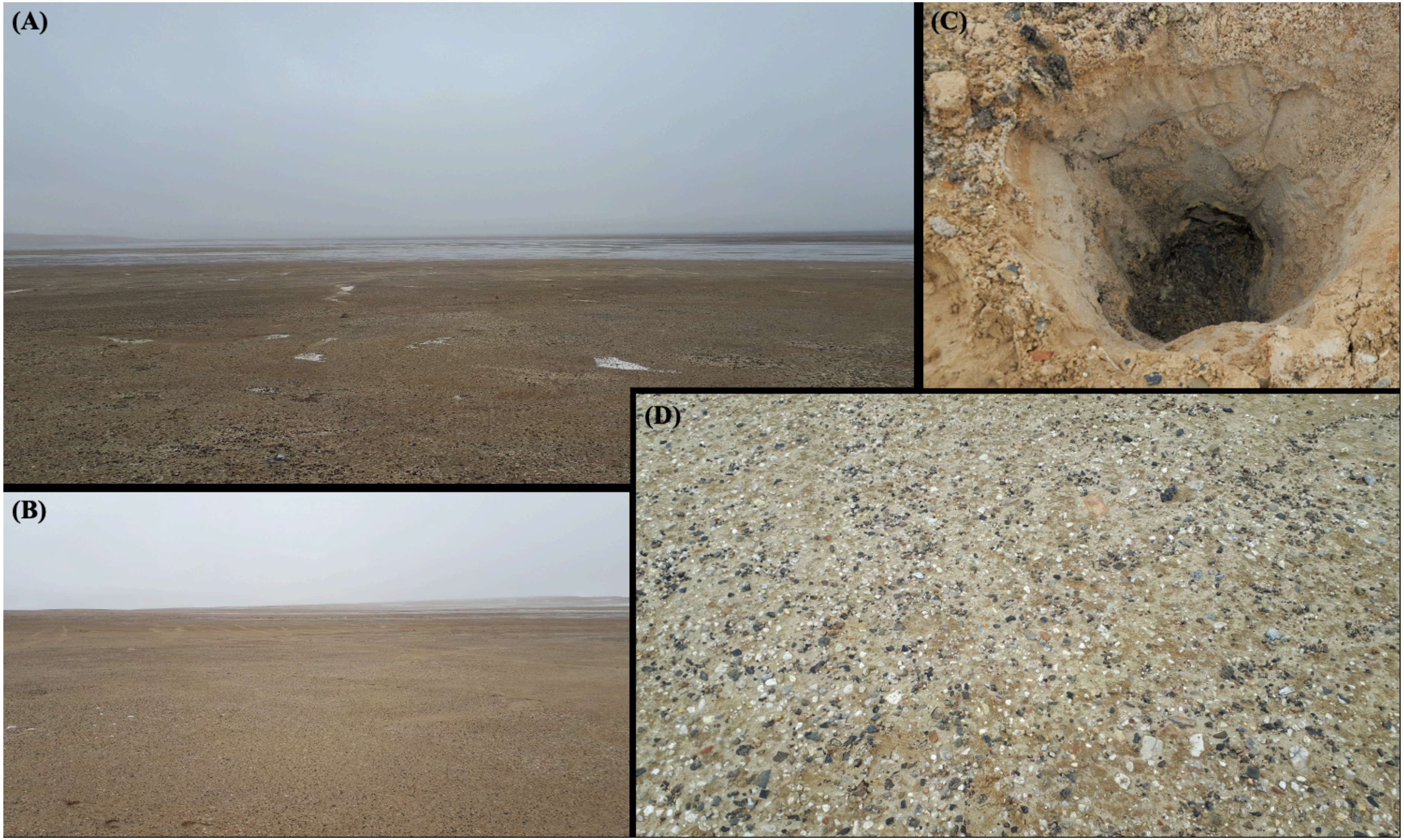

Supplementary Figure 2. Photographs of unvegetated Komsomolets (K) island: (A, B) landscape views; (C) soil profile; (D) soil.

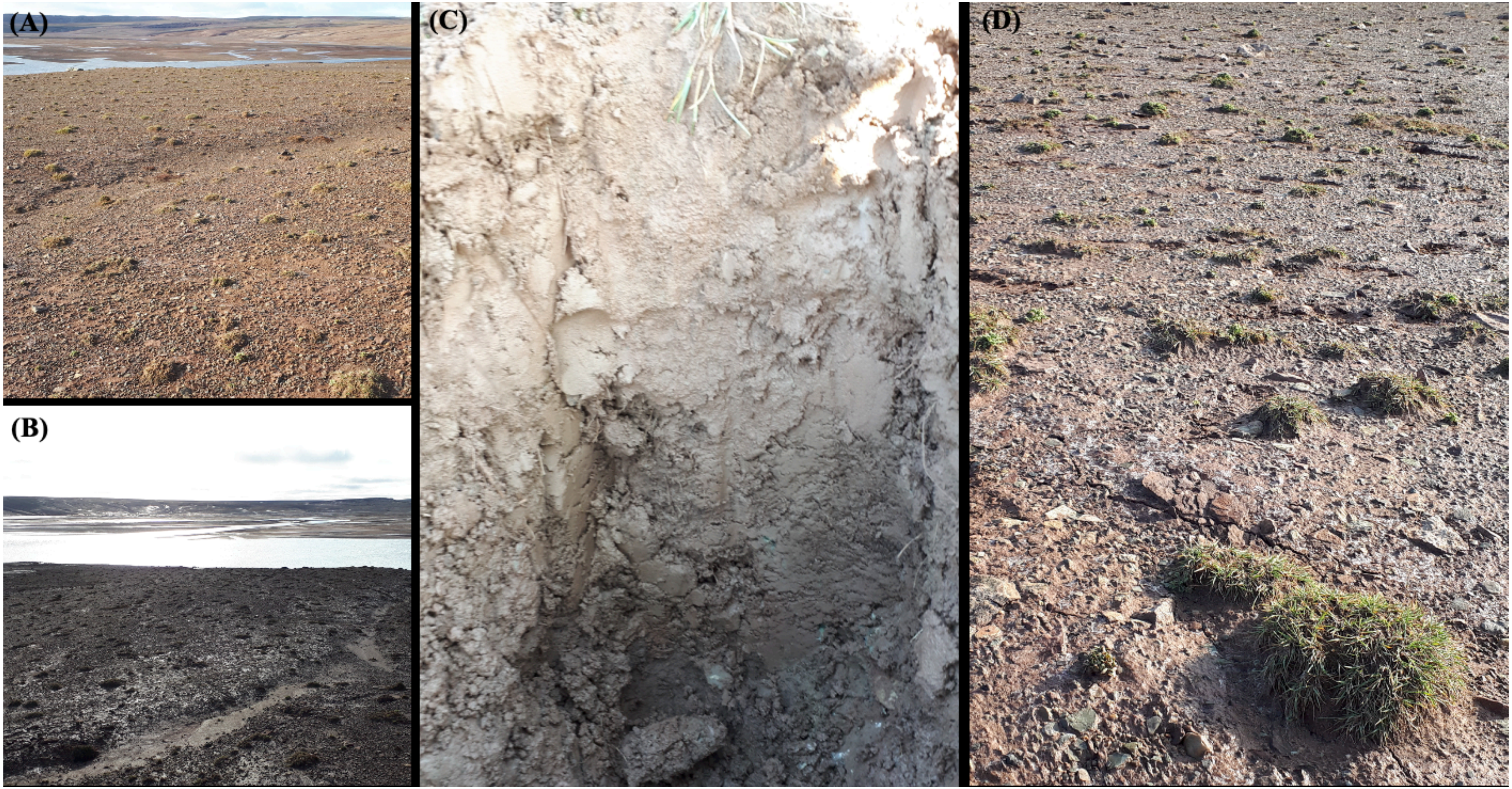

Supplementary Figure 3. Photographs of vegetated October Revolution (O) island: (A, B) landscape views; (C) soil profile; (D) soil.

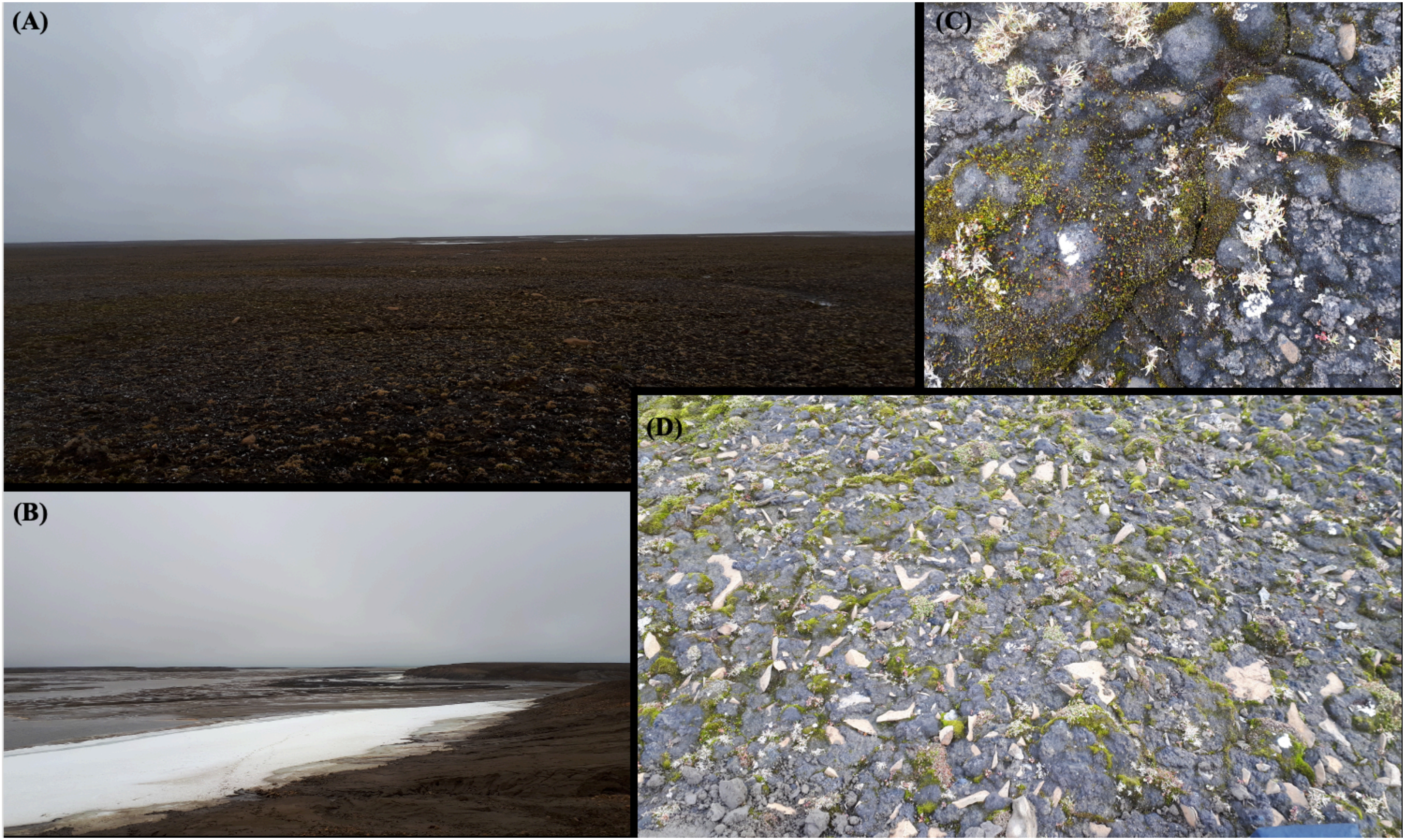

Supplementary Figure 4. Photographs of vegetated Vize (V) Island: (A, B) landscape views; (C, D) soil.

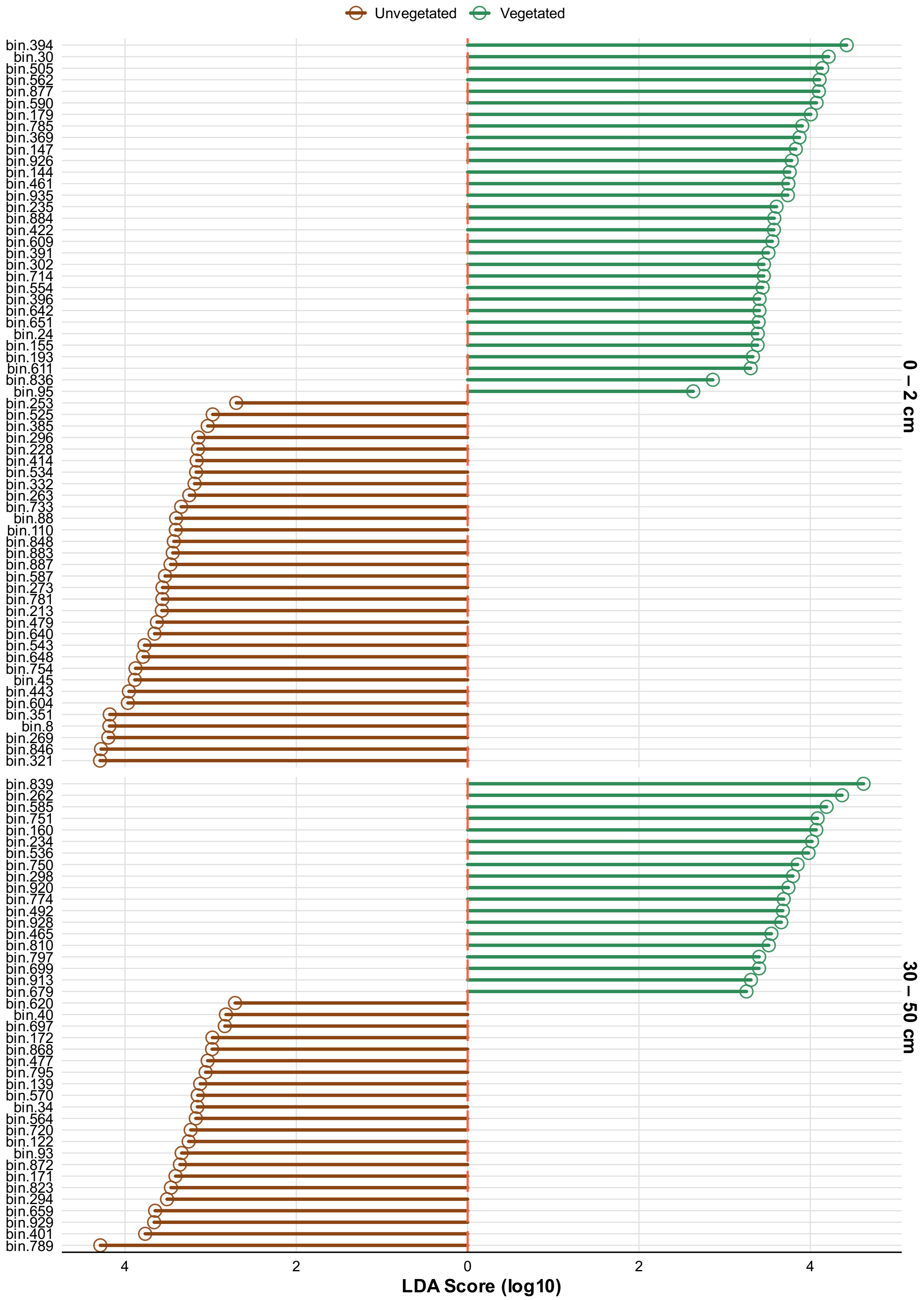

Supplementary Figure 5. Linear discriminant analysis (LDA) scores for metagenome-assembled genomes (MAGs) showing statistically significant differences ($P < 0.01$), based on vegetation cover and soil depth (N = 21). The direction of the scores indicates the soil environment in which the abundance is higher. Data are from shotgun metagenomic sequencing. Insufficient DNA for shotgun metagenomic sequencing was extracted from soil samples from Komsomolets at a depth of 30–50 cm, so soils at this depth from unvegetated islands are solely represented by soil samples collected from Graham Bell. Source data are provided in Supplementary Data 13.

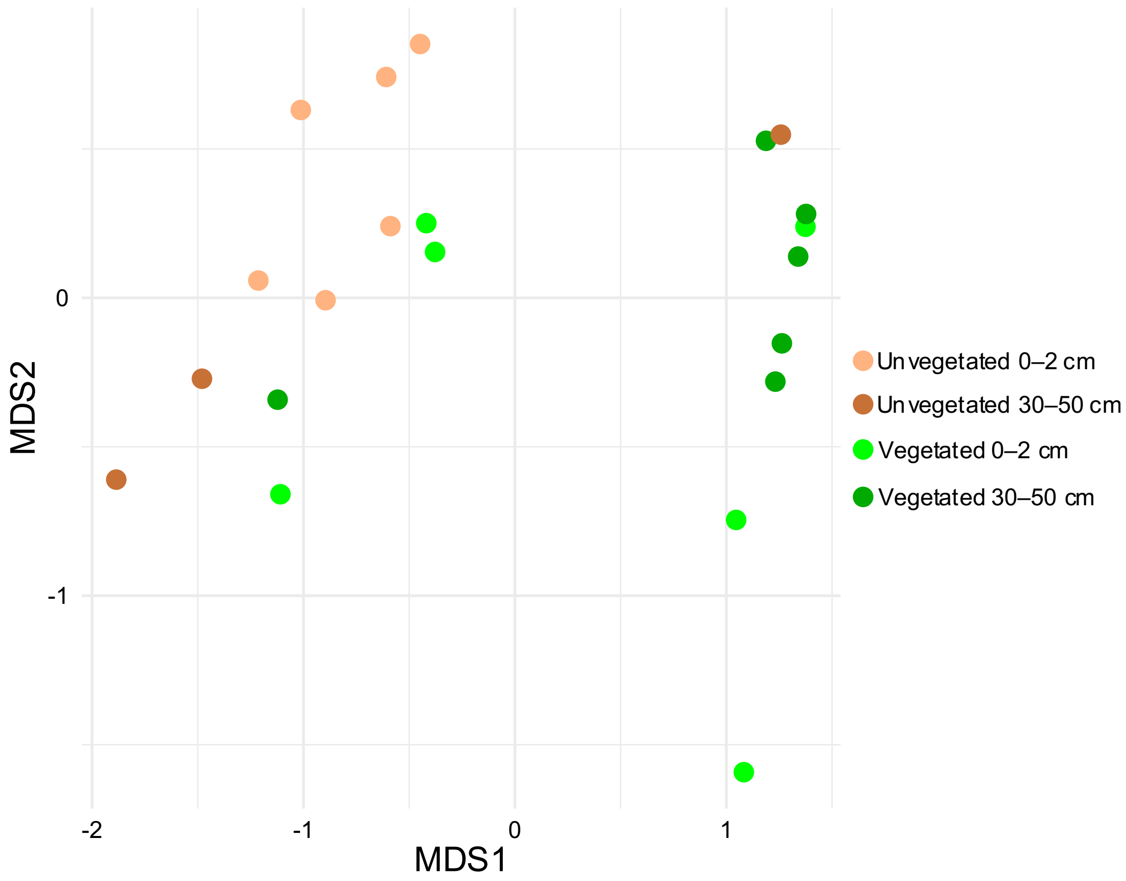

Supplementary Figure 6. Non-metric multidimensional scaling (NMDS) plots based on Bray–Curtis dissimilarity, showing virome structure (N = 21). Data are from shotgun metagenomic sequencing. Insufficient DNA for shotgun metagenomic sequencing was extracted from soil samples from Komsomolets at a depth of 30–50 cm, so soils at this depth from unvegetated islands are solely represented by soil samples collected from Graham Bell. Source data are provided in Supplementary Data 14.

Supplementary Table 1. Climatic information and vegetation cover of the High-Arctic islands Graham Bell (81°N, 65°E), Komsomolets (81°N, 94°E), October Revolution (79°N, 96°E), and Vize Island (79°N, 76°E), sampled in August 2021. The climatic information was obtained from the WorldClim database (0.5-degree resolution). Graham Bell (G) and Komsomolets (K) are unvegetated, while October Revolution (O) and Vize Island (V) are vegetated.

	G	K	O	V
Mean temperature (°C)	-14.9	-16.9	-16.6	-14.3
Annual precipitation (mm)	175	137	155	183
Minimum temperature (°C)	-30.1	-33.7	-34.0	-30.0
Maximum temperature (°C)	-0.2	0.6	1.8	1.9
Diurnal temperature range (°C)	4.5	4.7	4.9	4.8
Mean temperature of warmest quarter (°C)	-1.47	-1.82	-0.45	-0.42
Precipitation of warmest quarter (mm)	48	50	53	55
Number of plant species (n)	0	0	12	57
Vegetation cover (%)	0	0	6	30
Vascular plant cover (%)	0	0	5.5	13
Graminoid cover (%)	0	0	2	7
Forb cover (%)	0	0	3.5	6
Cryptogam cover (%)	0	0	0.5	17
Bryophyte cover (%)	0	0	0.5	12
Lichen cover (%)	0	0	0	5

Supplementary Table 2. Measurements of temperature and greenhouse gas emissions from soils. Values are presented as means ± standard deviation (n = 3). Statistical significance was assessed using the Mann-Whitney test (vegetation cover), with results reported as *P*-values. Unvegetated: Graham Bell (G), Komsomolets (K); Vegetated: October Revolution (O), Vize Island (V). Significant *P*-values are in bold.

	Air T (°C)	Soil T (°C)	CO_2 emission (μmol/m^2 day x10^3)	N_2O emission (μmol/m^2 day x10^3)	CH_4 emission (μmol/m^2 day x10^3)
G	2.1±0.2	1.4±0.2	-13.6±35.9	-768±2,770	3.3±10.3
K	0.4±0.0	1.1±0.0	0.15±1.11	-10,219±17,145	-6.7±23.1
O	0.9±0.6	0.8±0.6	-5.5±4.7	-1,790±9,693	-7.2±25.8
V	3.8±0.4	4.0±0.6	-0.1±0.3	-730±2.282	25.6±9.3
	$P = 0.17$	$P = 1.00$	$\boldsymbol{P = 0.002}$	$P = 0.39$	$P = 0.31$

Supplementary Table 3. Effects of vegetation cover and soil depth on taxonomic richness and Shannon index (n = 3). Indices were using phyloFlash and the reported results are in NTUs. Values are presented as means ± standard deviation. Statistical significance was assessed using linear mixed-effects models with vegetation (*v*), soil depth (*s*), and their interaction (*vs*) as fixed effects. *T-values* indicate effect direction and magnitude (positive/negative relationships). Unvegetated: Graham Bell (G), Komsomolets (K); Vegetated: October Revolution (O), Vize Island (V). Significant values are in bold. Data are from shotgun metagenomic sequencing. Insufficient DNA for shotgun metagenomic sequencing was extracted from soil samples from Komsomolets at a depth of 30–50 cm, so soils at this depth from unvegetated islands are solely represented by soil samples collected from Graham Bell.

	Richness	Shannon index
0–2 cm		
G	200±7	4.1±0.1
K	192±45	4.0±0.1
O	243±13	4.4±0.1
V	278±5	4.5±0.1
30–50 cm		
G	149±45	3.6±0.6
O	254±6	4.3±0.1
V	251±5	4.3±0.2
	$\mathbf{T_v = 4.95}$ $\mathbf{P_v = 0.003}$<br>$\mathbf{T_s = -3.30}$ $\mathbf{P_s = 0.004}$<br>$\mathbf{T_{vs} = 2.14}$ $\mathbf{P_{vs} = 0.048}$	$\mathbf{T_v = 4.29}$ $\mathbf{P_v = 0.035}$<br>$\mathbf{T_s = -5.32}$ $\mathbf{P_s < 0.001}$<br>$\mathbf{T_{vs} = 3.62}$ $\mathbf{P_{vs} = 0.003}$

Supplementary Table 4. Effects of vegetation cover and soil depth on the 15 most abundant phyla (counts per million, CPM) assigned by Kaiju. Values are presented as means ± standard deviation (n = 3). Statistical significance was assessed using linear mixed-effects models with vegetation (*v*) and soil depth (*s*) as fixed factors, their interaction (*vs*). *T*-values indicate effect direction and magnitude (positive/negative relationships). Unvegetated: Graham Bell (G), Komsomolets (K); Vegetated: October Revolution (O), Vize Island (V). Significant values are in bold. Data are from shotgun metagenomic sequencing. Insufficient DNA for shotgun metagenomic sequencing was extracted from soil samples from Komsomolets at a depth of 30–50 cm, so soils at this depth from unvegetated islands are solely represented by soil samples collected from Graham Bell.C: Candidatus genus. Source data are provided in Supplementary Data 15.

	Unclassified ($\times 10^3$)	Pseudomonadota ($\times 10^2$)	Actinomycetota ($\times 10^2$)	Acidobacteriota ($\times 10^2$)	Chloroflexota ($\times 10^2$)	Bacteroidota (×10)	Gemmatimonadota (×10)	Nitrospirota (×10)
<u>0–2 cm</u>								
G	2,793±82	1,913±510	1,794±201	840±103	1,299±478	858±197	1,496±416	135±7
K	2,559±176	2,662±256	1,610±157	1,161±193	432±60	1,074±315	841±544	120±17
O	2,753±163	2,576±216	1,434±128	848±169	366±176	3,953±1,019	4,486±336	763±63
V	2,948±166	2,570±125	1,125±146	756±25	391±60	4,996±355	1,750±245	698±28
<u>30–50 cm</u>								
G	1,906±274	2,532±745	3,353±1,303	709±170	585±250	440±177	501±364	152±43
O	2,481±202	2,668±515	1,002±92	1,318±189	365±74	4,700±1,172	4,276±1,233	1,141±287
V	2,596±406	3,050±1,487	1,393±632	818±197	534±241	3,151±787	3,678±1,296	580±36
	T_v = 1.00 P_v = 0.38<br>**T_s = -4.84 P_s < 0.001**<br>**T_{vs} = 2.32 P_{vs} = 0.033**	**T_v = -6.36 P_v < 0.001**<br>T_s = -0.40 P_s = 0.70<br>T_{vs} = 0.32 P_{vs} = 0.76	T_v = -1.09 P_v = 0.29<br>**T_s = 4.38 P_s < 0.001**<br>**T_{vs} = -3.56 P_{vs} = 0.002**	T_v = -0.96 P_v = 0.43<br>T_s = -1.16 P_s = 0.26<br>**T_{vs} = 2.44 P_{vs} = 0.027**	T_v = -1.20 P_v = 0.35<br>**T_s = -3.61 P_s = 0.002**<br>**T_{vs} = 3.24 P_{vs} = 0.005**	**T_v = -5.89 P_v < 0.001**<br>T_s = -0.39 P_s = 0.70<br>T_{vs} = 0.31 P_{vs} = 0.76	T_v = 1.90 P_v = 0.17<br>T_s = -1.33 P_s = 0.20<br>T_{vs} = 2.08 P_{vs} = 0.053	T_v = -0.96 P_v = 0.43<br>T_s = -1.16 P_s = 0.26<br>**T_{vs} = 2.44 P_{vs} = 0.027**

	Verrucomicrobiota (×10)	Planctomycetota (×10)	C. Eremiobacterota (×10)	Myxococcota (×10)	Cyanobacteriota (×10)	Bacillota	C. Dormibacteraeota	Thermomicrobiota
<u>0–2 cm</u>								
G	1,372±876	1,291±183	2,057±834	1,068±305	856±255	7,051±421	1,268±606	3,186±854
K	482±85	1,591±169	3,795±920	1,165±185	503±84	6,637±162	1,224±140	2,069±331
O	2,605±469	2,133±199	10.5±0.70	1,010±201	1,898±860	4,505±1,064	24.6±7.4	4,112±874
V	4,948±199	3,563±512	16.6±5.5	1,990±325	1,489±291	3,946±41	16.7±1.9	2,781±653
<u>30–50 cm</u>								
G	408±212	795±282	2,097±638	371±183	365±136	6,705±924	1,155±660	1,021±600
O	3,759±790	3,871±1,282	16.6±9.7	660±86	446±72	3,887±553	17.8±3.1	3,597±979
V	2,197±185	2,278±493	12.4±2.8	720±97	436±64	4,854±671	28.3±12.2	4,825±2,073
	**T_v = 4.81 P_v < 0.001**<br>T_s = -0.74 P_s = 0.47<br>T_{vs} = -0.31 P_{vs} = 0.76	**T_v = 2.74 P_v = 0.013**<br>T_s = -1.10 P_s = 0.29<br>T_{vs} = 1.15 P_{vs} = 0.26	T_v = -3.39 P_v = 0.07<br>T_s = -0.15 P_s = 0.88<br>T_{vs} = 0.13 P_{vs} = 0.90	T_v = 1.02 P_v = 0.38<br>**T_s = -3.86 P_s = 0.001**<br>T_{vs} = 0.21 P_{vs} = 0.84	**T_v = 4.53 P_v = 0.037**<br>**T_s = -3.36 P_s = 0.004**<br>**T_{vs} = -3.50 P_{vs} = 0.003**	**T_v = -6.99 P_v = 0.002**<br>T_s = -0.53 P_s = 0.60<br>T_{vs} = 1.32 P_{vs} = 0.20	**T_v = -6.92 P_v = 0.020**<br>**T_s = 2.35 P_s = 0.037**<br>T_{vs} = -2.09 P_{vs} = 0.058	T_v = 1.05 P_v = 0.31<br>T_s = -1.99 P_s = 0.06<br>**T_{vs} = 2.28 P_{vs} = 0.036**

Supplementary Table 5. Effects of vegetation cover and soil depth on the 15 most abundant genera (counts per million, CPM) assigned by Kaiju. Values are presented as means ± standard deviation (n = 3). Statistical significance was assessed using linear mixed-effects models with vegetation (*v*) and soil depth (*s*) as fixed factors, their interaction (*vs*). *T*-values indicate effect direction and magnitude (positive/negative relationships). Unvegetated: Graham Bell (G), Komsomolets (K); Vegetated: October Revolution (O), Vize Island (V). Significant values are in bold. Data are from shotgun metagenomic sequencing. Insufficient DNA for shotgun metagenomic sequencing was extracted from soil samples from Komsomolets at a depth of 30–50 cm, so soils at this depth from unvegetated islands are solely represented by soil samples collected from Graham Bell. Source data are provided in Supplementary Data 16.

	Bradyrhizobium (×10)	*Sphingomonas* (×10)	*Granulicella* (×10)	*Nocardioides* (×10)	*Streptomyces* (×10)	*Acidisphaera* (×10)	*Candidatus Dormibacter* (×10)	*Polaromonas* (×10)
0–2 cm								
G	2,006±786	606±84	862±287	267±16	696±80	614±318	1,071±647	55±14
K	2,497±210	815±114	4,256±712	289±59	565±56	2,208±173	1,083±144	70±16
O	905±66	2,811±1,041	28±3	1,404±693	414±18	27±7	12±2	488±232
V	1,195±292	1,309±186	47±3	583±111	424±47	38±5	7±1	165±37
30–50 cm								
G	3,186±2,968	295±144	1,391±1,094	601±149	1,149±374	626±370	1,008±657	55±37
O	1,276±338	2,033±751	432±686	721±266	281±57	24±12	7±2	260±6
V	948±123	741±339	31±3	781±219	367±137	22±5	11±4	1,796±2,343
	**$T_v = -5.02$ $P_v < 0.001$**	$T_v = 1.90$ $P_v = 0.19$	$T_v = 1.90$ $P_v = 0.19$	**$T_v = 2.86$ $P_v = 0.042$**	$T_v = -2.06$ $P_v = 0.06$	$T_v = -1.72$ $P_v = 0.23$	**$T_v = -4.80$ $P_v = 0.042$**	**$T_v = 4.36$ $P_v < 0.001$**
	$T_s = -0.50$ $P_s = 0.63$	$T_s = -1.05$ $P_s = 0.31$	$T_s = -1.05$ $P_s = 0.31$	$T_s = 1.26$ $P_s = 0.23$	$T_s = 4.88$ $P_s < 0.001$	$T_s = 1.73$ $P_s = 0.11$	$T_s = -0.62$ $P_s = 0.55$	$T_s = -0.09$ $P_s = 0.93$
	$T_{vs} = 0.56$ $P_{vs} = 0.58$	$T_{vs} = -1.60$ $P_{vs} = 0.13$	$T_{vs} = -1.60$ $P_{vs} = 0.13$	$T_{vs} = -1.73$ $P_{vs} = 0.10$	$T_{vs} = -4.47$ $P_{vs} < 0.001$	$T_{vs} = -1.53$ $P_{vs} = 0.15$	$T_{vs} = 0.53$ $P_{vs} = 0.61$	$T_{vs} = -0.32$ $P_{vs} = 0.75$

	Conexibacter	*Acidocella*	*Pseudonocardia*	*Mesorhizobium*	*Nitrospira*	*Thiobacillus* (×10)	*Edaphobacter* (×10)	Unclassified (×10^2)
0–2 cm								
G	9,825±1,943	53±24	8,433±2,801	2,156±497	474±66	6±2	434±151	6,594±232
K	7,060±1,506	653±413	5,514±573	2,589±104	435±39	9±2	1,1112±78	5,338±201
O	1,147±127	6±2	3,282±746	3,779±714	5,220±213	31±12	25±7	6,248±752
V	928±228	10±1	1,767±191	6,619±278	4,943±255	14±1	36±2	6,171±103
30–50 cm								
G	7,890±4,497	1,914±1,947	4,430±1,632	2,816±991	522±327	13±15	513±301	5,604±395
O	936±209	7±2	980±286	3,492±643	8,016±2,852	30±6	28±2	6,832±453
V	1,564±835	7±2	1,111±442	3,855±467	3,494±37	2,115±3,249	30±3	6,549±642
	**$T_v = -5.86$ $P_v = 0.028$**	$T_v = -0.92$ $P_v = 0.45$	$T_v = -3.08$ $P_v = 0.09$	$T_v = 3.17$ $P_v = 0.06$	**$T_v = 5.98$ $P_v = 0.016$**	$T_v = 0.17$ $P_v = 0.87$	$T_v = -2.16$ $P_v = 0.16$	$T_v = 0.30$ $P_v = 0.79$
	**$T_s = -4.14$ $P_s < 0.001$**	**$T_s = 9.03$ $P_s < 0.001$**	**$T_s = -3.50$ $P_s = 0.002$**	$T_s = 0.96$ $P_s = 0.35$	$T_s = 0.11$ $P_s = 0.92$	$T_s = 0.07$ $P_s = 0.95$	**$T_s = 4.26$ $P_s < 0.001$**	**$T_s = -2.50$ $P_s = 0.023$**
	**$T_{vs} = 3.65$ $P_{vs} = 0.002$**	**$T_{vs} = -8.07$ $P_{vs} < 0.001$**	$T_{vs} = 1.67$ $P_{vs} = 0.11$	**$T_{vs} = -2.74$ $P_{vs} = 0.014$**	$T_{vs} = -0.61$ $P_{vs} = 0.55$	$T_{vs} = 0.96$ $P_{vs} = 0.35$	**$T_{vs} = -3.06$ $P_{vs} = 0.002$**	**$T_{vs} = 3.13$ $P_{vs} = 0.007$**

Supplementary Table 6. Effects of soil properties on soil gene potential across the four studied High-Arctic (N = 21) islands using RDA. Multiple combinations were tested: the entire dataset, individual soil depths, and vegetated or unvegetated islands. *F* indicates F value and *P* denotes P-value, with statistically significant differences ($P < 0.05$) highlighted in bold.

	pH	SOC	Clay	Silt
Entire dataset	$F = 0.71$; $P = 0.4$	$F = 0.46$; $P = 0.8$	$F = 0.02$; $P = 0.9$	$F = 0.13$; $P = 0.8$
0–2 cm depth	**$F = 4.76$; $P = 0.021$**	**$F = 4.13$; $P = 0.023$**	$F = 0.46$; $P = 0.7$	$F = 2.39$; $P = 0.1$
30–50 cm depth	**$F = 9.21$; $P = 0.010$**	$F = 1.40$; $P = 0.4$	$F = 0.30$; $P = 0.7$	$F = 3.23$; $P = 0.1$
Unvegetated islands	$F = 4.52$; $P = 0.053$	$F = 9.26$; $P = 0.055$	$F = 0.30$; $P = 0.7$	$F = 0.44$; $P = 0.6$
Vegetated islands	$F = 4.09$; $P = 0.057$	$F = 0.13$; $P = 0.6$	$F = 0.40$; $P = 0.4$	$F = 1.84$; $P = 0.37$

Supplementary Table 7. Effects of vegetation cover and soil depth on abundances (counts per million, CPM) of super-categories assigned by the eggNOG database. Values are presented as means ± standard deviation (n = 3). Statistical significance was assessed using linear mixed-effects models with vegetation (*v*) and soil depth (*s*) as fixed factors, their interaction (*vs*). *T*-values indicate effect direction and magnitude (positive/negative relationships). Unvegetated: Graham Bell (G), Komsomolets (K); Vegetated: October Revolution (O), Vize Island (V). Significant values are in bold. Data are from shotgun metagenomic sequencing. Insufficient DNA for shotgun metagenomic sequencing was extracted from soil samples from Komsomolets at a depth of 30–50 cm, so soils at this depth from unvegetated islands are solely represented by soil samples collected from Graham Bell.

	Cellular processes and signalling (CPS) ($\times 10^{-2}$)	Information processing and storage (ISP) ($\times 10^{-2}$)	Metabolism (MBM) ($\times 10^{-2}$)
<u>0–2 cm</u>			
G	433±74	448±68	856±172
K	726±70	657±80	1,406±133
O	243±60	202±48	438±100
V	208±50	164±41	392±92
<u>30–50 cm</u>			
G	257±152	313±128	545±291
O	521±50	383±45	921±83
V	371±31	306±29	712±69
	T_v = -2.43 P_v = 0.13	T_v = -3.56 P_v = 0.07	T_v = -2.66 P_v = 0.11
	**T_s = -2.95 P_s = 0.009**	**T_s = -2.71 P_s = 0.015**	**T_s = -2.77 P_s = 0.013**
	**T_{vs} = 5.17 P_{vs} = 0.001**	**T_{vs} = 4.60 P_{vs} < 0.001**	**T_{vs} = 4.92 P_{vs} < 0.001**

Supplementary Table 8. Effects of vegetation cover and soil depth on the abundances (counts per million, CPM) of classes belonging to the cellular processes and signalling (CPS) super-category assigned by the eggNOG database. Values are presented as means ± standard deviation (n = 3). Statistical significance was assessed using linear mixed-effects models with vegetation (*v*) and soil depth (*s*) as fixed factors, their interaction (*vs*). *T*-values indicate effect direction and magnitude (positive/negative relationships). Unvegetated: Graham Bell (G), Komsomolets (K); Vegetated: October Revolution (O), Vize Island (V). D: cell cycle control, cell division, and chromosome portioning; M: cell wall/membrane/envelope biogenesis; N: cell motility, O: post-translational modification, protein turnover, and chaperones; T: signal transduction mechanisms; U: intracellular trafficking, secretion, and vesicular transport; V: defence mechanisms; W: extracellular structures; Y: nuclear structure; Z: cytoskeleton. Significant values are in bold. Data are from shotgun metagenomic sequencing. Insufficient DNA for shotgun metagenomic sequencing was extracted from soil samples from Komsomolets at a depth of 30–50 cm, so soils at this depth from unvegetated islands are solely represented by soil samples collected from Graham Bell.

	D ($\times10^{-1}$)	M ($\times10^{-2}$)	N ($\times10^{-1}$)	O ($\times10^{-1}$)	T ($\times10^{-1}$)	U ($\times10^{-1}$)	V ($\times10^{-1}$)	W	Y ($\times10^{2}$)	Z
<u>0–2 cm</u>										
G	244±41	136±26	160±18	658±112	1,141±191	399±60	357±61	31±7	175±55	102±20
K	380±40	228±25	308±14	1,092±97	1,905±177	725±59	549±65	73±8	753±183	162±25
O	119±33	71±19	105±16	349±85	647±152	267±63	225±59	33±9	5±4	91±14
V	98±27	59±15	99±19	291±71	564±133	221±52	200±48	29±7	37±15	91±19
<u>30–50 cm</u>										
G	139±79	79±49	81±52	380±226	684±423	237±136	244±117	14±13	72±124	62±39
O	252±30	153±15	242±26	722±78	1,345±113	610±73	478±40	69±17	31±53	186±25
V	194±26	107±14	185±43	529±51	980±97	398±16	340±25	44±5	169±243	108±25
	T_v = -3.03 P_v = 0.09<br>**T_s = -3.34 P_s = 0.004**<br>**T_{vs} = 5.44 P_{vs} < 0.001**	T_v = -2.55P_v = 0.12<br>**T_s = -2.89 P_s = 0.011**<br>**T_{vs} = 4.85 P_{vs} < 0.001**	T_v = -1.87 P_v = 0.20<br>**T_s = -3.27 P_s = 0.005**<br>**T_{vs} = 6.23 P_{vs} < 0.001**	T_v = -2.57 P_v = 0.12<br>**T_s = -3.18 P_s = 0.006**<br>**T_{vs} = 5.24 P_{vs} < 0.001**	T_v = -2.42 P_v = 0.13<br>**T_s = -2.87 P_s = 0.011**<br>**T_{vs} = 4.98 P_{vs} < 0.001**	T_v = -1.92 P_v = 0.19<br>**T_s = -2.68 P_s = 0.017**<br>**T_{vs} = 5.44 P_{vs} < 0.001**	T_v = -2.44 P_v = 0.13<br>**T_s = -2.34 P_s = 0.033**<br>**T_{vs} = 4.85 P_{vs} < 0.001**	T_v = -1.05 P_v = 0.40<br>T_s = -2.10 P_s = 0.052<br>**T_{vs} = 4.07 P_{vs} < 0.001**	T_v = -1.55 P_v = 0.26<br>T_s = -1.19 P_s = 0.25<br>T_{vs} = 1.60 P_{vs} = 0.13	T_v = -1.25 P_v = 0.33<br>T_s = -1.99 P_s = 0.063<br>**T_{vs} = 3.55 P_{vs} = 0.003**

Supplementary Table 9. Effects of vegetation cover and soil depth on abundances (counts per million, CPM) of classes belonging to the information processing and storage (ISP) super-category assigned by the eggNOG database. Values are presented as means ± standard deviation (n = 3). Statistical significance was assessed using linear mixed-effects models with vegetation (*v*) and soil depth (*s*) as fixed factors, their interaction (*vs*). *T*-values indicate effect direction and magnitude (positive/negative relationships). Unvegetated: Graham Bell (G), Komsomolets (K); Vegetated: October Revolution (O), Vize Island (V). A: RNA processing and modification; B: chromatin structure and dynamics; J: translation, ribosomal structure, and biogenesis; K: transcription; L: replication, recombination, and repair. Significant values are in bold. Data are from shotgun metagenomic sequencing. Insufficient DNA for shotgun metagenomic sequencing was extracted from soil samples from Komsomolets at a depth of 30–50 cm, so soils at this depth from unvegetated islands are solely represented by soil samples collected from Graham Bell.

	A	B	J ($\times10^{-2}$)	K ($\times10^{-2}$)	L ($\times10^{-2}$)
<u>0–2 cm</u>					
G	80±8	108±19	74±14	120±20	252±36
K	161±25	171±34	124±12	178±20	352±53
O	30±9	50±14	40±9	53±14	108±26
V	33±4	45±14	32±8	46±12	85±21
<u>30–50 cm</u>					
G	35±32	48±37	45±24	66±40	202±64
O	55±8	97±14	82±7	104±13	195±27
V	98±72	101±10	64±4	89±10	151±16
	$T_v = -2.23$ $P_v = 0.15$	$T_v = -3.14 P_v = 0.09$	$T_v = -2.58$ $P_v = 0.12$	$T_v = -3.59$ $P_v = 0.06$	$T_v = -4.01$ $P_v = 0.05$
	$T_s = -2.08$ $P_s = 0.054$	$\mathbf{T_s = -3.74}$ $\mathbf{P_s = 0.002}$	$\mathbf{T_s = -3.16}$ $\mathbf{P_s = 0.006}$	$\mathbf{T_s = -3.67}$ $\mathbf{P_s = 0.002}$	$T_s = -1.96$ $P_s = 0.07$
	$\mathbf{T_{vs} = 3.11}$ $\mathbf{P_{vs} = 0.007}$	$\mathbf{T_{vs} = 5.43}$ $\mathbf{P_{vs} < 0.001}$	$\mathbf{T_{vs} = 5.60}$ $\mathbf{P_{vs} < 0.001}$	$\mathbf{T_{vs} = 5.38}$ $\mathbf{P_{vs} < 0.001}$	$\mathbf{T_{vs} = 3.65}$ $\mathbf{P_{vs} = 0.002}$

Supplementary Table 10. Effects of vegetation cover and soil depth on abundances (counts per million, CPM) of classes belonging to the metabolism (MBM) super-category assigned by the eggNOG database. Values are presented as means ± standard deviation (n = 3). Statistical significance was assessed using linear mixed-effects models with vegetation (*v*) and soil depth (*s*) as fixed factors, their interaction (*vs*). *T*-values indicate effect direction and magnitude (positive/negative relationships). Unvegetated: Graham Bell (G), Komsomolets (K); Vegetated: October Revolution (O), Vize Island (V). C: energy production and conversion; E: amino acid transport and metabolism; F: nucleotide transport and metabolism; G: carbohydrate transport and metabolism; H: coenzyme transport and metabolism; I: lipid transport and metabolism; P: inorganic ion transport and metabolism; Q: secondary metabolites biosynthesis, transport, and catabolism. Significant values are in bold. Data are from shotgun metagenomic sequencing. Insufficient DNA for shotgun metagenomic sequencing was extracted from soil samples from Komsomolets at a depth of 30–50 cm, so soils at this depth from unvegetated islands are solely represented by soil samples collected from Graham Bell.

	C ($\times10^{-2}$)	E ($\times10^{-2}$)	F ($\times10^{-2}$)	G ($\times10^{-2}$)	H ($\times10^{-2}$)	I ($\times10^{-2}$)	P ($\times10^{-2}$)	Q ($\times10^{-1}$)
0–2 cm								
G	152±30	185±40	43±9	130±24	74±15	97±20	103±20	725±154
K	24±22	305±32	70±7	216±21	115±14	157±16	182±16	1,157±103
O	78±17	96±23	24±6	64±14	39±9	41±9	60±14	350±79
V	70±17	83±20	19±5	62±14	33±8	38±9	55±13	320±69
30–50 cm								
G	101±51	121±64	26±13	83±43	49±24	57±33	68±37	415±249
O	168±17	199±19	50±5	138±14	82±7	94±7	132±12	689±48
V	128±10	156±17	40±1	108±11	61±5	67±9	99±9	531±79
	T_v = -2.69 P_v = 0.11<br>**T_s = -2.58 P_s = 0.025**<br>**T_{vs} = 4.89 P_{vs} < 0.001**	T_v = -2.64 P_v = 0.12<br>**T_s = -2.58 P_s = 0.020**<br>**T_{vs} = 4.73 P_{vs} < 0.001**	T_v = -2.60 P_v = 0.12<br>**T_s = -3.05 P_s = 0.008**<br>**T_{vs} = 5.67 P_{vs} < 0.001**	T_v = -2.65 P_v = 0.12<br>**T_s = -2.81 P_s = 0.013**<br>**T_{vs} = 4.91 P_{vs} < 0.001**	T_v = -2.88 P_v = 0.10<br>**T_s = -2.63 P_s = 0.02**<br>**T_{vs} = 4.88 P_{vs} < 0.001**	T_v = -3.05 P_v = 0.09<br>**T_s = -3.25 P_s = 0.005**<br>**T_{vs} = 4.81 P_{vs} < 0.001**	T_v=-2.18 P_v=0.16<br>**T_s=-2.40 P_s=0.030**<br>**T_{vs}=4.92 P_{vs}<0.001**	T_v = -2.87 P_v = 0.09<br>**T_s = -3.20 P_s = 0.005**<br>**T_{vs} = 4.73 P_{vs} < 0.001**

Supplementary Table 11. Effects of vegetation cover and soil depth on abundances (counts per million, CPM) of (A) classes assigned by the CAZY database. Values are presented as means ± standard deviation (n = 3). Statistical significance was assessed using linear mixed-effects models with vegetation (*v*) and soil depth (*s*) as fixed factors, their interaction (*vs*). *T*-values indicate effect direction and magnitude (positive/negative relationships). Unvegetated: Graham Bell (G), Komsomolets (K); Vegetated: October Revolution (O), Vize Island (V). GT: glycosyl transferases; AA: auxiliary activities; CE: carbohydrate esterases; CBM: carbohydrate binding modules; GH: glycoside hydrolases; PL: polysaccharide lyases. Significant values are in bold. Data are from shotgun metagenomic sequencing. Insufficient DNA for shotgun metagenomic sequencing was extracted from soil samples from Komsomolets at a depth of 30–50 cm, so soils at this depth from unvegetated islands are solely represented by soil samples collected from Graham Bell.

	Anabolic processes	Catabolic processes				
	GT	AA	CE	CBM	GH	PL
<u>0–2 cm</u>						
G	3.777±597	343±62	672±139	1,219±179	3,385±520	54±13
K	5,743±742	594±68	997±125	1,983±243	5,618±642	76±20
O	2,102±521	197±47	329±57	1,166±262	1,884±398	59±8
V	1,759±452	174±44	301±60	965±235	1,648±373	61±8
<u>30–50 cm</u>						
G	2,303±1,280	247±113	428±234	771±404	2,285±1,009	38±27
O	4,071±364	324±36	790±54	2.285±299	3,835±365	139±18
V	3,115±246	307±63	481±83	1,456±182	2,684±409	78±13
	T_v = -2.87 P_v = 0.10<br>**T_s = -3.00 P_s = 0.008**<br>**T_{vs} = 4.95 P_{vs} < 0.001**	T_v = -2.34 P_v = 0.14<br>T_s = -2.07 P_s = 0.055<br>**T_{vs} = 3.72 P_{vs} = 0.002**	T_v = -2.96 P_v = 0.089<br>**T_s = -2.54 P_s = 0.022**<br>**T_{vs} = 4.47 P_{vs} < 0.001**	T_v = -1.27 P_v = 0.33<br>T_s = -2.12 P_s = 0.051<br>**T_{vs} = 4.48 P_{vs} < 0.001**	T_v = -2.44 P_v = 0.13<br>**T_s = -2.51 P_s = 0.024**<br>**T_{vs} = 4.56 P_{vs} < 0.001**	T_v = -0.37 P_v = 0.73<br>T_s = -1.22 P_s = 0.24<br>**T_{vs} = 3.32 P_{vs} = 0.004**

Supplementary Table 12. Effects of vegetation cover and soil depth on abundances (counts per million, CPM) of substrates assigned by the CAZY database. Values are presented as means ± standard deviation (n = 3). Statistical significance was assessed using linear mixed-effects models with vegetation (*v*) and soil depth (*s*) as fixed factors, their interaction (*vs*). *T*-values indicate effect direction and magnitude (positive/negative relationships). Unvegetated: Graham Bell (G), Komsomolets (K); Vegetated: October Revolution (O), Vize Island (V). Significant values are in bold. Data are from shotgun metagenomic sequencing. Insufficient DNA for shotgun metagenomic sequencing was extracted from soil samples from Komsomolets at a depth of 30–50 cm, so soils at this depth from unvegetated islands are solely represented by soil samples collected from Graham Bell.

	CAZy substrates								
	Cellulose	Chitin	Hemicellulose	Lignin	Multiple	Murein	Oligosaccharides	Pectin	Starch
0-2 cm									
G	399±72	740±99	310±49	342±62	602±105	190±29	1,144±199	352±63	742±113
K	625±104	1,179±167	492±48	594±68	991±196	287±52	1,912±220	635±73	1200±125
O	397±92	464±110	221±37	197±47	354±74	114±24	814±151	232±40	324±55
V	323±79	364±93	205±41	174±44	325±74	100±25	724±157	237±43	295±72
30-50 cm									
G	210±148	475±245	168±100	247±113	375±196	154±54	751±361	249±113	594±185
O	752±62	992±109	498±64	324±36	720±68	263±11	1,629±154	557±59	675±54
V	475±104	680±77	296±84	307±63	503±121	189±6	1,087±97	368±31	528±42
	T_v= -1.17 P_v= 0.35<br>**T_s= -2.48 P_s = 0.024**<br>**T_{vs}= 4.46 P_{vs} < 0.001**	T_v = -2.36 P_v = 0.14<br>**T_s = -2.53 P_s = 0.022**<br>**T_{vs} = 5.03 P_{vs} < 0.001**	T_v = -1.87 P_v = 0.19<br>**T_s = -2.61 P_s = 0.019**<br>**T_{vs} = 4.61 P_{vs} < 0.001**	T_v = -2.34 P_v = 0.14<br>T_s = -2.07 P_s = 0.056<br>**T_{vs} = 3.72 P_{vs} = 0.018**	T_v = -2.36 P_v = 0.14<br>**T_s = -2.67 P_s = 0.017**<br>**T_{vs} = 4.54 P_{vs} < 0.001**	T_v = -2.36 P_v = 0.14<br>**T_s = -2.67 P_s = 0.017**<br>**T_{vs} = 4.54 P_{vs} < 0.001**	T_v = -1.93 P_v = 0.19<br>**T_s = -2.38 P_s = 0.030**<br>**T_{vs} = 4.58 P_{vs} < 0.001**	T_v = -1.85 P_v = 0.20<br>T_s = -1.87 P_s = 0.08<br>**T_{vs} = 4.51 P_{vs}<0.001**	T_v = -2.93 P_v = 0.09<br>T_s = -1.94 P_s = 0.07<br>**T_{vs} = 4.41 P_{vs} < 0.001**

Supplementary Table 13. Effects of vegetation cover and soil depth on abundances (counts per million, CPM) of families assigned by the NCyc database. Values are presented as means ± standard deviation (n = 3). Statistical significance was assessed using linear mixed-effects models with vegetation (*v*) and soil depth (*s*) as fixed factors, their interaction (*vs*). *T*-values indicate effect direction and magnitude (positive/negative relationships). Unvegetated: Graham Bell (G), Komsomolets (K); Vegetated: October Revolution (O), Vize Island (V). ANR: assimilatory nitrate reduction; DDNR: denitrification and dissimilatory nitrate reduction; DNR: dissimilatory nitrate reduction; OD&S: organic degradation and synthesis. Significant values are in bold. Data are from shotgun metagenomic sequencing. Insufficient DNA for shotgun metagenomic sequencing was extracted from soil samples from Komsomolets at a depth of 30–50 cm, so soils at this depth from unvegetated islands are solely represented by soil samples collected from Graham Bell.

	Anammox ($\times10^2$)	ANR	Denitrification	DDNR	DNR	Nitrification ($\times10^2$)	Nitrogen fixation ($\times10^2$)	OD&S
0–2 cm								
G	211±171	122±22	26±7	11±5	70±9	147±115	972±267	1,003±166
K	1,000±152	183±23	37±4	15±2	109±16	136±43	2,550±615	1,477±180
O	34±19	70±14	56±22	21±6	29±3	412±43	34±21	623±141
V	49±19	63±14	51±14	21±5	29±5	387±65	161±71	518±124
30–50 cm								
G	102±156	84±40	24±17	42±10	51±16	123±98	228±395	798±313
O	44±44	138±10	150±9	87±20	72±12	1,211±303	105±93	1,165±100
V	63±25	101±20	127±17	81±7	73±40	488±225	103±85	924±51
	T_v = -1.44 P_v = 0.28<br>T_s = -1.41 P_s = 0.18<br>T_{vs} = 1.27 P_{vs} = 0.23	T_v = -2.78 P_v = 0.10<br>**T_s = -2.35 P_s = 0.031**<br>**T_{vs} = 4.30 P_{vs} < 0.001**	T_v = 1.73 P_v = 0.16<br>T_s = -0.47 P_s = 0.64<br>**T_{vs} = 6.70 P_{vs} < 0.001**	T_v = 1.33 P_v = 0.20<br>**T_s = 4.56 P_s < 0.001**<br>**T_{vs} = 4.20 P_{vs} < 0.001**	T_v = -3.37 P_v = 0.07<br>T_s = -1.83 P_s = 0.09<br>**T_{vs} = 3.99 P_{vs} = 0.001**	T_v = 1.09 P_v = 0.35<br>T_s = -0.13 P_s = 0.90<br>**T_{vs} = 2.24 P_{vs} = 0.039**	T_v = -2.16 P_v = 0.17<br>**T_s = -3.30 P_s = 0.004**<br>**T_{vs} = 2.72 P_{vs} = 0.016**	T_v = -2.78 P_v = 0.10<br>T_s = -1.79 P_s = 0.09<br>**T_{vs} = 4.29 P_{vs} < 0.001**

Supplementary Table 14. Functional gene abundance (CPMs) and carbohydrate-active enzyme (CAZyme) families detected in samples from O and V conditions. CPS: cellular processes and signalling; IPS: information processing and storage; MBM: metabolism; AA: auxiliary activities; CE: carbohydrate esterases; CBM: carbohydrate-binding modules; GH: glycoside hydrolases; PL: polysaccharide lyases; OD&S: other domains and signatures. Nitrogen-cycle genes are grouped as follows: Anammox, ANR (assimilatory nitrate reduction), Denitrification, DNR (dissimilatory nitrate reduction), Nitrification, and Nitrogen fixation.

	CPS ($\times 10^{-2}$)	ISP ($\times 10^{-2}$)	MBM ($\times 10^{-2}$)	AA	CE	CBM	GH	PL	Anammox	ANR	Denitrification	DNR	Nitrification	Nitrogen fixation	OD&S
O															
Sample 1	21	20	41	655	1,317	3,508	6,717	212	1	54	33	26	5	1	478
Sample 2	22	20	40	660	1,238	3,697	6,201	195	1	73	76	31	4	1	759
V															
Sample 1	22	19	42	561	1,281	3,274	6,695	337	1	57	41	26	4	2	434
Sample 2	22	19	42	511	1,063	2,753	5,565	257	1	53	45	26	3	1	460

Supplementary Table 15. Metagenome-assembled genome (MAGs) information from unvegetated islands at 0–2 cm soil depth, including MAG identification (bin number) genome completeness (COM), contamination (CON), taxonomic assignment (phylum and genus level), average nucleotide identity (ANI), and alignment fraction (AF) values expressed as percentages. Unvegetated islands: Graham Bell and Komsomolets (LEfSe $P < 0.01$). Novel species (defined as having <95% ANI and >60% AF) are highlighted in bold. Data are from shotgun metagenomic sequencing.

MAG	COM	CON	Taxa	ANI	AF	MAG	COM	CON	Taxa	ANI	AF
88	74.8	8.6	KBS-83 (Acidobacteriota)	NA	NA	351	57.5	8.9	JANYGA01 (Eremiobacterota)	NA	NA
45	77.4	6.9	*Granulicella* (Acidobacteriota)	NA	NA	321	82.5	4.9	JANYGA01 (Eremiobacterota)	98	76
213	52.8	8.6	*Granulicella* (Acidobacteriota)	84	21	263	55.7	3.4	JAMXLG01 (Eremiobacterota)	NA	NA
648	60.4	7.2	DOZZ01 (Acidobacteriota)	84	16	534	50.6	5.9	JALYVW01 (Eremiobacterota)	NA	NA
228	58.2	8.6	BOG-234 (Acidobacteriota)	NA	NA	296	87.2	0	JALYVW01 (Eremiobacterota)	NA	NA
**8**	**87.0**	**8.2**	***Rhodococcus* (Actinomycetota)**	**94**	**67**	846	64.3	9.8	JANYGA01 (Eremiobacterota)	NA	NA
332	86.3	3.7	JANXMY01 (Armatimonadota)	NA	NA	733	79.9	6.5	JALYVT01 (Eremiobacterota)	NA	NA
525	98.1	0.7	JACMLD01 (Bacteroidota)	NA	NA	273	63.8	4.8	Unclassified (Eremiobacterota)	NA	NA
253	82.3	0.9	JAMPXM01 (Bdellovibrionota)	NA	NA	479	96.3	5.4	*Rhodopila* (Pseudomonadota)	NA	NA
604	80.1	6.5	UBA6019 (Chloroflexota)	87.5	40	883	86.7	7.9	Palsa-881 (Pseudomonadota)	NA	NA
385	81.6	2.8	UBA5177 (Chloroflexota)	NA	NA	781	57.8	1.7	Palsa-881 (Pseudomonadota)	NA	NA
269	51.6	5.2	Palsa-875 (Chloroflexota)	NA	NA	848	77.7	2.6	CAILRJ01 (Pseudomonadota)	NA	NA
443	80.6	9.6	Unclassified (Chloroflexota)	NA	NA	543	72.5	5.2	CAHJXG01 (Pseudomonadota)	NA	NA
754	91.1	3.1	JANWJD01 (Chloroflexota)	NA	NA	887	92.6	3.9	Bog-1198 (Pseudomonadota)	NA	NA
640	65.1	5.0	Unclassified (Chloroflexota)	NA	NA	**587**	**88.6**	**3.6**	**Bog-1198 (Pseudomonadota)**	**91**	**67**
110	76.9	1.3	Unclassified (Chloroflexota)	NA	NA	414	79.3	2.3	Unclassified (Chloroflexota)	NA	NA

Supplementary Table 16. Metagenome-assembled genome (MAGs) information from unvegetated islands at 30–50 cm soil depth, including MAG identification (bin number) genome completeness (COM), contamination (CON), taxonomic assignment (phylum and genus level), average nucleotide identity (ANI), and alignment fraction (AF) values expressed as percentages. Unvegetated island: Graham Bell (LEfSe $P < 0.01$). Novel species (defined as having <95% ANI and >60% AF) are highlighted in bold. NA indicates that ANI and AF values could not be calculated due to insufficient genomic similarity or lack of a suitable reference genome. Data are from shotgun metagenomic sequencing. Insufficient DNA for shotgun metagenomic sequencing was extracted from soil samples from Komsomolets at a depth of 30–50 cm, so soils at this depth from unvegetated islands are solely represented by soil samples collected from Graham Bell.

MAG	COM	CON	Taxa	ANI	AF	MAG	COM	CON	Taxa	ANI	AF
823	63.1	1.7	JAKAAZ01 (Acidobacteriota)	NA	NA	122	94.1	4.0	Unclassified (Chloroflexota)	NA	NA
294	79.2	6.4	JAKAAZ01 (Acidobacteriota)	NA	NA	139	85.6	0.5	JACWAJ01 (Chloroflexota)	NA	NA
40	92.9	5.1	JAJZIJ0 (Acidobacteriota)	NA	NA	789	94.8	4.2	Bog-877 (Chloroflexota)	98	74
697	74.1	3.4	Unclassified (Acidobacteriota)	NA	NA	34	66.1	2.2	Bog-877 (Chloroflexota)	NA	NA
570	74.8	1.3	Unclassified (Actinomycetota)	NA	NA	564	88.6	1.9	*Velthaea* (Eremiobacterota)	NA	NA
171	86.4	8.1	RYN-46 (Actinomycetota)	NA	NA	**795**	**87.5**	**0.9**	**Eremiobacterota (Eremiobacterota)**	**90**	**72**
659	96.6	1.3	RAAP-2 (Actinomycetota)	NA	NA	720	96.3	5.2	DAHUXY01 (Eremiobacterota)	NA	NA
868	93.6	1.3	JAKBAA01 (Actinomycetota)	NA	NA	477	75.6	2.8	DAHUXY01 (Eremiobacterota)	NA	NA
93	61.4	0.0	Unclassified (Actinomycetota)	NA	NA	872	92.7	3.6	Unclassified (Eremiobacterota)	NA	NA
401	94.7	5.2	Bog-756 (Actinomycetota)	NA	NA	172	77.6	0.0	JAJYPN01 (Eremiobacterota)	NA	NA
620	88.4	0.0	Unclassified (Chloroflexota)	NA	NA	929	58.4	8.8	Unclassified (Chloroflexota)	NA	NA

Supplementary Table 17. Metagenome-assembled genome (MAGs) information from vegetated islands at 0–2 cm soil depth, including MAG identification (bin number) genome completeness (COM), contamination (CON), taxonomic assignment (phylum and genus level), average nucleotide identity (ANI), and alignment fraction (AF) values expressed as percentages. Vegetated islands: October Revolution and Vize Island (LEfSe $P < 0.01$). Novel species (defined as having <95% ANI and >60% AF) are highlighted in bold. NA indicates that ANI and AF values could not be calculated due to insufficient genomic similarity or lack of a suitable reference genome. Data are from shotgun metagenomic sequencing.

MAG	COM	CON	Taxa	ANI	AF	MAG	COM	CON	Taxa	ANI	AF
147	83.7	9.9	JAIVJZ01 (Acidobacteriota)	NA	NA	505	51.4	4.4	*Sphingomicrobium* (Pseudomonadota)	NA	NA
179	82.8	8.7	Gp7-AA6 (Acidobacteriota)	NA	NA	714	82.8	5.7	*Rudaea* (Pseudomonadota)	88	39
193	91.2	2.1	Unclassified (Actinomycetota)	NA	NA	422	78.3	2.2	Unclassified (Pseudomonadota)	NA	NA
590	69.0	8.6	JAENVT01(Actinomycetota)	NA	NA	785	90.4	5.6	*Qipengyuania* (Pseudomonadota)	82	18
884	89.7	2.5	JACDCJ01 (Actinomycetota)	95	65	391	50.9	1.7	*Povalibacter* (Pseudomonadota)	NA	NA
642	88.0	3.2	JACDBE01 (Actinomycetota)	97	78	30	54.9	2.7	*Polaromonas* (Pseudomonadota)	NA	NA
651	73.2	3.4	JACCTH01 (Actinomycetota)	NA	NA	611	63.8	0.0	PALSA-1196 (Pseudomonadota)	NA	NA
562	90.6	1.5	*Ilumatobacter* (Actinomycetota)	NA	NA	369	82.4	8.0	PALSA-1003 (Pseudomonadota)	81	21
836	88.7	0.5	Unclassified (Actinomycetota)	NA	NA	**394**	**78.3**	**8.6**	**LMDS01 (Pseudomonadota)**	**90**	**61**
95	97.0	1.1	Unclassified (Bacteroidota)	NA	NA	396	85.9	0.8	JACMNW01 (Pseudomonadota)	NA	NA
554	90.2	4.7	*Chryseolinea* (Bacteroidota)	NA	NA	926	73.6	1.7	JACCXJ01 (Pseudomonadota)	NA	NA
24	80.3	1.6	*Aquaticitalea* (Bacteroidota)	NA	NA	877	70.1	3.4	*Hydrogenophaga* (Pseudomonadota)	NA	NA
609	72.5	8.0	Unclassified (Chloroflexota)	NA	NA	935	85.7	5.0	*Dokdonella* (Pseudomonadota)	84	33
302	79.1	2.0	*Stenomitos* (Cyanobacteriota)	99	89	461	69.0	5.2	CADEED01 (Pseudomonadota)	NA	NA
144	67.5	2.3	JAICNR01 (Gemmatimonadota)	NA	NA	235	76.1	6.8	Unclassified (Verrucomicrobiota)	NA	NA
155	67.9	0.6	Unclassified (Myxococcota)	NA	NA						

Supplementary Table 18. Metagenome-assembled genome (MAGs) information from vegetated islands at 30–50 cm soil depth, including MAG identification (bin number) genome completeness (COM), contamination (CON), taxonomic assignment (phylum and genus level), average nucleotide identity (ANI), and alignment fraction (AF) values expressed as percentages. Vegetated islands: October Revolution and Vize Island (LEfSe $P < 0.01$). NA indicates that ANI and AF values could not be calculated due to insufficient genomic similarity or lack of a suitable reference genome. Data are from shotgun metagenomic sequencing.

MAG	COM	CON	Taxa	ANI	AF	MAG	COM	CON	Taxa	ANI	AF
492	93.7	9.8	OLB17 (Acidobacteriota)	NA	NA	585	82.3	4.1	*Pseudomonas*_E (Pseudomonadota)	NA	NA
920	60.3	5.2	Unclassified (Acidobacteriota)	NA	NA	679	96.5	3.9	*Phyllobacterium* (Pseudomonadota)	97	90
750	92.9	7.7	Unclassified (Acidobacteriota)	NA	NA	810	93.5	1.2	*Cypionkella* (Pseudomonadota)	NA	NA
298	85.0	5.3	CAMPKL01 (Acidobacteriota)	87	48	465	65.5	1.7	CAMLHT01 (Gemmatimonadota)	NA	NA
797	95.7	3.8	UBA3006 (Actinomycetota)	NA	NA	536	98.2	5.9	SXOA01 (Planctomycetota)	NA	NA
928	86.9	4.7	JACDBF01 (Actinomycetota)	87	38	234	56.9	5.0	JALHQU01 (Planctomycetota)	NA	NA
262	76.1	5.5	*Arthrobacter* (Actinomycetota)	87	38	751	70.0	5.1	UBA5216 (Pseudomonadota)	NA	NA
699	91.4	1.5	*Flavitalea* (Bacteroidota)	NA	NA	839	72.6	5.2	*Thiobacillus* (Pseudomonadota)	NA	NA
913	86.3	7.1	Unclassified (Bdellovibrionota)	NA	NA	160	96.2	7.6	*Thiobacillus* (Pseudomonadota)	NA	NA
774	62.3	2.8	Palsa-1033 (Chloroflexota)	98	89						

Supplementary Table 19. Richness (number of BGC-encoding genes) of biosynthetic gene clusters (BGCs) and BCG classes identified by the DeepBGC program across islands and at two different soil depths. Unvegetated: Graham Bell (G), Komsomolets (K); Vegetated: October Revolution (O), Vize Island (V). Non-ribosomal peptide (NRP); ribosomally synthesized and post-translationally modified peptides (RiPP). Data are from shotgun metagenomic sequencing. Insufficient DNA for shotgun metagenomic sequencing was extracted from soil samples from Komsomolets at a depth of 30–50 cm, so soils at this depth from unvegetated islands are solely represented by soil samples collected from Graham Bell.

	Unvegetated 0–2 cm	Unvegetated 30–50 cm	Vegetated 0–2 cm	Vegetated 30–50 cm
BCG				
antibacterial	720	443	516	355
antibacterial-antifungal	5	8	8	3
antibacterial-cytotoxic	0	0	0	1
antifungal	0	1	0	0
cytotoxic	32	24	14	12
inhibitor	25	11	15	6
unclassified	333	236	209	145
BCG classes				
NRP	13	10	6	21
NRP-polyketide	8	2	2	3
other	26	14	9	13
polyketide	132	109	122	62
polyketide-terpene	9	4	2	2
RiPP	61	53	59	50
saccharide	166	103	85	59
saccharide-terpene	0	0	1	
terpene	26	18	23	11
unclassified	674	410	453	301
	1115	723	762	522

Supplementary Table 20. Richness (number of ARG-encoding genes) of antimicrobial resistance gene (ARG) compounds identified by the DeepARG program across islands and at two different soil depths. Unvegetated: Graham Bell (G), Komsomolets (K); Vegetated: October Revolution (O), Vize Island (V). Data are from shotgun metagenomic sequencing. Insufficient DNA for shotgun metagenomic sequencing was extracted from soil samples from Komsomolets at a depth of 30–50 cm, so soils at this depth from unvegetated islands are solely represented by soil samples collected from Graham Bell.

	Unvegetated 0–2 cm	Unvegetated 30–50 cm	Vegetated 0–2 cm	Vegetated 30–50 cm
ARGs				
MLS	45	27	34	40
aminocoumarin	2	0	0	1
aminoglycoside	54	36	32	35
bacitracin	47	28	34	29
beta-lactam	61	26	45	48
diaminopyrimidine	13	1	15	13
fluoroquinolone	79	97	49	43
fosmidomycin	7	1	9	2
glycopeptide	217	164	110	123
multidrug	467	269	239	271
mupirocin	13	18	7	8
nucleoside	9	15	2	3
peptide	40	15	24	23
phenicol	30	27	18	24
pleuromutilin	36	30	19	28
polyamine:peptide	0	0	0	2
rifamycin	4	2	1	6
sulfonamide	33	41	18	15
tetracenomycin_C	17	32	3	1
tetracycline	85	111	66	50
triclosan	6	0	9	12
unclassified	74	73	65	56
	1339	1013	799	833

Supplementary Table 21. Effects of vegetation cover and soil depth on virome richness and Shannon index. Indices were calculated based on count per million (CPM) values and the genes classified as viruses. Values are presented as means ± standard deviation (n = 3). Statistical significance was assessed using linear mixed-effects models with vegetation (*v*) and soil depth (*s*) as fixed factors, their interaction *(vs*). *T*-values indicate effect direction and magnitude (positive/negative relationships). Unvegetated: Graham Bell (G), Komsomolets (K); Vegetated: October Revolution (O), Vize Island (V). Significant values are in bold. Data are from shotgun metagenomic sequencing. Insufficient DNA for shotgun metagenomic sequencing was extracted from soil samples from Komsomolets at a depth of 30–50 cm, so soils at this depth from unvegetated islands are solely represented by soil samples collected from Graham Bell.

	Richness	Shannon index
0–2 cm		
G	24,285±6,473	9.34±0.30
K	25,975±1,763	9.52±0.08
O	14,894±3,548	8.69±0.14
V	3,943±2,611	8.72±0.14
30–50 cm		
G	14,882±8,329	8.60±0.74
O	11,574±1,292	6.40±0.43
V	5,148±3,349	7.47±0.62
	**T_v = -4.60 P_v < 0.001**	T_v = -1.86 P_v = 0.14
	**T_s = -3.36 P_s = 0.004**	**T_s = -2.26 P_s = 0.037**
	T_{vs} = 0.40 P_{vs} = 0.70	**T_{vs} = -2.21 P_{vs} = 0.041**

Supplementary Table 22. Effects of vegetation cover and soil depth on the 15 most abundant virus phyla (counts per million, CPM). Values are presented as means ± standard deviation (n = 3). Statistical significance was assessed using linear mixed-effects models with vegetation (*v*) and soil depth (*s*) as fixed factors, their interaction (*vs*). *T*-values indicate effect direction and magnitude (positive/negative relationships). Unvegetated: Graham Bell (G), Komsomolets (K); Vegetated: October Revolution (O), Vize Island (V). Significant values are in bold. Data are from shotgun metagenomic sequencing. Insufficient DNA for shotgun metagenomic sequencing was extracted from soil samples from Komsomolets at a depth of 30–50 cm, so soils at this depth from unvegetated islands are solely represented by soil samples collected from Graham Bell.

	Unclassified	Uroviricota	Nucleocytoviricota	Preplasmiviricota	Cressdnaviricota	Phixviricota
0–2 cm						
G	424±106	3,552±1,245	16.7±3.1	1.6±0.4	4.5±2.1	0.2±0.1
K	763±85	4,786±678	38.2±1.5	7.7±5.2	11.3±3.9	0.3±0.3
O	260±46	3,566±1,037	30.4±10.6	2.7±3.6	4.4±2.6	1.9±1.9
V	146±32	1,542±94	10.0±12.0	0.7±1.0	0.1±0.1	0.7±0.9
30–50 cm						
G	410±20	4,044±993	24.3±10.1	17.5±23.9	2.1±3.6	0.3±0.1
O	192±108	7,867±9,250	1.4±1.1	1.0±1.7	0.1±0.1	7.4±5.9
V	142±39	2,028±413	2.57±1.33	3.0±5.1	4.2±4.0	0.1±0.1
	T_v= -2.27 P_v = 0.15	T_v= -1.68 P_v = 0.21	T_v= -0.59 P_v = 0.62	T_v= -1.60 P_v = 0.13	T_v= -2.08 P_v = 0.20	T_v= 0.55 P_v = 0.61
	T_s= -0.45 P_s = 0.66	T_s= 0.36 P_s = 0.73	T_s= 0.58 P_s = 0.57	T_s= -0.52 P_s = 0.61	T_s= -1.66 P_s = 0.12	T_s= 0.06 P_s = 0.95
	T_{vs}= -0.13 P_{vs}= 0.90	T_{vs}= -0.52 P_{vs}= 0.61	**T_{vs}= -2.45 P_{vs}= 0.026**	T_{vs}= 0.54 P_{vs}= 0.60	T_{vs}= 1.32 P_{vs}= 0.20	T_{vs}= 0.43 P_{vs}= 0.67

	Taleaviricota	Cossaviricota	Peploviricota	Artverviricota	Hofneiviricota	Pisuviricota
0–2 cm						
G	0.5±0.4	0.0±0.0	0.1±0.1	0.1±0.1	0.2±0.0	1.9±0.3
K	3.7±1.5	0.0±0.0	0.3±0.4	0.7±0.7	0.5±0.1	2.1±0.5
O	0.0±0.0	0.0±0.0	0.2±0.3	0.1±0.1	1.0±0.7	0.4±0.3
V	0.1±0.1	0.0±0.0	0.1±0.2	0.5±0.5	0.2±0.2	1.0±0.5
30–50 cm						
G	0.1±0.1	0.0±0.0	0.1±0.1	0.3±0.3	0.3±0.1	2.1±0.9
O	0.0±0.0	3.9±6.7	0.0±0.0	0.0±0.0	0.3±0.2	0.6±0.9
V	0.0±0.0	0.1±0.1	2.1±3.0	0.1±0.2	5.1±7.2	0.3±0.2
	T_v= -1.29 P_v = 0.32	T_v= -0.06 P_v = 0.96	T_v= -0.20 P_v = 0.84	T_v= 0.40 P_v = 0.71	T_v= 0.78 P_v = 0.45	**T_v= -3.88 P_v = 0.014**
	**T_s= -3.60 P_s = 0.004**	T_s= 0.00 P_s = 1.00	T_s= -0.65 P_s = 0.53	T_s= 0.62 P_s = 0.55	T_s= -0.22 P_s = 0.83	T_s= 0.14 P_s = 0.89
	**T_{vs}= 2.97 P_{vs}= 0.011**	T_{vs}= 0.91 P_{vs}= 0.38	T_{vs}= 0.46 P_{vs}= 0.65	T_{vs}= -1.34 P_{vs}= 0.20	T_{vs}= 0.04 P_{vs}= 0.97	T_{vs}= 1.04 P_{vs}= 0.31

Supplementary Table 23. Effects of vegetation cover and soil depth on the 15 most abundant virus families (CPMs). Values are presented as means ± standard deviation (n = 3). Statistical significance was assessed using linear mixed-effects models with vegetation (*v*) and soil depth (*s*) as fixed factors, their interaction (*vs*). *T*-values indicate effect direction and magnitude (positive/negative relationships). Unvegetated: Graham Bell (G), Komsomolets (K); Vegetated: October Revolution (O), Vize Island (V). Significant values are in bold. Data are from shotgun metagenomic sequencing. Insufficient DNA for shotgun metagenomic sequencing was extracted from soil samples from Komsomolets at a depth of 30–50 cm, so soils at this depth from unvegetated islands are solely represented by soil samples collected from Graham Bell.

	Unclassified	Autographiviridae	Schitoviridae	Drexlerviridae	Straboviridae	Herelleviridae	Mimiviridae	Phycodnaviridae
<u>0–2 cm</u>								
G	3,910±1,309	4.9±3.1	1.5±0.2	26.0±2.0	5.4±2.2	16.8±9.2	10.3±2.0	4.1±1.4
K	5,452±728	15.8±2.7	9.9±3.7	20.4±7.1	19.3±8.0	17.2±6.4	13.1±1.5	12.8±0.9
O	53,757±1,060	19.4±12.7	12.5±9.3	10.7±3.0	7.1±1.1	8.6±2.8	12.2±8.5	11.1±5.2
V	1,661±102	5.1±1.5	0.4±0.5	5.9±0.6	5.3±1.8	1.1±0.3	3.2±2.9	3.0±4.3
<u>30–50 cm</u>								
G	4,376±944	5.1±2.7	1.7±2.0	25.1±7.9	6.4±4.1	6.6±5.7	10.6±1.4	7.3±6.0
O	7,836±8,995	86.7±144	109±188	10.4±8.0	3.2±0.5	2.1±2.1	0.2±0.2	0.7±0.6
V	2,147±407	5.4±4.2	0.8±1.0	6.6±3.2	3.7±2.5	1.6±0.6	2.1±1.1	0.4±0.4
	T_v= -1.85 P_v = 0.19	T_v= 0.19 P_v = 0.86	T_v= 0.12 P_v = 0.91	**T_v= -3.64 P_v = 0.024**	T_v= -0.85 P_v = 0.49	**T_v= -3.90 P_v = 0.009**	T_v= -1.35 P_v = 0.24	T_v= -0.27 P_v = 0.81
	T_s= 0.28 P_s = 0.78	T_s= -0.63 P_s = 0.54	T_s= -0.35 P_s = 0.73	T_s= 0.38 P_s = 0.71	T_s= 0.09 P_s = 0.92	**T_s= -3.00 P_s = 0.009**	T_s= -0.34 P_s = 0.74	T_s= 0.51 P_s = 0.62
	T_{vs}= -0.48 P_{vs}= 0.64	T_{vs}= -0.33 P_{vs}= 0.74	T_{vs}= -0.75 P_{vs}= 0.47	T_{vs}= -0.26 P_{vs}= 0.80	T_{vs}= -1.07 P_{vs}= 0.30	T_{vs}= 1.71 P_{vs}= 0.11	T_{vs}= -1.31 P_{vs}= 0.21	T_{vs}= -2.02 P_{vs}= 0.06

	Kyanoviridae	Demerecviridae	Iridoviridae	Ackermannviridae	Tectiviridae	Vilmaviridae	Microviridae	Zobellviridae
<u>0–2 cm</u>								
G	2.3±0.8	4.8±2.4	0.7±0.1	1.5±0.9	0.3±0.3	1.1±1.2	0.2±0.1	2.5±1.0
K	8.3±4.1	5.1±2.5	3.9±1.6	3.4±1.6	0.3±0.3	3.2±2.9	0.3±0.3	0.4±0.1
O	1.9±1.3	4.2±1.7	1.4±0.9	1.8±0.4	0.2±0.2	0.9±1.2	1.9±1.9	6.0±5.4
V	1.1±0.3	2.3±0.5	2.4±3.6	1.2±0.3	0.1±0.1	4.8±4.4	0.7±0.9	0.7±0.1
<u>30–50 cm</u>								
G	17.4±28.0	11.8±8.7	6.0±5.2	1.5±1.7	11.6±17.0	0.8±0.7	0.3±0.1	0.4±0.7
O	1.1±0.3	0.6±0.4	0.0±0.0	3.7±2.4	0.0±0.0	0.0±0.0	7.4±5.9	1.1±1.2
V	2.2±1.2	0.8±0.1	0.1±0.1	0.6±0.4	0.0±0.0	1.2±1.1	0.1±0.1	0.1±0.1
	T_v= -1.37 P_v = 0.31	T_v= -0.63 P_v = 0.53	T_v= -0.28 P_v = 0.78	T_v= -0.78 P_v = 0.50	T_v= -0.68 P_v = 0.51	T_v= -0.48 P_v = 0.67	T_v= 0.56 P_v = 0.61	T_v= 0.92 P_v = 0.42
	T_s= -0.91 P_s = 0.38	**T_s= 2.76 P_s = 0.013**	T_s= 1.89 P_s = 0.077	T_s= -0.38 P_s = 0.71	**T_s= 7.79 P_s < 0.001**	T_s= -0.77 P_s = 0.45	T_s= 0.06 P_s = 0.95	T_s= -0.78 P_s = 0.45
	T_{vs}= 1.21 P_{vs}= 0.25	**T_{vs}= -2.97 P_{vs}= 0.008**	**T_{vs}= -2.28 P_{vs}= 0.036**	T_{vs}= 0.78 P_{vs}= 0.45	**T_{vs}= -0.93 P_{vs}< 0.001**	T_{vs}= -0.04 P_{vs}= 0.97	T_{vs}= 0.43 P_{vs}= 0.67	T_{vs}= -0.53 P_{vs}= 0.60

Supplementary Table 24. Raw read counts and the number of protein-coding genes classified using the eggNOG, CAZy, and NCyc databases of the surface soils (0–2 cm). Statistical differences were assessed using the Kruskal-Wallis test (soil depth), with results reported as χ^2 and *P*-values. Data are from shotgun metagenomic sequencing.

Sample	Soil depth (cm)	Vegetation	Island	Raw reads	eggNOG	CAZy	NCyc
ACE1	0–2	Vegetated	Vize Island	25,433,791	19,457,744	989,235	133,684
ACE4	0–2	Vegetated	Vize Island	18,605,974	14,539,788	753,357	103,460
ACE10	0–2	Vegetated	Vize Island	25,268,627	18,973,200	862,713	122,604
ACE18	0–2	Unvegetated	Graham Bell	33,587,381	27,608,572	1,419,863	188,020
ACE21	0–2	Unvegetated	Graham Bell	59,989,133	49,218,158	2,531,945	336,157
ACE24	0–2	Unvegetated	Graham Bell	59,726,492	49,587,008	2,549,158	349,363
ACE31	0–2	Unvegetated	Komsomolets	44,642,053	37,691,404	2,071,507	255,103
ACE33	0–2	Unvegetated	Komsomolets	38,305,810	32,504,263	1,900,700	227,126
ACE35	0–2	Unvegetated	Komsomolets	45,829,624	39,619,985	2,250,971	292,822
ACE64	0–2	Vegetated	October Revolution	23,787,873	18,774,556	961,775	129,364
ACE66	0–2	Vegetated	October Revolution	21,540,769	17,186,960	908,436	119,417
ACE68	0–2	Vegetated	October Revolution	22,430,702	17,910,863	957,192	122,314
					**χ^2 = 8.31 *P* = 0.003**		

Supplementary Table 25. Raw read counts and the number of protein-coding genes classified using the eggNOG, CAZy, and NCyc databases of the deeper soils (30–50 cm). Statistical differences were assessed using the Kruskal-Wallis test (soil depth), with results reported as χ^2 and *P*-values. Data are from shotgun metagenomic sequencing.

Sample	Soil depth (cm)	Vegetation	Island	Raw reads	eggNOG	CAZy	NCyc
ACE3	30–50	Vegetated	Vize Island	12,935,569	10,162,793	475,617	75,137
ACE6	30–50	Vegetated	Vize Island	28,981,348	22,846,117	1,096,203	179,729
ACE12	30–50	Vegetated	Vize Island	14,010,824	11,860,989	570,690	103,773
ACE23	30–50	Unvegetated	Graham Bell	38,827,230	33,765,774	1,702,498	252,803
ACE26	30–50	Unvegetated	Graham Bell	41,296,091	37,085,388	1,689,899	284,463
ACE27	30–50	Unvegetated	Graham Bell	57,103,413	49,358,658	2,323,075	384,586
ACE70	30–50	Vegetated	October Revolution	13,350,072	10,951,599	549,833	79,406
ACE71	30–50	Vegetated	October Revolution	11,193,987	8,991,033	490,487	67,209
ACE72	30–50	Vegetated	October Revolution	2,654,410	2,194,656	98,125	14,348
				**χ^2 = 5.40 *P* = 0.020**			

Supplementary Table 26. Summary of Analysis performed on contigs, and MAGs derived from the shotgun data. BGCs: Biosythetic gene clusters; ARGs: antimicrobial resistance genes.

Shotgun	Soil genetic potential	Microbial taxonomy and abundance	Viral taxonomy and abundance	Linear discriminant analysis	BGCs and ARGs
Contigs (after assembly with MEGAHIT)	X	X	X		
MAGs (high-quality: >87% completeness and <10% contamination)				X	X

Supplementary Results

1. Effects of vegetation and soil depth on the eggNOG categories

The abundance (counts per million, CPM) of gene-coding classes within the following super-categories were not significantly affected by vegetation cover: CPS: cell control, division, and chromosome partitioning (D), cell wall/membrane/envelope biogenesis (M), cell motility (N), post-translational modification (O), signal transduction mechanisms (T), intracellular secretion and vesicular transport (U), and defence mechanisms (V); IPS: chromatin structure and dynamics (B), translation, ribosomal structure, and biogenesis (J), and transcription (K); and MBM: energy production and conversion (C), amino acid transport and metabolism (E), nucleotide transport and metabolism (F), carbohydrate transport and metabolism (G), coenzyme transport and metabolism (H), lipid transport and metabolism (I), inorganic ion transport and metabolism (P), and secondary metabolite biosynthesis, transport, and catabolism (Q). However, on vegetated islands their abundance was higher at 30–50 cm soil depth (Table 4; Supplementary Table 8–10) while on unvegetated islands it was higher at 0–2 cm depth.

2. Metabolic genes identified in metagenome-assembled genomes (MAGs) from unvegetated soil (0–2 cm depth)

We identified seven indicator taxa corresponding to the following bins: 525 (Bacteroidota, Chitinophagaceae), 479 (Pseudomonadota, *Rhodopila*), 887 (Pseudomonadota, Steroidobacteraceae), 754 (Chloroflexota, *JANWJD01*), 8 (Actinomycetota, *Rhodococcus*), 587 (Pseudomonadota, Steroidobacteraceae), and 296 (Eremiobacterota, Baltobacteraceae). All these organisms were considered novel species (ANI < 95%).

Carbon cycle: Among the selected MAGs, MAGs 587, 887, 479, 754, and 8 contained genes encoding Rubisco forms I, IV, or both—key enzymes in the Calvin–Benson–Bassham (CBB) cycle for CO_2 fixation. These MAGs also harboured *sgdh* and *smdh*, which encode sorbitol and mannitol dehydrogenases, respectively, enzymes involved in sugar alcohol degradation. Additionally, MAGs 479, 296, 754, and 8 possessed *coxL*, *coxM*, and *coxS* genes, encoding the large, medium, and small subunits of carbon monoxide dehydrogenase (CODH), an enzyme complex that oxidizes CO and contributes to C fixation and energy generation under certain conditions. The *sfh* gene, encoding soluble fumarate hydratase, an enzyme of the tricarboxylic acid (TCA) cycle, was detected in MAGs 887 and 587. No genes associated with *madA* or *madB* (involved in malate degradation), *fmtF* (formyltransferase), or *fae* (formaldehyde-activating enzyme) were found in the selected MAGs. No C-related genes were identified in bin 525.

Nitrogen cycle: None of the selected MAGs contained *nifD*, *nifH*, or *nifK*, which encode the subunits of nitrogenase, the enzyme complex responsible for atmospheric nitrogen (N_2) fixation. Regarding nitrification, genes associated with nitrite oxidation—*nxrA* and *nxrB*, encoding nitrite oxidoreductase—were not detected in any of the MAGs. Similarly, genes related to nitrate reduction, such as *narH* (nitrate reductase subunit) and *narG* (catalytic subunit of nitrate reductase), were mostly absent, with the exception of *narG*, which was found only in MAG 8. In the denitrification pathway, genes involved in nitrite and nitric oxide reduction were detected in a few MAGs. Specifically, *nirD*, which is part of the *nirBD* operon encoding enzymes involved in the reduction of nitrite (NO_2^-) to ammonium (NH_4^+) via the dissimilatory nitrate reduction to ammonium (DNRA) pathway, was present in MAGs 479 and 8. Only MAG 479 contained *norB*, encoding subunits of nitric oxide reductase, which catalyses the conversion of NO to nitrous oxide (N_2O). However, none of the MAGs harboured *nosZ* or *nosD*, which are essential for the final step of denitrification—the reduction of N_2O to N_2—indicating the presence of an incomplete denitrification pathway.

Respiration: The *ccoP* gene, part of the high-affinity *cbb*$_3$-type cytochrome *c* oxidase complex, was found only in MAG 587, while *ccoN* and *ccoO* were absent from all MAGs, suggesting an incomplete complex. Genes encoding the low-affinity oxidase, *cydA* and *cydB*, were detected in MAGs 887, 587, 479, and 8. The genes *cyoA*, *cyoD*, and *cyoE*—the latter notably involved in heme oxygen biosynthesis—as well as *coxA* and *coxB*, which encode subunits of the *aa*$_3$-type cytochrome *c* oxidase, were present in all MAGs.

Sulphur cycle: The *sat* gene, which encodes sulphate adenylyltransferase—an enzyme involved in the activation of sulphate to adenosine 5'-phosphosulphate (APS) in the first step of sulphate reduction—was found only in MAG 754. No genes associated with dissimilatory sulphate reduction (*aprA*, *dsrA*, *dsrB*), sulphur oxidation via

flavocytochrome (*fccB*), or the Sox pathway (*soxY*, *soxC*, *soxB*) were detected in any of the MAGs. The *sqr* gene, encoding sulphide:quinone oxidoreductase, which catalyzes the oxidation of hydrogen sulphide (H_2S) to elemental sulphur (S^0), was identified in MAGs 8 and 479. The *sdo* gene, encoding sulphur dioxygenase—a key enzyme in the oxidation of persulphide to sulphite (SO_3^{2-})—was found in MAGs 587, 479, and 754.

3. Metabolic genes identified in MAGs from unvegetated soil (30–50 cm depth)

Seven indicator taxa were identified in this soil depth range, corresponding to the following bins: 720 (*Eremiobacterota*, *Baltobacteraceae*), 122 (*Chloroflexota*, JAJPKF01), 40 (*Acidobacteriota*, *Acidobacteriaceae*), 872 (*Eremiobacterota*, *Baltobacteraceae*), 564 (*Eremiobacterota*, *Baltobacteraceae*), 620 (*Chloroflexota*, UBA8260), and 795 (*Eremiobacterota*, *Baltobacteraceae*). All these organisms have ANI values below 95%.

Carbon cycle: MAGs 795 and 122 encoded Rubisco forms I and IV, indicating potential for CO_2 fixation through the Calvin–Benson–Bassham cycle. The genes *coxL*, *coxM*, and *coxS*—encoding subunits of CODH—were present in MAGs 872, 720, 795, 564, and 122, suggesting the capability for CO oxidation. The genes *sgdh*, *smdh*, *sfh*, *madA*, *madB*, *fmtF*, and *fae* were not detected in any of the MAGs analysed. **Nitrogen cycle:** No MAGs contained *nifD*, *nifH*, or *nifK* (N_2 fixation), *nxrA* or *nxrB* (nitrite oxidation), *narG* or *narH* (nitrate reduction), *nirK* or *nirS* (nitrite reduction via denitrification), *norB*, *nosZ*, or *nosD* (downstream denitrification), or *nrfH* (DNRA pathway). Only *nirD*, associated with the DNRA pathway (nitrite reduction to ammonium), was identified in MAGs 564, 122, and 40. The gene *norC*, encoding a subunit of nitric oxide reductase, was detected exclusively in MAG 40.

Respiration: The *ccoP* gene, encoding a component of the high-affinity cbb_3-type cytochrome c oxidase, was identified in MAGs 872, 720, 795, and 40, whereas *ccoN* was not detected in any MAGs, indicating an incomplete oxidase complex. The low-affinity oxidase genes *cydA* and *cydB* were present in MAGs 872, 720, 795, and 564. The *cyoE* gene—associated with heme O biosynthesis—was identified in all MAGs, as were *coxA* and *coxB* (subunits of aa_3-type cytochrome c oxidase); however, *cyoA* and *cyoD* were also present throughout.

Sulphur cycle: The *sat* gene, encoding sulphate adenylyltransferase (activating sulphate to APS in the first step of sulphate reduction), was found only in MAG 122. Genes associated with dissimilatory sulphate reduction (*aprA*, *dsrA*, *dsrB*) or flavocytochrome-mediated sulphur oxidation (*fccB*) were not detected in any MAG. Sox pathway genes (*soxY*, *soxC*, *soxB*) and *sqr* (sulphide:quinone oxidoreductase) were identified in MAG 795, while *sdo* (sulphur dioxygenase, for persulphide oxidation) was present in MAGs 795 and 122.

4. Metabolic genes identified in MAGs from vegetated soil (0–2 cm depth)

We identified seven indicator taxa, corresponding to the following bins: 95 (*Bacteroidota*, *Kapaibacteriaceae*), 193 (*Actinomycetota*, *Acidimicrobiia*), 562 (*Actinomycetota*, *Ilumatobacter*), 785 (*Pseudomonadota*, *Qipengyuania*), 554 (*Bacteroidota*, *Chryseolinea*), 884 (*Actinomycetota*, JACDCJ01), and 836 (*Bacteroidota*, UBA955). All these organisms had ANI values below 95%.

Carbon cycle: The genes *coxL*, *coxM*, and *coxS*, associated with carbon monoxide oxidation, were identified in MAGs 562, 193, and 884. Rubisco forms I and IV were detected in MAGs 193, 884, 562, 785, and 554, indicating potential for autotrophic CO_2 fixation via the Calvin–Benson–Bassham cycle. The genes *sgdh* and *sfh* were found exclusively in MAG 785, while *smdh* was detected in MAG 562. The gene *fmtF* was not detected, *fae* was found in MAG 562, and *madA* and *madB* were absent from all MAGs.

Nitrogen cycle: The genes *nifD*, *nifH*, *nifK*, *nosZ*, *nirK*, *nirD*, *narG*, *narH*, *norB*, *nrfH*, and *norC* were not detected. However, *nxrA*, *nxrB*, and *nosD*—associated with nitrite oxidation and nitrous oxide reduction—were present in MAG 95.

Respiration: The genes *ccoP*, *ccoO*, and *ccoN*, encoding subunits of the high-affinity *cbb₃*-type cytochrome *c* oxidase, were identified in MAGs 936 and 95. The gene *cydA*, related to the low-affinity terminal oxidase, was found in MAG 554, while *cydB* was not detected in any MAGs. The *cyoE* gene, involved in heme O biosynthesis, was present in MAGs 785, 836, 95, 554, and 193; however, *cyoA* and *cyoD* were absent from all MAGs. Finally, *coxA* and *coxB*, subunits of the *aa₃*-type cytochrome *c* oxidase, were detected in MAGs 193, 884, 562, 95, and 785.

Sulphur cycle: No genes associated with dissimilatory sulphate reduction (*sat*, *aprA*, *dsrA*, *dsrB*), flavocytochrome-mediated sulphur oxidation (*fccB*), the Sox pathway (*soxY*, *soxC*, *soxB*), or sulphide oxidation (*sqr*) were detected. Only the *sdo* gene, encoding sulphur dioxygenase involved in persulphide oxidation, was found in MAGs 836, 95, and 554.

5. <u>Metabolic genes identified in MAGs from vegetated soil (30–50 cm depth)</u>

Seven indicator taxa were identified in this soil depth range, corresponding to the following bins: 536 (*Planctomycetota*, *Pirellulaceae*), 160 (*Pseudomonadota*, *Thiobacillus*), 797 (*Actinomycetota*, *Ilumatobacteraceae*), 492 (*Acidobacteriota*, *Pyrinomonadaceae*), 810 (*Pseudomonadota*, *Cypionkella*), 750 (*Acidobacteriota*, *Thermoanaerobaculia*), and 699 (*Bacteroidota*, *Flavitalea*). All of these MAGs exhibited ANI values below 95%.

Carbon cycle: The *coxL*, *coxM*, and *coxS* genes, encoding subunits of CODH, were found in MAGs 810, 536, 797, and 750. Rubisco forms I and IV were detected in MAGs 810, 160, 536, and 750, suggesting potential for autotrophic CO_2 fixation. The genes *sgdh* and *sfh* were found in MAGs 810 and 160, while *smdh* was not detected in any MAGs. The genes *fmtF* and *fae* were present in MAG 536, whereas *madA* and *madB* were absent from all MAGs.

Nitrogen cycle: The genes *nifD*, *nifH*, *nifK*, and *nosZ* were not detected in any of the selected MAGs. However, *nxrA* and *nxrB* were found in MAG 536, while *narG* and *narH* were identified exclusively in MAG 160. Genes *nirK*, *nirD*, and *norB* were present in MAGs 810, 160, and 536. *NosD* was detected only in MAG 160, and *nrfH*, associated with DNRA, was found in MAG 750. The *norC* gene was present exclusively in MAGs 810 and 160.

Respiration: The *ccoP*, *ccoO*, and *ccoN* genes, components of the high-affinity *cbb₃*-type cytochrome *c* oxidase, were identified in MAGs 810 and 160, while *ccoO* was also

found in MAG 536. The gene *cydA*, encoding part of the low-affinity cytochrome *bd* oxidase, was present in MAG 536, but *cydB* was not detected in any MAG. The *cyoE* gene, involved in heme O biosynthesis, was detected in all MAGs, whereas *cyoA* and *cyoD* were present only in MAG 160. Finally, *coxA* and *coxB*, subunits of the aa_3-type cytochrome *c* oxidase, were found in MAGs 810, 160, 536, 797, 750, and 492.

Sulphur cycle: Genes involved in sulphur oxidation and reduction were primarily found in MAG 160, which encoded *sat*, *aprA*, *dsrA*, *dsrB*, *soxY*, *soxB*, and *sqr*. Both *sqr* and *sdo* were found in MAG 536, while *sdo* alone was detected in MAGs 699 and 492.
