# Supplementary material for: Metagenomic insights into viral and microbial genes of Russian High-Arctic soil microbiomes
Source: Commun Biol. 2026 Apr 15;9:819. doi: 10.1038/s42003-026-10050-0 (PMC13269706; doi:10.1038/s42003-026-10050-0)
Supplement: Supplementary file 19 — Description of Additional Supplementary Files [file 42003_2026_10050_MOESM19_ESM.pdf]

Description of Additional Supplementary Files

File name: **Supplementary Data 1.**
Description: Extracted geographic coordinates used to generate the sampling map shown in **Figure 1.**

File name: **Supplementary Data 2.**
Description: Non-metric multidimensional scaling (NMDS) output used for **Figure 2A.**

File name: **Supplementary Data 3.**
Description: Relative abundance of microbial phyla (TPM) used for **Figure 2B.**

File name: **Supplementary Data 4.**
Description: Non-metric multidimensional scaling (NMDS) output used for **Figure 2C.**

File name: **Supplementary Data 5.**
Description: Relative abundance of microbial genera (TPM) used for **Figure 2D.**

File name: **Supplementary Data 6.**
Description: Bray–Curtis distance matrix of functional genetic potential used for **Figure 3.**

File name: **Supplementary Data 7.**
Description: Raw metabolic summary data used for **Figure 4.**

File name: **Supplementary Data 8.**
Description: Information on metagenome-assembled genomes (MAGs) used for **Figure 4B.**

File name: **Supplementary Data 9.**
Description: DeepBGC output used for **Figures 5A–D.**

File name: **Supplementary Data 10.**
Description: DeepBGC output used for **Figure 5C.**

File name: **Supplementary Data 11.**
Description: Phylum-level relative abundance (CPM) used for **Figure 6A.**

File name: **Supplementary Data 12.**
Description: Family-level relative abundance (CPM) used for **Figure 6B.**

File name: **Supplementary Data 13.**
Description: Linear discriminant analysis (LDA) output used for **Supplementary Figure 5.**

File name: **Supplementary Data 14.**
Description: Bray–Curtis distance matrix of viral community structure used for **Supplementary Figure 6.**

File name: **Supplementary Data 15.**
Description: Relative abundance of phyla (CPM) obtained using the **GTDB-Tk** database.

File name: **Supplementary Data 16.**
Description: Relative abundance of genera (CPM) obtained using the **GTDB-Tk** database.

File name: **Supplementary Data 16.**
Description: Output generated by **MetaBAT2** for binning of assembled contigs.

File name: **Supplementary Data 17.**
Description: Output generated by **MetaBAT2 +** Taxonomic classification using **GTDB-Tk.**
