# Supplementary material for: Metagenomic insights into viral and microbial genes of Russian High-Arctic soil microbiomes
Source: Commun Biol. 2026 Apr 15;9:819. doi: 10.1038/s42003-026-10050-0 (PMC13269706; doi:10.1038/s42003-026-10050-0)
Supplement: Supplementary file 21 — Transparent Peer Review file [file 42003_2026_10050_MOESM21_ESM.pdf]

nature portfolio

Peer Review File

Metagenomic insights into viral and microbial genes of Russian High-Arctic soil microbiomes

Corresponding Author: Dr Beat Frey

This file contains all reviewer reports in order by version, followed by all author rebuttals in order by version.

Version 0:

Reviewer comments:

Reviewer #1

(Remarks to the Author)
This manuscript presents a valuable dataset: the first shotgun metagenomic survey of soil microbiomes and viruses from multiple High-Arctic islands with contrasting vegetation covers. The integration of taxonomic, functional, BGC/ARG, MAG-based, and virus analyses is impressive. However, despite strong metagenomic datasets, the manuscript presently suffers from outdated software/databases and CDS-based analysis. The paper would benefit from substantial revision before it can be considered for publication.

A major methodological weakness is the extensive use of outdated, around 5-year-old bioinformatic tools and reference databases. This may be a critical issue for a 2025 metagenomics paper. For example, Trimmomatic v0.39 (2021), MetaGeneMark v3.25 (2020), MEGAHIT 1.2 (2019), eggNOG v5 (2020), Kaiju v1.7 (2021), and CAZy 2017 release. The updated bioinformatic tools and databases may introduce bias for the taxonomic and many functional interpretations.

Another flaw lies in the decision to infer taxonomic composition and diversity directly from coding DNA sequences (CDSs) predicted from assembled contigs. CDS-based taxonomy may also be biased, as coding sequence composition varies by organism, genome size, GC content, and assembly efficiency. The authors are encouraged to test the rRNA- or marker-gene–based profiles (e.g., mOTUs, singleM or phyloFlash) for the diversity estimates. This likely explains why taxonomic differences among treatments were weak. The discussion of functional profiles may also acknowledge this limitation.

The manuscript presents a unique and valuable dataset that clearly deserves deeper and more rigorous scientific analysis to realize its full potential.

Reviewer #2

(Remarks to the Author)
The authors of this study assessed the role of vegetation and depth on microbial diversity in high Arctic soils. The study included four islands that captured both conditions and the authors collected deep metagenomic sequence data. There is a general paucity of soil microbial community data available across the circumpolar regions, so this data set provides crucial missing information and will be a great interest to the community. Beyond investigating the bacterial community, the authors also included an analysis of the viral communities which complements the investigation and provides even more needed information on Arctic soils.

The study tested if vegetation increased microbial taxonomic and functional diversity, but did not find evidence to support. Using MAGs, they tested if vegetation increased genes related to carbon and nitrogen cycling and reduced biosynthetic gene clusters and antimicrobial resistance genes. They found no differences in C & N cycling genes, contrary to their hypothesis. However, they did find more ARGs in unvegetated soils. Contrary to their hypothesis, vegetation did not have a significant effect on viral diversity. Consistent with similar systems, viral diversity is higher in surface soils.

1. The work and conclusions that the authors draw from the evidence are convincing. The manuscript could benefit from a greater acknowledgement of the sampling design limitations. With only two islands of each type sampled, the authors are limited in the generality of the conclusions. Requesting additional samples is not reasonable or feasible as sampling in

Arctic environments is extremely challenging. However, the quality of the results and discussion provide a generous amount of information.

2. To strengthen the conclusions, the study could include results on whether soil chemistry properties influence the functional gene potential. As the manuscript is written, the authors have not directly connected these, only through vegetation status and depth.

3. The statistical analyses are well-conducted and appropriate. The manuscript could be improved with a clearer description of the sampling design. A figure, included with the maps, would be helpful to visualize the samples, their sampling locations (including vegetation cover, depth, and island), and the quantities.

4. More clarity is needed on how the assemblies computed on pooled data or always on a per sample basis?

5. Related to the previous point, the description of the methods and interpretation of the results could be improved with more clearly stating which data was being used for each kind of analysis. At times it was difficult to know if the analysis was based only on the contigs vs the MAGs. Since the MAGs represent a more limited sample of the community, it is helpful to the reader to understand which is being used when.

6. The quantitative PCR results are very briefly summarized in section 3.1, but the manuscript does little to integrate these results in the other analysis or discussion. This creates confusion regarding their role in the study.

7. In the discussion (LL574-598), the authors suggest a role of thawing permafrost in driving some of the microbial patterns found in the deeper samples. Is there evidence that these regions are experiencing thaw events?

Reviewer #3

(Remarks to the Author)
I co-reviewed this manuscript with one of the reviewers who provided the listed reports. This is part of the Communications Biology initiative to facilitate training in peer review and to provide appropriate recognition for Early Career Researchers who co-review manuscripts.

Version 1:

Reviewer comments:

Reviewer #1

(Remarks to the Author)
I'm happy that the authors accepted my suggestions.

Reviewer #2

(Remarks to the Author)
The authors have addressed all of my previous comments with this revised manuscript.

I want to thank the authors for carefully addressing each of the previous comments. I found the response to reviews document particularly helpful. I appreciate the efforts the authors took to clarify the methods and analysis. I found the additional analysis added to the manuscript.

Reviewer #3

(Remarks to the Author)
I co-reviewed this manuscript with one of the reviewers who provided the listed reports. This is part of the Communications Biology initiative to facilitate training in peer review and to provide appropriate recognition for Early Career Researchers who co-review manuscripts.

Open Access This Peer Review File is licensed under a Creative Commons Attribution 4.0 International License, which permits use, sharing, adaptation, distribution and reproduction in any medium or format, as long as you give appropriate credit to the original author(s) and the source, provide a link to the Creative Commons license, and indicate if changes were made.
In cases where reviewers are anonymous, credit should be given to 'Anonymous Referee' and the source.
The images or other third party material in this Peer Review File are included in the article's Creative Commons license, unless indicated otherwise in a credit line to the material. If material is not included in the article's Creative Commons license and your intended use is not permitted by statutory regulation or exceeds the permitted use, you will need to obtain permission directly from the copyright holder.
To view a copy of this license, visit https://creativecommons.org/licenses/by/4.0/

Reviewer 1 report:

This manuscript presents a valuable dataset: the first shotgun metagenomic survey of soil microbiomes and viruses from multiple High-Arctic islands with contrasting vegetation covers. The integration of taxonomic, functional, BGC/ARG, MAG-based, and virus analyses is impressive. However, despite strong metagenomic datasets, the manuscript presently suffers from outdated software/databases and CDS-based analysis. The paper would benefit from substantial revision before it can be considered for publication.

Query 1. A major methodological weakness is the extensive use of outdated, around 5-year-old bioinformatic tools and reference databases. This may be a critical issue for a 2025 metagenomics paper. For example, Trimmomatic v0.39 (2021), MetaGeneMark v3.25 (2020), MEGAHIT 1.2 (2019), eggNOG v5 (2020), Kaiju v1.7 (2021), and CAZy 2017 release. The updated bioinformatic tools and databases may introduce bias for the taxonomic and many functional interpretations.

Answer 1. DONE. We understand that reporting the most up-to-date software versions is crucial. Therefore, we have carefully reviewed all the programs used in our analyses and noted that some versions were incorrectly indicated in the manuscript, while others were indeed the most current at the time of analysis (conducted between 2022 and 2023).

For example:

- **Trimmomatic v0.39 (2021)** was used; the newer version, v0.40, was only released recently (August 20, 2025).
- **MEGAHIT** v1.2.9 is currently at the latest available version; see https://github.com/voutcn/megahit/releases.
- **MetaGeneMark v3.25 (2020)** was incorrectly cited in the manuscript; the analyses were actually performed with v3.38, which is the current version (https://exon.gatech.edu/GeneMark/license_download.cgi). This has now been corrected in the manuscript (L265).
- **Functional annotation** was performed using eggnog-mapper v2.1.6 (released Oct. 6, 2021), which was the current version when the analyses began.
- **CAZy annotation** was conducted using scripts and databases from https://github.com/anwarMZ/CoMW, which have not been updated since 2019.
- **Kaiju** was used in version v1.10.0 instead of v1.7, released in November 2023 (https://anaconda.org/channels/bioconda/packages/kaiju/files). This has been corrected in the manuscript (L284).

Query 2. Another flaw lies in the decision to infer taxonomic composition and diversity directly from coding DNA sequences (CDSs) predicted from assembled contigs. CDS-based taxonomy may also be biased, as coding sequence composition varies by organism, genome size, GC content, and assembly efficiency. The authors are encouraged to test the rRNA- or marker-gene–based profiles (e.g., mOTUs, singleM or phyloFlash) for the diversity estimates. This likely explains why taxonomic differences among treatments were weak. The discussion of functional profiles may also acknowledge this limitation.

Answer 2. DONE. We agree with the reviewer that Kaiju may not be the most appropriate tool for this analysis. After evaluating several alternatives, we decided to use phyloFlash v3.4.2 together with the most up-to-date SILVA database (version 138.1). This has been specified in the Materials and Methods section (L285), and the updated alpha-diversity results are now included in Table S4 (below). As anticipated by the reviewer, this approach allows us to detect significant differences in the alpha-diversity, demonstrating that vegetation enhances microbial richness and Shannon index, particularly at greater soil depths. In addition, vegetation cover and soil depth significantly alters microbial community structure, as reported in the manuscript: "Vegetation cover and soil depth both significantly affected microbial community structure ($F_v = 22.12$; $P = 0.003$; $P_s = 4.59$; P = 0.002)." (L394-395)

	Richness	Shannon index
0–2 cm		
G	200±7	4.1±0.1
K	192±45	4.0±0.1
O	243±13	4.4±0.1
V	278±5	4.5±0.1
30–50 cm		
G	149±45	3.6±0.6
O	254±6	4.3±0.1
V	251±5	4.3±0.2
	**$T_v = 4.95$ $P_v = 0.003$**<br>**$T_s = -3.30$ $P_s = 0.004$**<br>**$T_{vs} = 2.14$ $P_{vs} = 0.048$**	**$T_v = 4.29$ $P_v = 0.035$**<br>**$T_s = -5.32$ $P_s < 0.001$**<br>**$T_{vs} = 3.62$ $P_{vs} = 0.003$**

The manuscript presents a unique and valuable dataset that clearly deserves deeper and more rigorous scientific analysis to realize its full potential.

Reviewer 2 report:

The authors of this study assessed the role of vegetation and depth on microbial diversity in high Arctic soils. The study included four islands that captured both conditions and the authors collected deep metagenomic sequence data. There is a general paucity of soil microbial community data available across the circumpolar regions, so this data set provides crucial missing information and will be a great interest to the community. Beyond investigating the bacterial community, the authors also included an analysis of the viral communities which complements the investigation and provides even more needed information on Arctic soils.

The study tested if vegetation increased microbial taxonomic and functional diversity, but did not find evidence to support. Using MAGs, they tested if vegetation increased genes related to carbon and nitrogen cycling and reduced biosynthetic gene clusters and antimicrobial resistance genes. They found no differences in C & N cycling genes, contrary to their hypothesis. However, they did find more ARGs in unvegetated soils. Contrary to their hypothesis, vegetation did not have a significant effect on viral diversity. Consistent with similar systems, viral diversity is higher in surface soils.

Query 3. The work and conclusions that the authors draw from the evidence are convincing. The manuscript could benefit from a greater acknowledgement of the sampling design limitations. With only two islands of each type sampled, the authors are limited in the generality of the conclusions. Requesting additional samples is not reasonable or feasible as sampling in Arctic environments is extremely challenging. However, the quality of the results and discussion provide a generous amount of information.

Answer 3. DONE. We appreciate the reviewer's recognition of these limitations, which are inherent to the remoteness of the area. Following this comment, we have included an explicit acknowledgment of this constraint in the Materials & Methods section. Now it reads: “Sampling locations were determined by the logistical constraints of the ACE expedition, including coordination with researchers from other disciplines and weather conditions. These constraints allowed only a short period (up to 6 hours) of fieldwork on these specific islands and precluded access to other potential sites.” (L142-146)

Query 4. To strengthen the conclusions, the study could include results on whether soil chemistry properties influence the functional gene potential. As the manuscript is written, the authors have not directly connected these, only through vegetation status and depth.

Answer 4. DONE. We have included a Redundancy Analysis (RDA) to evaluate the influence of soil physicochemical properties on the functional gene potential. The Material & Methods section now states: “To evaluate the influence of soil physico-chemical properties on functional gene potential, a Redundancy Analysis (RDA) was performed using the rda() function from the *vegan* package. The statistical significance was assessed via permutation tests using the anova.cca function with 999 permutations.” (Lines 343-347). The analysis yielded significant insights, showing that the functional gene structure was driven by pH at both soil depths and SOC content at the 0–2 cm layer (Table S8, below).

	pH	SOC	Clay	Silt
Entire dataset	$F = 0.71$; $P = 0.4$	$F = 0.46$; $P = 0.8$	$F = 0.02$; $P = 0.9$	$F = 0.13$; $P = 0.8$
0–2 cm depth	**$F = 4.76$; $P = 0.021$**	**$F = 4.13$; $P = 0.023$**	$F = 0.46$; $P = 0.7$	$F = 2.39$; $P = 0.1$
30–50 cm depth	**$F = 9.21$; $P = 0.010$**	$F = 1.40$; $P = 0.4$	$F = 0.30$; $P = 0.7$	$F = 3.23$; $P = 0.1$
Unvegetated islands	$F = 4.52$; $P = 0.053$	$F = 9.26$; $P = 0.055$	$F = 0.30$; $P = 0.7$	$F = 0.44$; $P = 0.6$
Vegetated islands	$F = 4.09$; $P = 0.057$	$F = 0.13$; $P = 0.6$	$F = 0.40$; $P = 0.4$	$F = 1.84$; $P = 0.37$

Query 5. The statistical analyses are well-conducted and appropriate. The manuscript could be improved with a clearer description of the sampling design. A figure, included with the maps, would be helpful to visualize the samples, their sampling locations (including vegetation cover, depth, and island), and the quantities.

Answer 5. **DONE**. We have replaced Figure 1 with a new version that directly includes the landscape and soil integrated into the map. We have also added the number of samples per island and the sampling locations. Please see the updated Figure 1 (below).

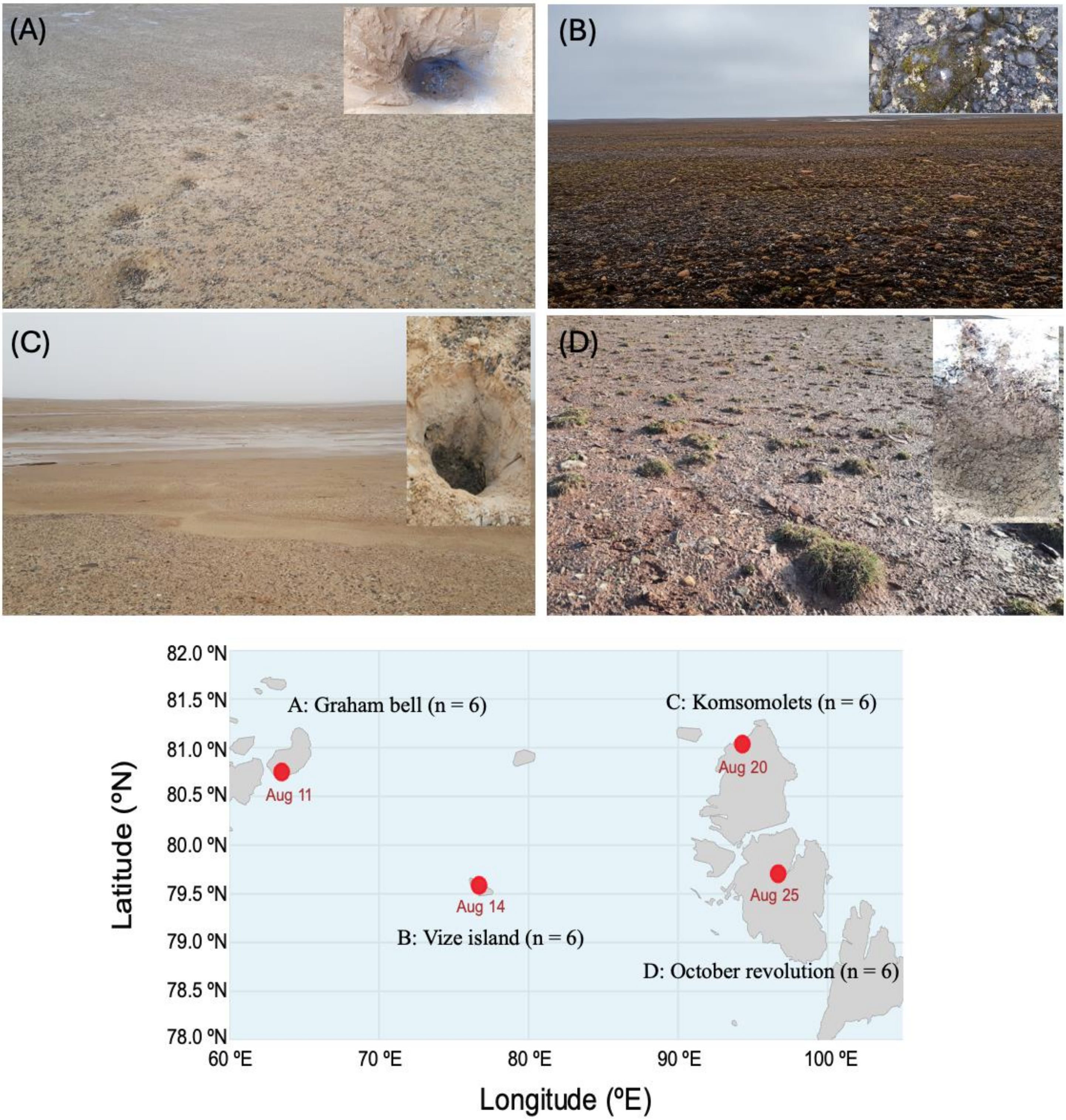

Query 6. More clarity is needed on how the assemblies computed on pooled data or always on a per sample basis?

Answer 6. **DONE**. We have clarified that all samples were analysed on a per-sample basis. The revised text now reads: “All samples were processed and assembled independently; no pooled assemblies were performed.” (L262-263).

Query 7. Related to the previous point, the description of the methods and interpretation of the results could be improved with more clearly stating which data was being used for each kind of analysis. At times it was difficult to know if the analysis was based only on the contigs vs the MAGs. Since the MAGs represent a more limited sample of the community, it is helpful to the reader to understand which is being used when.

Answer 7. **DONE**. We acknowledge the complexity of this assay, so we have included a new Table S3 summarizing the analyses performed on contigs and MAGs derived from the shotgun data. Please see the Table S3 below.

Shotgun	Genetic potential	Microbial taxonomy and abundance	Viral taxonomy and abundance	BGC, ARGs and linear discriminant analysis	BGCs and ARGs
Contigs (after assembly with MEGAHIT)	X	X	X		
MAGs (high-quality: >87% completeness and <10% contamination)				X	X

Query 8. The quantitative PCR results are very briefly summarized in section 3.1, but the manuscript does little to integrate these results in the other analysis or discussion. This creates confusion regarding their role in the study.

Answer 8. **DONE**. We agree with the reviewer that qPCR integration was insufficient. We have now integrated these results into the Discussion section. Now it reads “Notably, qPCR quantification revealed that 16S rRNA gene copy numbers were significantly reduced at greater soil depths, suggesting that while community composition may be resilient to vegetation cover, the bacterial abundance declines substantially with depth, likely reflecting depletion of labile carbon substrates below the active soil layer.” (L611-615).

Query 9. In the discussion (LL574-598), the authors suggest a role of thawing permafrost in driving some of the microbial patterns found in the deeper samples. Is there evidence that these regions are experiencing thaw events?

Answer 9. Thank you for this comment. Unfortunately, due to the remoteness of the site there are no long-term permafrost or ground-temperature monitoring data available, so thaw events cannot be documented directly. However, during fieldwork in August (the warmest period of the year), we measured soil temperature while excavating the profiles and consistently recorded values below +0.5 °C at 50 cm depth, indicating partially frozen soil. This suggests that the 50 cm horizon is located at, or very close to, the permafrost table and is seasonally affected by thawing, which underpins our interpretation of the deeper microbial patterns in the context of permafrost thaw.

Reviewer 1 report:

Query 1. I'm happy that the authors accepted my suggestions.

Answer 1. Thank you.

Reviewer 2 report:

Query 2. The authors have addressed all of my previous comments with this revised manuscript. I want to thank the authors for carefully addressing each of the previous comments. I found the response to reviews document particularly helpful. I appreciate the efforts the authors took to clarify the methods and analysis. I found the additional analysis added to the manuscript.

Answer 2. Thank you for your kind words.

Reviewer 3 report:

I co-reviewed this manuscript with one of the reviewers who provided the listed reports. This is part of the Communications Biology initiative to facilitate training in peer review and to provide appropriate recognition for Early Career Researchers who co-review manuscripts.
